# Elastic network model of learned maintained contacts to predict protein motion

**DOI:** 10.1371/journal.pone.0183889

**Published:** 2017-08-30

**Authors:** Ines Putz, Oliver Brock

**Affiliations:** Robotics and Biology Laboratory, Department of Computer Science and Electrical Engineering, Technische Universität Berlin, Berlin, Berlin, Germany; Koç University, TURKEY

## Abstract

We present a novel elastic network model, *lmc*ENM, to determine protein motion even for localized functional motions that involve substantial changes in the protein’s contact topology. Existing elastic network models assume that the contact topology remains unchanged throughout the motion and are thus most appropriate to simulate highly collective function-related movements. *lmc*ENM uses machine learning to differentiate breaking from maintained contacts. We show that *lmc*ENM accurately captures functional transitions unexplained by the classical ENM and three reference ENM variants, while preserving the simplicity of classical ENM. We demonstrate the effectiveness of our approach on a large set of proteins covering different motion types. Our results suggest that accurately predicting a “deformation-invariant” contact topology offers a promising route to increase the general applicability of ENMs. We also find that to correctly predict this contact topology a combination of several features seems to be relevant which may vary slightly depending on the protein. Additionally, we present case studies of two biologically interesting systems, Ferric Citrate membrane transporter FecA and Arachidonate 15-Lipoxygenase.

## Introduction

The function of proteins is tightly coupled with their ability to perform conformational motion [1]. To gain insights into protein function therefore requires the ability to infer the motion abilities inherently encoded in the protein’s structure. These protein motions vary widely in their temporal and spatial scales [2], making it difficult—if not impossible—to observe them directly with experimental methods. These methods can only provide structural snapshots or they can only access certain temporal and spatial resolutions [3–5].

Computational approaches to study protein motions aim to close this gap. They range from most accurate molecular dynamics (MD) simulations to highly simplified elastic network models (ENMs).

Molecular dynamics approaches—on one end of the spectrum—simulate atomistic motions based on precise physical force fields [6–9]. This results in what is believed to be a highly accurate understanding of protein motion. However, due to the computational requirements, only brief glimpses of protein motion can be obtained. In spite of increasing computational power, advances in parallelization [10–12], and special-purpose supercomputers [13–15], the practical usability of MD remains limited [11].

On the other end of the spectrum, efficient computational approaches make drastic simplifications to the underlying physics—but at the same time maintain a surprising biological accuracy. They exploit the fact that much information about protein motion seems to be captured in the protein’s contact topology, a simplified representation of the structural connectivity. These coarse-graining approaches, including the elastic network models (ENMs) [16–20], deliberately decrease the resolution of the underlying model to gain computational power, yet predict intrinsic protein motions of biological relevance [21–25].

Elastic network models, which will be the focus of this paper, are one form of simplified model that has been very successful. They represent a protein as a network of masses connected by springs. Each mass corresponds to a residue of the protein. Two masses are connected by a virtual spring if the respective residues are within a certain distance in the protein structure (we will also say that the residues are in contact).

There is a cost associated with the reduction in model complexity realized by ENMs. The simplicity prevents them from capturing functional transitions if they are localized or uncorrelated (low degree of collectivity) [21, 26–29]. Making matters worse, it is difficult to know a priori whether ENMs can model a protein’s motion accurately [28]. As a result, ENMs currently are not only limited to a particular type of protein motion, it is also difficult to know if a given protein exhibits that motion type. These factors limit the practical relevance of ENMs.

We propose a novel elastic network model that aims to improve the general applicability of ENMs by leveraging information to maintain the network’s connectivity. Our approach is based on the insight that ENMs capture function-related transitions only if the initial network topology (the springs) is maintained during the protein’s motion. Highly collective conformational changes naturally fulfill this requirement. Localized functional transitions, on the other hand, often lead to substantial changes in the contact topology and therefore in the corresponding network topology. We show that removing springs from the ENM for contacts that break during the motion enables ENMs to capture local and uncorrelated motions. Of course, to employ ENMs in situations when only a single conformation of the protein is known, we must also be able to *predict* these breaking contacts from that single conformation.

The core contribution of our approach is the ability to predict the dynamic behavior of contacts (whether they break or are maintained). To do so, we leverage information from the protein’s structure. This information is captured in the physicochemical characteristics of local parts of the protein structure. While these parts largely maintain their structural shape when the protein moves, they move with respect to each other controlled by the strength of their physicochemical interactions. Consequently, the mobility and deformability of these parts also affect their underlying contact topology, causing some contacts to break during a functional transition. To predict these breaking contacts, we use a machine-learning based classifier trained on a graph-based representation of their structural context [30].

Based on the predicted contact changes, we build a novel elastic network model, called *lmc*ENM, which only consists of learned maintained contacts. These contacts form the connectivity of the ENM, after the predicted breaking contacts have been removed. The adjusted contact topology of *lmc*ENM more likely remains valid when the protein moves and thus helps capture localized conformational changes. Although *lmc*ENM encodes additional information about the dynamic behavior of contacts, it still preserves the simplicity of the original ENM approach.

To evaluate *lmc*ENM, we apply it to a set of 90 proteins, covering functional transitions with different degree of collectivity. We show that *lmc*ENM accurately captures conformational changes that are poorly explained by the widely used, classical ENM and three reference ENM variants. In particular, *lmc*ENM is most effective in capturing localized functional transitions coupled with the binding of a ligand. While the classical ENM largely underestimates these localized transitions, we demonstrate that the adjusted contact topology of *lmc*ENM makes them accessible. We present case studies of two biologically interesting proteins selected from our data set, the outer membrane transporter FecA and Arachidonate 15-Lipoxygenase. Finally, we analyze which features contribute the most to correctly differentiate breaking from maintained contacts.

## Background: Elastic network models

The standard elastic network model (ENM) represents a protein as a network of point masses, each representing a residue. In this model, two residues are connected by a virtual spring, if their C_*α*_ atoms are within a predefined distance. Harmonic analysis of the resulting mechanical system then reveals the normal modes of the resulting mechanical system [25, 31]. The most dominant, low-frequency modes are commonly associated with the protein’s motion relevant for its function [32].

Elastic network models (ENMs) derive information about protein motion based on two main assumptions: First, the intrinsic motions of a protein can be approximated by a simplified, harmonic potential [16]. Second, the coarse-grained structure of a protein largely encodes these motions [17–20].

Due to the harmonic approximation made by ENMs, the accuracy of motion predictions deteriorates with distance from the initial conformation. Nevertheless, often a few low-frequency modes suffice to accurately explain functional transitions of proteins that are large-scale and highly collective [21–24]. This ability to narrow down the relevant deformation space (spanned by the essential low-frequency modes) makes NMA-based approaches particularly suited to guide conformational exploration [33, 34], docking simulations [27, 35, 36], or refinement of experimentally resolved structures [37, 38].

ENMs often fail to capture localized or uncorrelated motions [21, 26–29, 39, 40]. In these cases, extraneous constraints, introduced by the simple construction of the model, stiffen the network, preventing the ENM from reflecting localized protein motion. To overcome this limitation, as we will see in this paper, it is necessary to identify and remove these extraneous constraints from the network.

Refining elastic network models by exploiting additional information has a long tradition given their coarse-grained nature. However, one has to carefully balance how much and which additional information is actually relevant as computational cost increase with model complexity. We now briefly review related approaches that adjust network connectivity and/or stiffness, or interaction potential of ENMs. Based on the type of additional information we broadly categorize them into three groups: Methods that exploit (i) additional physcicochemical information, (ii) information about the protein’s structure, or (iii) information about the protein’s motion.

### Exploiting physicochemical knowledge

ENMs rely on the fact that physical forces gradually decrease with distance, i.e. residues close in space are more likely to move together than more distant ones. Basic ENMs use an arbitrary fixed distance cut-off and constant spring stiffness, possibly oversimplifying matters. Alternative approaches connect all residues in the network and select spring stiffness as function of residue distance [18, 41–44]. Apart from potentially over-constraining the network, a generic function for spring stiffness seems to be difficult to define [45].

Other approaches additionally consider the chemical type of the interaction. They vary spring stiffness between covalently bonded and non-bonded residue pairs [41, 46]. Jeong et al. [47] propose a chemical bond-cutoff ENM, where each CA-atom is connected to its four closest sequential neighbors and spring stiffness is varied with sequence distance. This implicitly guarantees network stability even for lower cutoffs that are usually not accessible for distance-cutoff based ENMs. Due to the sparser network they need to explicitly model chemical interactions, such as disulfide bridges, hydrogen bonds, or van-der-Waals forces. Recently, a mass-weighted variant has been proposed [48], which was further extended by symmetry constraints to better capture the packed state of protein crystals when their structure is determined experimentally [49]. These models are particularly accurate in terms of B-factor prediction. However, B-factors themselves provide a questionable source of information about protein motion due to the influence of crystal packing effects or errors introduced by molecular refinement [50].

### Exploiting structural knowledge

Some approaches tailor the connectivity and/or potential of the ENM to knowledge of the protein’s structure. The simplest way to achieve this is to consider interactions between more than two residues with a more complex potential [51–53], or additionally incorporate side-chain connectivity and chemical type [54]. It is also possible to consider additional backbone or side-chain atoms [55, 56], secondary structure [57], or information obtained from rigidity analysis [24, 58]. While the former trade physical accuracy for computational cost, the latter may introduce errors due to the additional coarse-graining.

If aspects of the structure are known to remain constant during the protein’s motion, it is possible to refine ENMs by adding additional constraints to maintain the overall structure, for example in the case of membrane proteins or larger protein complexes. Dony et al. [59] augment ENMs by adding springs between buried residues as well as between hydrogen-bonded residues.

### Exploiting knowledge about motion

The aforementioned approaches obtain the network topology of the ENM from a single, static protein conformation. Hence, there is no guarantee that the initial contact topology derived from this conformation remains valid when the protein moves. In some cases, however, we posses information about two or more conformations along the motion trajectory and use this information to improve the ENM.

One type of refinement is based on molecular dynamics (MD) simulations. Based on a single MD simulation, Hinsen et al [41] optimized a distance-dependent function to adjust spring stiffness. Orellana et al. [29] optimized connectivity and stiffness of the ENM based on short MD trajectories. They propose a three-staged hybrid potential with strongly connected sequential neighbors, distance-weighted springs for residues close in space, and a protein-size dependent cutoff to ignore irrelevant, remote interactions (see c. Additional ENM variants for evaluation in Methods for details). Their approach outperforms simpler ENM variants, but it remains questionable whether MD trajectories in the nano-second regime are able to cover the full space of motions accessible to proteins [60]. Globisch et al. [40] refine ENMs of protein complexes by analyzing short MD trajectories of their subunits. They reduce the network to bonds largely maintained throughout the simulations. The computational costs of the required MD simulations and the ability to only generate partial trajectories of the protein’s motion limit the applicability of this approach.

Another source of information are ensembles obtained by Nuclear Magnetic Resonance (NMR) or X-ray. For instance, Lezon et al. [45] derive optimal stiffness constants for secondary structure type and sequence distance between interacting residues using entropy maximation of NMR ensembles. Despite the good agreement between normal modes and PCA-modes from X-ray and NMR ensembles [61], the structural diversity of the latter may be biased towards missing experimental data [50].

When two conformations of a protein are known (e.g. open and closed conformation), the structural differences between these conformation allow to infer aspects of the intermediate motion. Song et al. [28, 62] use this information to tailor ENMs to the observed collective motions by varying the spring stiffness within (stronger) and between (weaker) domains. The resulting ENMs are more accurate, but they can only be obtained when two different conformations are available.

### Relation to our work

The aforementioned approaches indicate that a broad range of physicochemical, structural, and topological characteristics holds additional information about protein motion. However, the most important aspects of function-related movements may not necessarily be encoded in singular characteristics/properties, but rather result from their interplay as the work of Jamroz et al. [63] suggests.

Now the main question seems to be: how can we identify the combination of relevant characteristics to refine ENMs most effectively? We propose to learn these combinations from a large set of possible characteristics. In particular, we consider features that capture the influence of local and global structural topology on protein motion. Furthermore, we deliberately refine the network connectivity of ENMs without adjusting stiffness or interaction potential. This allows us to preserve the simplicity and computational efficiency of ENMs, while improving their general applicability. Still, the approach we present below can be used in conjunction with most of the previously mentioned methods of adjusting ENMs.

## Methods

We now present our approach to refine ENMs by leveraging additional information from physicochemical, structural, and topological properties of the protein structure. This information will ultimately be used to predict changes in the contact network of ENMs.

### A. Definition of contact topology

Two residues *i* and *j* are in contact if their distance is less than a pre-determined cutoff *r*_*c*_. The binary contact matrix *C* captures this network in its elements *C*_*ij*_:

$$\begin{array}{c} C_{ij}=\left\{ \begin{array}{cc} 1, & \text{if}\;d_{ij}\leq r_{c}\\ 0, & \text{otherwise} \end{array}\right. \end{array}$$ (1)

where *d*_*ij*_ denotes the Euclidean distance between the Euclidean coordinates of the C_*α*_ atoms representing residues *i* and *j*. The cutoff distance *r*_*c*_ depends on the type of ENM used and is often tailored to a protein and problem.

### B. Definition of contact changes and contact types

The contact topology is obtained from a single conformation and therefore encodes *static* structural connectivity. To identify function-related changes in connectivity, we compare the contact matrices for the start (S) and end (E) conformations of a functional transition. Based on the observed contact changes, we define a transition matrix *T*, whose elements, *T*_*ij*_, encode three different types of contact transitions: *maintained*, *breaking*, and *forming* contacts, defined as follows:

$$\begin{array}{c} T_{ij}=\left\{ \begin{array}{cc} \text{maintained}\;\text{contact}, & \text{if}\;C_{ij}^{S}=1\;\text{and}\;e_{ij}≜\left|\frac{\Delta d_{ij}}{d_{ij}^{S}}\right|\;\leq \;e_{c}\\ \text{breaking}\;\text{contact}, & \text{if}\;C_{ij}^{S}=1\;\text{and}\;e_{ij}≜\left|\frac{\Delta d_{ij}}{d_{ij}^{S}}\right|\;>\;e_{c}\\ \text{forming}\;\text{contact}, & \text{if}\;C_{ij}^{S}=0\;\text{and}\;C_{ij}^{E}=1\\ \text{no}\;\text{contact}, & \text{otherwise} \end{array}\right. \end{array}$$ (2)

where $C_{ij}^{S}$ ($C_{ij}^{E}$) refers to the entry for residues *i* and *j* in the contact matrix of the start and end conformation, respectively; *e*_*ij*_ denotes the distance change between residues *i* and *j* relative to their initial distance in the start conformation, where $\Delta d_{ij}≜d_{ij}^{S}-d_{ij}^{E}$. Intuitively, *e*_*ij*_ can be interpreted as strain measuring how much the distance between two particles in a body elongates (“stretch”) or shortens (“compression”) relative to their original distance. We limit the distance change by an upper bound *e*_*c*_ to distinguish breaking from maintained contacts.

### C. Prediction of contact changes as binary classification problem

#### a. Relevant types of contact changes

We consider two strategies to adjust the initial contact topology of ENMs: (i) removing breaking contacts, and (ii) removing breaking contacts and adding forming contacts. As will be discussed in detail in Results and Discussion, adding forming contacts worsens ENM performance. We therefore do not consider them and can now formulate the prediction of contact changes as a classification problem with two classes: breaking and maintained contacts.

#### b. Contact graph and secondary structure graph to generate features

To solve this binary classification problem, we will define a set of features. To be effective, these features should capture as much relevant structural information about the contact as possible.

To encode the local contact environment, we define the *immediate neighborhood graph* (IN_ij_) of a contact (for details see [30]). The graph consists of residues (nodes) and edges (between residues in contact). The nodes consist of the neighborhood of residues i and j. This includes residues i and j, the neighboring residues in sequence (positions i,j ±1), as well as all residues in contact with them. For *α*-helices, the neighborhood also includes residues i-4 and i+4 (similarly for j), which are the two closest neighbors one helix turn away.

In the neighborhood graph, nodes and edges are labeled. Node labels carry characteristics of individual residues, whereas edge labels characterize individual contacts. A detailed description of the labels can be found in the supplementary material (Tables B-C in S1 File). The labels are referred to as features and will be used for machine learning.

To characterize the embedding of a contact within the global structural topology of a protein, we also define the *secondary structure element (SSEs) graph*. The nodes correspond to secondary structure elements, i.e. *α*-helices, *β*-strands, or loops with a minimum length of three residues. Two nodes are connected by an edge if the corresponding SSEs are in contact, i.e. they share at least one residue-residue contact. Node labels (also called features) capture the characteristics of individual SSEs, whereas edge labels characterize the interface between two SSEs in contact. Based on the SSE-graph we distinguish between *intra*-SSE and *inter-SSE* contacts.

#### c. Overview of used features

We use a set of 75 features to characterize the properties of the local contact environment and its embedding into the overall structural topology. We concatenate these features into a feature vector that is then used to train and test our classifier. Continuous features are encoded as single, real-valued inputs, whereas categorical features are specified as a set of binary values. In total, the feature vector is 170-dimensional.

In addition to novel features specifically tailored to our problem, we add or adapt some features used in our previous work [30]. The features are grouped into seven categories (see Table 1): pairwise, graph topology, graph spectrum, single node, node label statistics, edge label statistics, and whole protein features. The supplementary material contains a detailed description of the individual features in each category and reports, which of the features are re-used or extended as well as external software used for their generation (Tables C-I and Text B in S1 File). We now introduce each feature category with some examples.

**Table 1:** Overview of used features.

Group	Feature examples	Number of inputs
Pairwise	Secondary structure element (SSE) type^a^, sequence separation between SSEs, distance between SSE centroids, symmetry coverage of SSE(s), intra-SSE contact and intra-SSE topology descriptors, inter-SSE contact and inter-SSE interface descriptors, contact residues part of terminal SSEs, hydrogen bonding^b^, side-chain contact, contact with pocket and number of atom contacts with pocket, pocket descriptors (polarity, hydrophobicity, volume, drug score), contained in symmetric segments, distance to symmetry plane, 4-bin contact depth and residue depth difference classes, mutual information^a^	63
Graph topology^a^	Number of nodes, number of edges, average degree centrality, average closeness centrality, average betweenness centrality, graph radius, graph diameter, average eccentricity, number of end points, average clustering coefficient	10
Graph spectrum^a^	Largest two eigenvalues, number of different eigenvalues, sum of eigenvalues, energy of adjacency matrix	5
Single node^a^	Degree, closeness centrality, betweenness centrality, sequence separation from N/C-terminus, sequence conservation and sequence neighborhood conservation for *i* and *j*	12
Node label statistics	Chemical type of residues^a^, secondary structure descriptors^a^, solvent accessibility^a^, hydrogen bonding^b^, average free solvation energy^a^, 4-bin solvation energy distribution^a^, entropy of labels, neighborhood impurity degree^b^, average distance from centroid^a^, symmetry coverage, average degree of symmetry, average residue depth, 5-bin distribution of residue depth, average lower/upper half-sphere exposure, sequence conservation^a^, sequence neighborhood conservation^a^	57
Edge label statistics	Link impurity^b^, 5-bin mutual information distribution^a^, cumulative mutual information^a^	13
Whole protein	Secondary structure composition^a^, 5-bin connectivity class based on number of contacts, symmetry coverage	10
		170

^*a*^ Added from Schneider et al. [30].

^*b*^ Adapted from Schneider et al. [30].

Table lists the features used by our classifier to predict function-related contact changes in the contact topology of proteins. Added or adapted features from Schneider et al. [30] are marked. If all features in one category are added from Schneider et al. the category is marked instead of the individual features. The supplementary material describes individual features and their implementation in detail.

Pairwise features encode properties of an individual contact. As contacts seldom change their distance in isolation, many of the pairwise features are defined on their associated secondary structure element(s) (SSEs). The features capture for instance SSE types, sequential and three-dimensional distance between the SSEs, hydrogen bonding between SSEs, closeness to empty pockets, or closeness to binding site.

To capture topological characteristics of the local contact environment we re-use the graph-topology, graph spectrum, and single node features from Schneider et al. [30]. For instance, contacts embedded into a highly constrained neighborhood are less likely to change than contacts in sparsely connected local contact networks. The average number of neighbors of each node in the local contact environment can be characterized by the average degree centrality.

Node and edge label statistics encode properties of the contact’s neighborhood not captured by its topology. For example, local contact networks with high symmetry coverage, i.e. most residues belong to a symmetric segment of the protein, are likely to maintain their connectivity even when the protein moves. This can be measured by the normalized number of symmetric residues.

We further collect properties of the whole protein, such as the connectivity class based on the total number of contacts, and the distribution of secondary structure types. These features now serve as input to train and test our classifier.

#### d. SVM training

We train a support vector machine (SVM) to differentiate breaking from maintained contacts, given the features described above. This classifier builds upon an in-house contact prediction framework [30]. We face a highly imbalanced learning problem because proteins seem to have rather few breaking contacts (on average 4.5% of all contacts of a protein in our data set). A common approach to tackle such problems is random undersampling of the majority class [64]. For each protein in our data set, we take all observed breaking contacts as positive samples, while randomly picking three times as many maintained contacts as negatives. In addition, we adjust the penalty term *c* of the SVM by a class-dependent weighting factor, which is inversely proportional to the frequency of the class, as implemented in [65]. This increases the importance of correctly classifying positive samples.

The SVM classifier yields a probability score for each sample to belong to the positive class, i.e. to be a breaking contact. This probability is estimated with Platt’s scaling method [66] based on the binary classification scores of the SVM [65].

To train the SVM we use the radial-basis-function (RBF) kernel and determine the hyperparameters, cost *c* and kernel parameter *γ*, in a leave-one-out cross-validation on our data set (see E. Protein Data Set). The hyperparameters are tuned by optimizing the precision (Prec = TP/(TP+FP)) of the *L*/5 contacts with highest SVM score, where *L* refers to the length of the protein. Predicted breaking contacts that have also been observed are true positives (TP), whereas predicted ones not observed to break are false positive predictions (FP). The SVM with cost *c* = 100 and kernel parameter *γ* = 0.00001 reaches the highest average precision in our setting.

### D. Constructing *lmc*ENM—The elastic network of learned maintained contacts

To build *lmc*ENM for a given protein, we follow three steps: First, we feed the classifier with the contact network of the unbound conformation to predict which contacts have highest probability to break. Second, we select a subset of top scoring predicted contacts. And third, we remove these selected contacts from the initial network, yielding the network of learned maintained contacts, *lmc*ENM. In the following, we describe each of these steps in detail.

The SVM classifier scores all contacts in the initial network that connect residues at least four sequence positions apart. The reason is that removing shorter range breaking contacts from *mc*ENM and *lmc*ENM yielded unstable networks in several cases (see A. in Results). In addition, the improvement in accuracy caused by additionally removing these shorter range contacts was negligible for the stable networks. The classifier outputs the contacts ranked by decreasing confidence score, which indicates their likeliness to break.

To construct *lmc*ENM, we now seek a function that tells us how many of the top scoring predicted contacts we should remove from the initial contact topology. We expect that the amount of breaking contacts depends on the collectivity of the function-related movement. Local, uncorrelated motions likely require more initial contacts to break than large-scale, collective motions. However, in most cases the nature of the functional transition is unknown a priori and furthermore depends on various properties of the protein. This makes it difficult to find such a function. Therefore, we tested three simple strategies to select the subset of predicted breaking contacts to be removed:

**Constant cutoff** This strategy removes the top *n* predicted breaking contacts. It is based on the rationale that the amount of breaking contacts is limited in number and variance among different proteins. Given our assumption that breaking contacts are most relevant to capture localized, functional transitions, we would expect that they concentrate on particular regions of the protein. The spatial extent of these regions should be rather small and not necessarily depend on the protein’s size.
**Relative cutoff** This strategy removes the top *n* percent of predicted breaking contacts. Opposite to the previous strategy we now assume that the amount of breaking contacts is affected by the total number of contacts of the protein.
**Score-dependent cutoff** This strategy removes all predicted breaking contacts with probability larger than a predefined cutoff score. Here, we assume that prediction accuracy of the classifier is comparable among different proteins.

We evaluated each strategy on a predefined set of cutoff values to empirically determine the most effective strategy and associated cutoff value in our setting (see Results and Discussion).

Finally, we adjust the initial contact topology of the considered protein by removing the selected predicted breaking contacts. The outcome is our novel elastic network of learned maintained contacts, called *lmc*ENM.

### E. Protein data set

To train and test our classifier as well as to evaluate the performance of *lmc*ENM, we chose a set of proteins with known motion type. The *Protein Structural Change DataBase (PSCDB)* [67] provides motion classified protein pairs, each representing the functional transition of one protein family in the SCOP (Structural Classification of Proteins) database. A pair consists of two conformations, marking start and end of the functional transition, where only the latter is bound to a ligand, the former is unbound. The PSCDB classifies each of these functional transitions into six motion types (see below). In particular, it distinguishes highly collective, domain motions from localized, uncorrelated transitions. This allows us to explicitly assess the ability of our approach to explain localized, functional transitions that are elusive for classical ENMs.

We applied several filters to extract a meaningful and consistent data set from the PSCDB. We exluded proteins: (a) without significant motion (root mean squared distance (RMSD) ≤ 1.0*Å*), (b) with less than 70 residues alignment length, (c) with resolution higher than 2.5*Å* (d) including chain breaks (defined as more than 4.2*Å* Euclidean distance between two consecutive *C*_*α*_ atoms along the sequence [68]), (e) including a peptide with more than six non-hydrogen atoms in the unbound conformation [69], and (f) with largely extended or disordered structures. Furthermore, we limited ourselves to single-chain proteins to enable faster development and testing. Filters (a) and (d) exclude proteins encoding little to no information about protein motions, whereas (b), (c), (e), and (f) exclude proteins for which this information is distorted due to low structural quality, highly specialized structural topology, or interaction with other chains.

Our final data set of 90 protein pairs (LMC_all, see Dataset A in S2 File) is distributed across the following motion classes: coupled domain motion (short: CDM, 21 protein pairs), independent domain motion (IDM, 14), coupled local motion (CLM, 27), independent local motion (ILM, 18), buried ligand motion (BLM, 4), and other types of motion (OTM, 6). Both domain and local motions can be associated with ligand binding (coupled) or without (independent). Proteins that are bound to a ligand in the end conformation, but lack considerable movement between start and end, are categorized as buried ligand motions. Although, these proteins move to bind the ligand, the structural differences between the two conformations are small because the ligand-free conformation seems to imitate the shape of the ligand by occluded water molecules [67]. All remaining proteins fall into the category other types of motions.

The length of proteins in our data set ranges from 70 to 712 amino acids. The RMSDs (root mean squared distances) between the unbound and bound conformation lie between 1.1*Å* and 9.6*Å*. We use leave-one-out cross-validation (LOOCV) for training and tuning of the hyper parameters of the SVM (see d. SVM Training). This allows to maximize the amount of data used to train individual classifiers for each protein. Based on the rank-ordered list of predicted breaking contacts we then adjust the contact topology of the initial conformation to construct *lmc*ENM.

### Anisotropic network model theory

Our novel elastic network model, *lmc*ENM, is based on the widely used anisotropic network model (ANM) [17, 20, 21]. The ANM captures interactions between spatially close residues by a Hookean potential. The generalized form of the entire network potential of the ANM is given by

$$\begin{array}{c} V_{\text{ANM}}=\sum_{i,j/i\neq j}^{N}\frac{k_{ij}}{2}{\left(d_{ij}-d_{ij}^{0}\right)}^{2} \end{array}$$ (3)

where *d*_*ij*_ and $d_{ij}^{0}$ denote the instantaneous and equilibrium distance of residues *i* and *j* measured between their *C*_*α*_ atoms; *N* is the number of residues of the protein. *k*_*ij*_ is the force constant defined as

$$\begin{array}{c} k_{ij}=\gamma \cdot C_{ij} \end{array}$$ (4)

where *γ* is a uniform stiffness constant and *C*_*ij*_ ∈ {0, 1} refers to the entry of residues *i* and *j* in the contact topology matrix of the initial conformation as defined in Eq (1) (see A. Definition of Contact Topology).

The intrinsic deformability of an ENM is calculated by normal mode analysis (NMA). NMA solves the eigenvalue problem of the Hessian matrix defined by the second derivatives of the network potential *V*_ANM_ with respect to the mass-weighted positions of the network nodes. A detailed derivation of the Hessian matrix can be found in the original publications [17, 20]. The resulting 3*N* − 6 eigenvectors (normal modes) span the complete deformation space of the ENM. The excluded six eigenvectors with zero-frequency correspond to the external rigid body motions of the entire network. The normal modes are ranked according to their eigenvalues that specify the energetic cost of a deformation along the mode direction. The highest ranked low-frequency modes represent large-scale collective deformations of the system. As they are energetically favorable, they are easy to access and hence dominate the intrinsic movements of the ENM. High-frequency modes correspond to local movements requiring high-energy.

### F. ENM parameterizations

#### a. Baseline ENM

The cutoff distance of ANMs is often adjusted problem-wise and ranges between 8-15A [20, 23, 50, 70–72]. ANMs at smaller cutoffs may become unstable due to the sparser network. Hence, cutoff values of 12Å and larger are typically chosen [23, 47, 50], which render the ANM less suitable to predict localized functional transitions. We therefore tried to lower the cutoff distance for our data set as much as possible without making the network unstable.

In our study, we evaluated the cumulative mode overlaps of the ANM at cutoff values ranging from 8 to 18Å. At cutoffs lower than 11Å, some networks became unstable yielding more than the trivial six zero eigenvalues: 20 cases for ANM8, 9 cases for ANM9, and 3 cases for ANM10 from our full set of 90 proteins. We therefore tried to stabilize the ANMs at cutoffs lower than 11A.

To be stable ENMs must fulfill two requirements [47]: (i) each node must be connected to at least four other nodes, (ii) the network must have at least 3*N* − 6 edges, where *N* refers to the number of nodes. As first step towards stabilizing the ANM network at lower cutoffs we therefore enforce that each C_*α*_ atom is constrained by at least four neighbors (node degree > = 4). Under-constrained C_*α*_ atom get connected to their closest–not yet connected–neighbors along the sequence irrespective of their distance.

Table J in S1 File shows the performance of this network, called ANM_*minDeg*4_, at different cutoffs. The 4-neighbor-connectedness criterion largely reduces the number of networks yielding more than six zero eigenvalues, yet some networks remain unstable at cutoffs 8 and 9Å. However, ANM10_*minDeg*4_ now fulfills the criterion of six zero eigenvalues for all proteins and yields largest agreement between predicted and actual motion directions, measured by the cumulative mode overlap (median and mean) of the first ten low-frequency modes. Hence, we chose it as baseline for our approach. This is in line with Kondrashov et al. [70] who found that a distance-cutoff of 10Å yields largest agreement in overlap of motion directions, but less accurate prediction of motion magnitudes. In turn, the best match of motion amplitudes (fluctuation profiles) requires cutoffs larger than 15Å, thereby reducing the overlap in motion directions by increasing structural stiffness and collectivity of motion. This counteracts our goal to accurately model localized functional transitions with low degree of collectivity.

In the following, we refer to our baseline ANM10_*minDeg*4_ simply as ENM.

#### b. *mc*ENM and *lmc*ENM

*mc*ENM, the network of observed maintained contacts (theoretical upper bound), and *lmc*ENM (our method) base on the above chosen baseline ANM10_*minDeg*4_ at distance cutoff $r_{c}=10Å$ and uniform springs. The parametrization of both networks considering the amount of removed (predicted) breaking contacts is reported in a. Experimental Design and Parametrization of the results section.

#### c. Additional ENM variants for evaluation

Apart from baseline and theoretical upper bound, we benchmark our approach against three other ENM variants: (i) a cutoff-free model with distance-dependent force constants, HCA [41], (ii) a model optimizing force constants based on structural properties (OFC-ENM) [45], and (iii) a hybrid model combining a bond-cutoff strategy for close sequential neighbors and distance-dependent force constants for remote interactions, edENM [29]. All variants use the general form of network potential, *V*_*ANM*_, as defined in Eq (3) Anisotropic Network Model Theory.

The HCA method [41] defines the spring stiffness between all residue pairs in the network using a fast decaying distance-dependent function:

$$\begin{array}{c} k_{ij}=\left\{ \begin{array}{cc} a\cdot d_{ij}-b, & \text{if}\;d_{ij}<r_{c}\\ c\cdot {\left(d_{ij}\right)}^{-d}, & \text{otherwise} \end{array}\right. \end{array}$$ (5)

where *d*_*ij*_ is the Cartesian distance between residues *i* and *j*. We used the parametrization of the original publication ($a=205.5\;\text{kcal}\;{\text{mol}}^{-1}{Å}^{-3}$, $b=571.2\;\text{kcal}\;{\text{mol}}^{-1}{Å}^{-2}$, $c=3.059*10^{5}\;\text{kcal}\;{\text{mol}}^{-1}{Å}^{4}$, *d* = 6, and $r_{c}=4.0Å$).

OFC-ENM [45] scales spring stiffness based on secondary structure type and sequential distance between interacting residues. The optimal stiffness constants are obtained by analyzing NMR-ensembles using entropy maximization. We use OFC-ENM with the distance cutoff 10Å and the default parameter set as implemented in ProDy [73].

edENM [29] distinguishes three types of interactions. Residues close in sequence (up to three sequence positions apart) build a fully connected network where spring stiffness depends on sequence distance. Interactions between residues within a protein-size-dependent cutoff, *r*_*c*_, are modeled with distance-dependent springs. Irrelevant, remote interactions above the cutoff are excluded from the network. This leads to the following definition of spring stiffness between residues *i* and *j*:

$$\begin{array}{c} k_{ij}=\left\{ \begin{array}{cc} a/{\left(s_{ij}\right)}^{b}, & \text{if}\;s_{ij}\leq 3\\ {\left(c/d_{ij}\right)}^{d}, & \text{if}\;s_{ij}>3\;\text{and}\;d_{ij}\leq r_{c}\\ 0, & \text{otherwise} \end{array}\right. \end{array}$$ (6)

where *d*_*ij*_ (*s*_*ij*_) is the Cartesian (sequential) distance between residues *i* and *j*, respectively; *r*_*c*_ = 2.9 ∗ ln(*N*) − 2.9 is a size-dependent distance cutoff with *N* being the number of residues of the protein. We used the default parametrization of the original publication, which was optimized based on MD simulations ($a=60\;\text{kcal}\;{\text{mol}}^{-1}{Å}^{-2}$ and *b* = 2; $c=6\;\text{kcal}\;{\text{mol}}^{-1}{Å}^{-2}$ and *d* = 6).

For all tested ENM variants we only analyze ENMs based on unbound (start) protein conformations, because they generally capture more of the functional transition than the more compact bound (end) conformation [21, 28, 54].

### G. ENM evaluation measures

Coarse-grained ENMs often guide more detailed exploration of protein motions (see Background: Elastic Network Models). The value of this guidance largely depends on two factors: First, how well can the guidance be trusted, i.e. how accurate is the prediction of the essential deformation space that has to be searched? Second, how much can it reduce computational cost by narrowing down this search space?

#### a. Assessing biological accuracy

A common measure to evaluate the accuracy of ENMs is the *mode overlap* *O*_*j*_ [21, 74]. It specifies the amount of conformational change captured by a single mode *j* based on the angle between conformational displacement vector and mode direction vector *M*_*j*_, as defined in:

$$\begin{array}{c} O_{j}=\frac{|\sum^{3N}M_{j}\Delta r_{i}|}{{\left[\sum^{3N}M_{j}^{2}\cdot \sum^{3N}\Delta r_{i}^{2}\right]}^{1/2}} \end{array}$$ (7)

where $\Delta r_{i}=\left(r_{i}^{S}-r_{i}^{E}\right)$ denotes the displacement vector from start ($r_{i}^{S}$) to end conformation ($r_{i}^{E}$) at residue *i*; *N* is the number of residues of the protein. The measure ranges between 0 and 1 (perfect match).

By summing up the individual mode overlaps of the first *k* low-frequency modes, we now can specify their *cumulative mode overlap* *CO*(*k*) [28]. It indicates how accurate the deformation space spanned by these modes captures the functional transition, given by:

$$\begin{array}{c} CO\left(k\right)={\left[\sum_{j=1}^{k}O_{j}^{2}\right]}^{1/2}. \end{array}$$ (8)

In principle, the number of low-frequency modes required to span the *essential* deformation space is unknown. This is due to its strong coupling to the collectivity of the functional transition (see Background: Elastic Network Models). However, usually less than ten modes suffice to accurately capture function-related movements that are highly collective. In the results section, we thus assess the cumulative mode overlap of the first ten low-frequency modes *CO*(10) unless stated otherwise. We use *CO*(10) as main measure for benchmarking the different ENM variants. To avoid over-fitting to a single measure we evaluate a set of other commonly used metrics described below.

The *Pearson correlation coefficient* is used to measure the similarity between predicted residue fluctuations and observed displacements, as well as between predicted fluctuations and experimental B-factors from the unbound conformation. Predicted fluctuations were scaled to observed displacements and B-factors, respectively. The correlation coeffient ranges between -1 (total negative correlation), 0 (no correlation) and +1 (total positive correlation).

The *fraction of variance* of a mode measures how much of the structural variance it explains. It is defined by the variance of mode *j* divided by the trace of the covariance matrix of the model. The *cumulative fraction of variance* *CFV*(*k*) sums up the individual contributions of the first *k* low-frequency modes.

The *degree of collectivity* *κ*_*i*_ [75] of a protein motion quantifies the number of involved residues. It is given by:

$$\begin{array}{c} \kappa_{j}=\frac{1}{N}\exp\left(-\sum_{i}^{N}u_{j,i}^{2}\log u_{j,i}^{2}\right) \end{array}$$ (9)

where *N* denotes the number of residues of the protein and $u_{j,i}^{2}$ is defined as $u_{j,i}^{2}=\alpha \frac{1}{m_{j}}\left(M_{j,X}^{2}+M_{j,Y}^{2}+M_{j,Z}^{2}\right)$ with *M*_*j*_ being the *j*-th mode vector and *m*_*j*_ its mass; *α* is a normalization factor to ensure that $\sum_{i}Nu_{j,i}^{2}=1$. The measure varies between 1/*N* (only one residue affected) and 1 (maximally collective).

#### b. Assessing dimensionality of deformation space

The dimensionality of the essential deformation space depends on the desired accuracy. Therefore, we assess the number of modes required to capture 70%, 80%, and 90% of the functional transition (measured in percent cumulative mode overlap). Lower dimensionality effectively reduces computational cost for subsequent exploration of this space.

In addition, we report the *maximum overlap* *MaxO*(*j*) among the first *j* modes, together with the rank of the corresponding mode (rank 0 refers to the first mode), its collectivity, and fraction of variance.

#### c. Comparing against essential dynamics of conformational ensembles

Conformational ensembles obtained from structural databases provide an additional source to characterize protein flexibility [76–78]. For a subset of proteins we obtained such an ensemble and analyzed its Essential Dynamics (ED) using Principal Component Analysis (PCA) as implemented in ProDy [73]. We use the following measures to analyze the similarity between ENM deformation space and principal components space.

The *Pearson correlation coefficient* *CC* is used to determine the similarity between the mean square fluctuations captured by ED and the squared fluctuations of the ENM. It varies between -1 (total negative correlation), 0 (no correlation) and +1 (total positive correlation).

The *root mean square inner product (RMSIP)* [79] measures the similarity of two vectorial spaces by the overlap of their *k*-dimensional subspaces:

$$\begin{array}{c} \text{RMSIP}\left(k\right)={\left[\frac{\sum_{i,j=1}^{k}{\left(U_{i}\cdot V_{j}\right)}^{2}}{k}\right]}^{1/2} \end{array}$$ (10)

where *U*_*i*_ and *V*_*j*_ are the eigenvectors/principal components of the compared covariance matrices; *k* is the dimensionality of the subset of low-frequency modes/principal components. Commonly, *k* is set to an arbitrary value of 10. RMSIP ranges between 0 and 1 (perfect match).

A related measure of vector space similarity is the *root weighted square inner product (RWSIP)* [80]. In contrast to the RMSIP it considers the relative contribution of each eigenvector (direction) weighted by its corresponding eigenvalue (magnitude). Further, it takes into account the full spaces to be compared instead of a small subspace. The RWSIP is given by:

$$\begin{array}{c} \text{RWSIP}={\left[\frac{\sum_{i,j=1}^{N}u_{i}v_{j}{\left(U_{i}\cdot V_{j}\right)}^{2}}{\sum_{i=1}^{N}u_{i}v_{i}}\right]}^{1/2} \end{array}$$ (11)

where *U*_*i*_ and *V*_*j*_ are the eigenvectors/principal components of the compared covariance matrices; *u*_*i*_ and *v*_*j*_ are the eigenvalues; *N* is the number of non-trivial eigenvectors in each mode set. ENM eigenvalues have been inverted to be proportional to the relative amplitudes captured by PCA eigenvalues. RWSIP ranges between 0 and 1 (perfect overlap).

## Results and discussion

We present two areas of results: The first part (A. ENMs and the Effect of Removing Observed Breaking Contacts) provides the biological grounding of our approach. By assuming perfect knowledge we examine whether ENMs are able to capture *localized*, functional transitions of proteins. The second part (B. Evaluation of *lmc*ENM–Elastic Network of Learned Maintained Contacts) investigates if we can transfer this knowledge into a novel elastic network model that alleviates this major shortcoming of ENMs.

### A. ENMs and the effect of removing observed breaking contacts

Our work is based on the assumption that localized, functional transitions often require substantial changes in the contact topology of a protein. Consequently, refining ENMs based on *observed* contact changes should improve the match between predicted and actual motions.

#### a. Experimental design and parametrization

To validate this assumption we analyzed for each protein in our dataset how contacts change between a pair of high-resolution conformations obtained by X-ray crystallography. This is in contrast to the growing work favoring MD simulations to optimize and benchmark ENMs (see recent reviews [72, 81] and citations therein). Clearly, two conformations capture only part of the structural variability of conformational ensembles. However, the chosen conformations represent the end points of a functional transition determined by largest structural difference among known conformations of a protein family [67]. This increases the chances that the associated function-related structural changes are not only relevant but also appear in their coarse-grained contact topology, which may be more difficult to identify in structural ensembles obtained by MD simulations or Nuclear Magnetic Resonance. Both have a limited view on actual structural variance due to inaccuracies in sampling or measurement and still have restrictions on the protein’s size. Further, the captured structural differences may be too small to effectively change the simplified contact topology. Also, energy barriers may prevent MD simulations from accessing certain conformational states. Such an effect is commonly associated with the induced-fit mechanism, where the presence of the binding partner triggers the required conformational change for successful binding [82].

Apart from maintained contacts, we observe contacts that break, and contacts that form (see Methods for details). To identify the transition that is relevant to capture localized movements, we considered two strategies to adjust the initial contact topology of the ENM: (i) removing breaking contacts, which yields the network of maintained contacts, *mc*ENM, and (ii) removing breaking and adding forming contacts, which results in the network of maintained and forming contacts, mfcENM. To distinguish breaking from maintained contacts we used an empirically defined extension threshold of 9% of the initial contact distance that maximizes the median accuracy improvement of *mc*ENM for our dataset (Table K in S1 File).

To maintain network stability the baseline ENM at distance-cutoff 10Å requires at least four neighbors in contact for each residue, which must not necessarily be the closest four along the sequence (see a. Baseline ENM in Methods for details). However, *mc*ENM and *lmc*ENM became instable in several cases when removing observed breaking contacts based on the above defined extension threshold. This was due to the removal of contacts with less than four amino acids sequence separation. Hence, we had to tighten this stability criterion for both, *mc*ENM and *lmc*ENM, to remove breaking contacts only if their residues are at least four sequence positions apart. The criterion for six zero eigenvalues is maintained after removal of breaking contacts in both, *mc*ENM and *lmc*ENM, for all proteins (Table F in S2 File).

#### b. Observed breaking contacts matter

Breaking contacts seem to be weaker than maintained ones because they loose contact during a functional transition. Modeling them as strong as other contacts (uniform springs) thus inhibits actually accessible movements. The simplest approach to release these artificial/erroneous constraints is to remove breaking contacts from the initial contact network.

Forming contacts, in contrast, establish towards the end of a conformational change due to the more compact fold of the bound conformation. Hence, they further constrain the initial contact network. Even if we incorporate them into the less constrained network of maintained contacts, they rather inhibit required movements than enable them. Therefore, we expect improvement in accuracy for *mc*ENM, but not for mfcENM.

**Fig 1:**
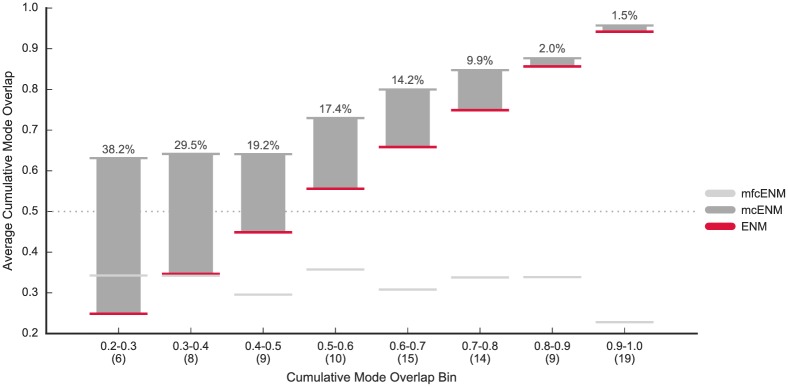
Accuracy of *mc*ENM and *mfc*ENM compared to ENM on our data set (90 proteins). Accuracy is measured by the cumulative mode overlap of the first ten low-frequency normal modes (*CO*(10)). Proteins are binned based on the cumulative mode overlap reached by ENM (#proteins per bin is given in brackets). The horizontal lines mark the average accuracy per bin (absolute improvement of *mc*ENM over ENM given by numbers above each bin). *mc*ENM consistently improves over ENM being particularly effective for proteins poorly captured by ENM (indicated by the gray dotted line). In contrast, mfcENM performs much worse than ENM.

In Fig 1, we compare the accuracy of *mc*ENM and mfcENM with the baseline ENM in terms of their cumulative mode overlap. Detailed results for every protein are given in Table C in S2 File. *mc*ENM consistently improves over ENM, whereas mfcENM drops far below the baseline in almost all cases. Therefore, mfcENM is excluded from the rest of the evaluation. *mc*ENM is particularly effective for proteins that are most difficult to capture with ENM (*CO*(10) ≪ 0.6). For proteins in these four leftmost bins, *mc*ENM gains between 7.0% up to 58.7% improvement in accuracy (Table C in S2 File). As expected *mc*ENM improves less in accuracy for proteins, whose functional transitions are well captured by ENM. Furthermore, *mc*ENM substantially increases the number of proteins reaching 60% coverage of the functional transition with only ten lowest-frequency normal modes (*mc*ENM: 92% of proteins, ENM: 63%, see Table C in S2 File).

Table 2 shows that the improvement of *mc*ENM over ENM is consistent over all evaluated metrics. For detailed results see Table C in S2 File. *mc*ENM more accurately captures the functional transition not only in terms of motion directions (overlap, structural variance), but also w.r.t. motion amplitudes (correlations between fluctuation profiles and temperature factors, where *mc*ENM is on par with ENM). Further, *mc*ENM reaches higher overlap and better agreement in structural variance for the best-overlapping mode, which is shifted towards the lower frequency spectrum of modes (rank). Hence, this mode becomes more dominant, which is desired for the most relevant mode. *mc*ENM also largely reduces the amount of modes required to explain a certain percentage of cumulative mode overlap (see next paragraph for details). Only when considering the degree of collectivity, i.e. how many residues are involved in the movement, *mc*ENM reaches lower values than ENM. We will investigate this further in the following paragraph.

**Table 2:** Evaluated similarity measures for ENM and *mc*ENM.

	ENM	*mc*ENM
	(median/mean)	(median/mean)
Cumul. Mode Overlap (10)	0.69/0.66	0.82/0.80
Cumul. Fraction of Variance (10)	0.35/0.38	0.57/0.59
CorrCoeff Fluctuations—Displacements (10)	0.52/0.50	0.81/0.78
CorrCoeff Temperature Factors—Betas (10)	0.40/0.40	0.41/0.40
Max Overlap	0.47/0.50	0.60/0.62
Rank (Max Overlap Mode)	1.00/11.08	0.00/1.93
Degree of Collectivity (Max Overlap Mode)	0.38/0.39	0.27/0.31
Fraction of Variance (Max Overlap Mode)	0.05/0.08	0.12/0.20
#Modes Cumul. Mode Overlap (70%)	11.00/34.51	3.00/6.92
#Modes Cumul. Mode Overlap (80%)	35.00/79.07	7.50/19.41
#Modes Cumul. Mode Overlap (90%)	164.50/200.60	39.00/75.56

For several measures we consider only the subset of the first ten low-frequency modes indicated by (10) after the measure’s name. Except for Rank and Collectivity of the best-overlapping mode higher values are better. A lower rank of the best overlapping mode with the observed displacement vector indicates that the most relevant motion captured by the elastic network is also more dominant. In terms of Degree of Collectivity, we find that lower values indicate that less collective, localized functional transitions are better captured (see next paragraph for more details).

Our results indicate that observed breaking contacts actually matter in contrast to forming ones. Their absence improves ENM accuracy, and is most effective in capturing otherwise poorly explained function-related movements.

#### c. *mc*ENM accurately captures localized functional transitions

Now we need to show that our strategy works in particular for proteins with localized, functional transitions. To validate this assumption we analyzed the performance of *mc*ENM w.r.t. the motion type of the proteins (see Methods for details on motion classification of our data set).

**Fig 2:**
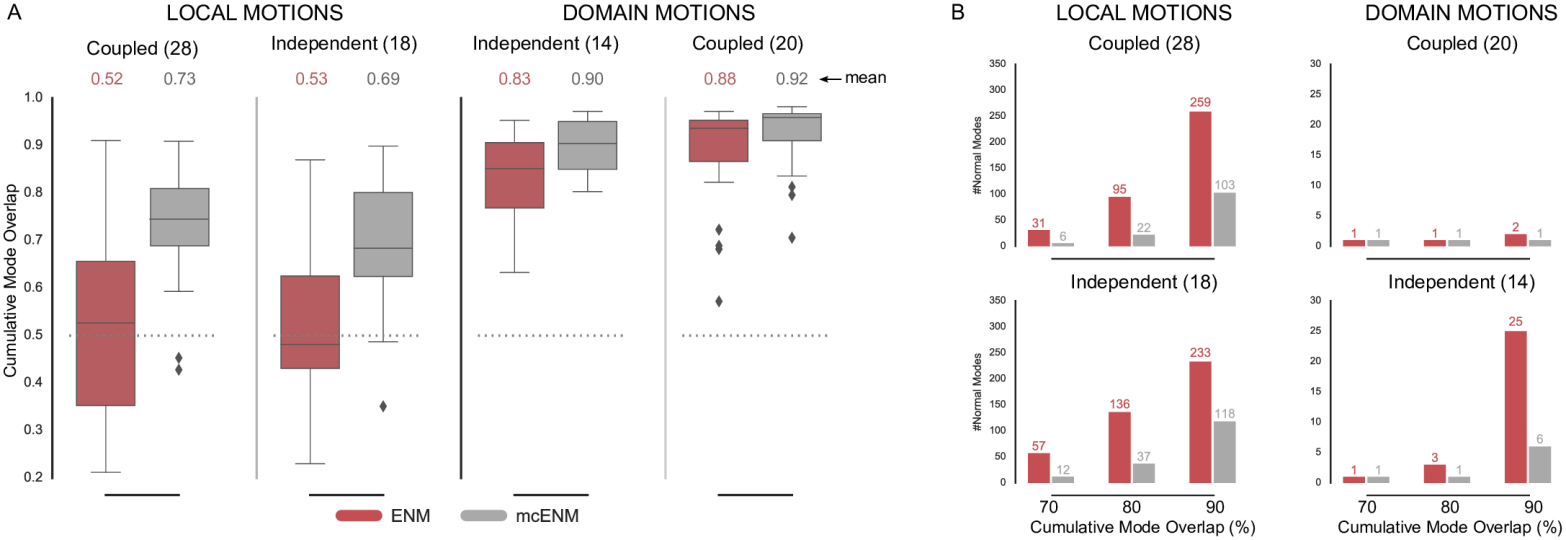
Accuracy of *mc*ENM compared to ENM measured by cumulative mode overlap (A) and dimensionality of deformation subspaces(B) of *mc*ENM on subset of local and domain motions (80 proteins). (A) The distribution of cumulative mode overlap is evaluated for the first ten low-frequency normal modes (*CO*(10)). *mc*ENM consistently improves over ENM in each motion category. *mc*ENM is particularly effective for proteins with localized functional transitions yielding an improvement between 15% and 21% for independent and coupled local motions. (B) The panels show the median number of normal modes (spanning the deformation subspace) required to explain between 70% and 90% of the functional transition (measured in cumulative mode overlap (%)). *mc*ENM consistently requires fewer modes to capture the same amount of conformational change as ENM.

Fig 2A shows the distribution of cumulative mode overlap of *mc*ENM and ENM for proteins classified as local vs. domain movers. Both categories are further subdivided into ligand-coupled or independent motions. *mc*ENM consistently improves over ENM for the shown motion types. However, proteins with localized functional transitions benefit by far the most. Here, *mc*ENM captures both coupled (independent) transitions on average 21% (15%) more accurate than ENM. For the domain motions already well captured by ENM, *mc*ENM still improves between 4% and 7% on average.

*mc*ENM substantially improves over ENM also in terms of other metrics, such as the structural variance captured by the lowest frequency modes and the similarity between predicted and observed fluctuation profiles (Fig A(A,B) in S1 File). Again, local movers benefit the most. Correlating predicted and experimentally observed temperature factors yields comparable performance of *mc*ENM and ENM (Fig A(C) in S1 File). To better capture experimental B-factors ENMs require larger distance cutoffs (>16 Å) thereby increasing structural stiffness and collectivity of motion [70]. This counteracts our goal to accurately model localized functional transitions with low degree of collectivity. Hence, this metric has little relevance in our context.

Our results show that *mc*ENM, in fact, is able to capture localized, functional transitions while largely outperforming the distance-cutoff based ENM. Apart from comparing the agreement between motion directions and magnitude, we also evaluate the complexity of the resulting essential deformation space.

#### d. *mc*ENM reduces dimensionality of essential deformation space

ENMs often guide more fine-grained exploration by narrowing down the search space (essential deformation space). The computational cost of searching this space increase with dimensionality (number of spanning modes). Hence, lower dimensional search spaces are desirable as long as they are accurate enough. As mentioned above the common strategy to consider between 10-20 lowest-frequency modes works well in capturing highly collective functional transitions, but fails for localized functional transitions with low degree of collectivity. Here, the relevant modes (usually less than 10) are often spread among higher frequencies [27]. Consequently, a much larger number of ENM modes would need to be considered to capture them, which in turn yields a higher dimensional search space. In the following, we analyze how the absence of breaking contacts affects the relationship between desired accuracy and number of required modes. As above, we focus on local and domain motions.

Fig 2B depicts the median number of modes required to achieve a cumulative overlap of 70%, 80%, and 90%. *mc*ENM needs much less modes to be as accurate as ENM, thereby substantially reducing the dimensionality of the associated deformation space. For instance, to capture 80% of ligand-coupled local motions *mc*ENM requires a median of 22 modes, whereas ENM needs 95. Being less constrained, *mc*ENM favors otherwise high-energetic modes that seem to be relevant to capture the function-related movement. Hence, these modes “shift” towards lower frequencies. Consequently, *mc*ENM reaches higher accuracy with fewer, but more relevant low-frequency modes because their individual contribution to the overlap is higher.

This mode shifting is further supported by the large decrease in rank of the best-overlapping mode of *mc*ENM compared to ENM as shown in Fig B in S1 File. *mc*ENM not only captures the direction of this mode much more accurate, but also increases its contribution to the structural variance to a large extent. Interestingly, the degree of collectivity of the best-overlapping mode for proteins with localized functional transitions is much smaller when being analyzed by *mc*ENM instead of ENM. Hence, the best-overlapping *mc*ENM-mode must be more relevant for the local transition given its higher overlap and larger variance. A similar “shifting” effect was observed by other groups when analyzing molecular dynamics trajectories [29, 43] or conformational ensembles [83] by essential dynamics (ED). Fewer ED-modes captured more of the structural variance (i.e. relative amplitude of deformations) than ENM-modes. Hence, the absence of observed breaking contacts makes relevant deformations accessible.

#### e. Relationship between observed breaking contact occurrence and effect on ENM accuracy

To the best of our knowledge, *mc*ENM is the first approach to examine the effect of observed breaking contacts on ENM accuracy. Above we showed that they are a novel source of information, which helps to capture localized, functional transitions with ENMs. To further explore their importance we now analyze how their occurrence and impact are linked by considering motion type and structural fold of the proteins.

**Fig 3:**
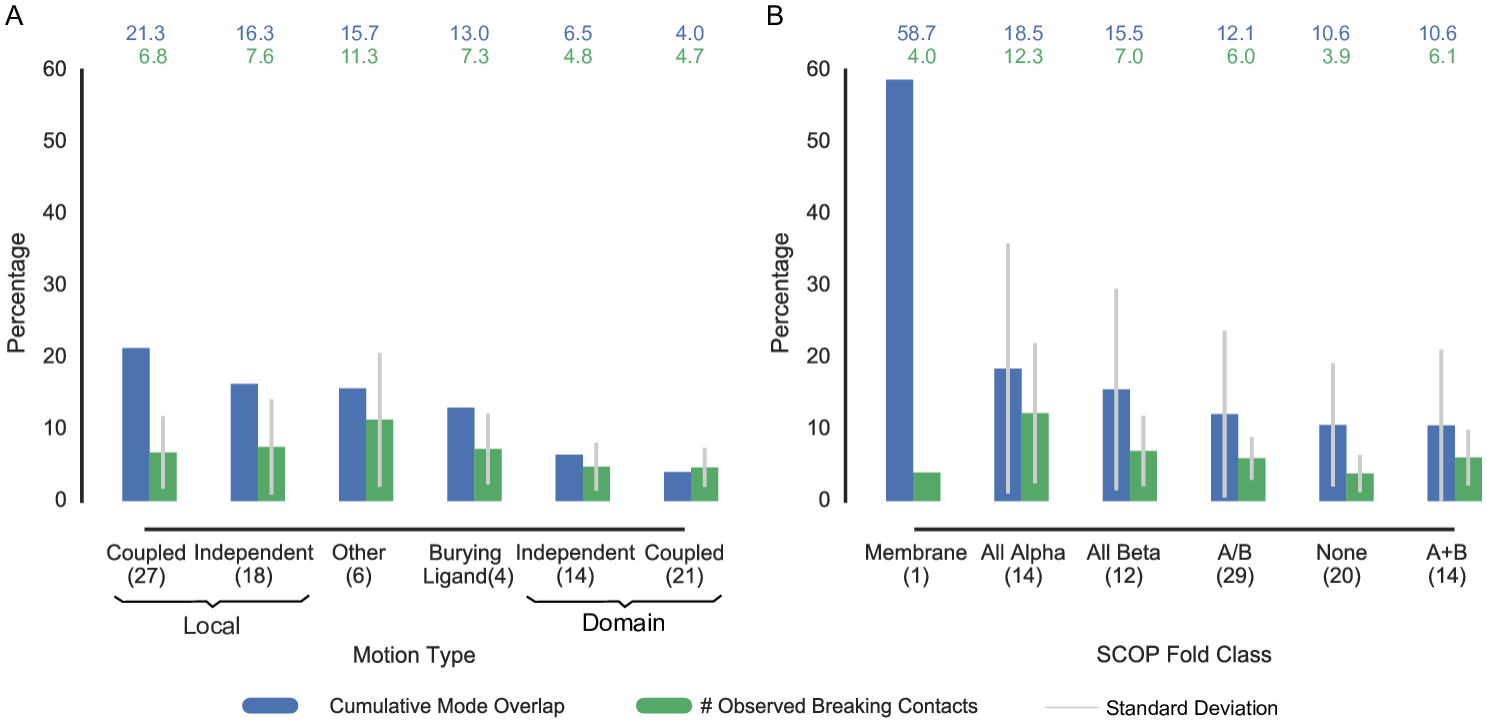
Accuracy improvement of *mc*ENM over ENM in relation to percent of observed breaking contacts on whole data set (90 proteins). The blue bars depict the absolute accuracy improvement of *mc*ENM over ENM averaged over each group, whereas the green bars show the average amount of removed breaking contacts. The accuracy improvement is calculated by the difference between cumulative mode overlap of the first ten low-frequency modes of *mc*ENM and ENM. (A) Proteins grouped by motion types. (B) Proteins grouped by SCOP fold class.

**Dependence on Motion Type** Fig 3A shows the accuracy improvement of *mc*ENM over ENM related to the amount of removed breaking contacts per motion category. In this analysis we also include burying ligand and other types of motions next to local and domain motions (see Methods for details on the motion classification).

*mc*ENM improves much more in accuracy for local motions than for domain motions given the amount of removed breaking contacts. Hence, individual breaking contacts seem to encode more information about motion when they belong to local movers than to domain movers. Surprisingly, also proteins that bury a ligand in their end conformation benefit from removing observed breaking contacts. This is particularly interesting as these proteins show only subtle differences between start and end conformation (< 1 Å RMSD), but must transiently open the entry to the binding site for the ligand to enter. Visual inspection reveals that–in at least one of the four cases–most observed breaking contacts locate at the proposed entry to the binding site in literature (Fig C in S1 File). Nonetheless, further research is needed to examine the relevance of observed breaking contacts for burying ligand and other motion types.

**Dependence on Structural Fold** The structural topology of proteins largely governs their dynamic behavior. Therefore, we analyzed if certain fold types promote contact changes more than others. Fig 3B summarizes the results for the proteins in our data set grouped by their SCOP class that we obtained from the Structural Classification of Proteins (SCOP) database [84, 85].

Remarkably, the only membrane protein in our data set improves by almost 60% in cumulative overlap despite a relatively small amount of breaking contacts (more details in h. Case Studies). *All alpha* proteins benefit more from removing breaking contacts than the remaining classes, although individual breaking contacts seem to have less impact than for the other classes. This may be due to the relatively high structural flexibility of *all alpha* proteins. Thus, breaking contacts may even occur in regions not necessarily related to the functional transition, making them less relevant. In contrast, folds strongly stabilized by a central beta sheet or beta barrel as in the *all beta, a/b*, or *a+b* classes appear to be more robust towards changes in the contact topology. This may lead to fewer, but larger clusters of breaking contacts having stronger impact on the improvement in accuracy. However, further investigation of these hypotheses is beyond the scope of this paper and will be addressed in future research.

#### f. Summary

Our results demonstrate that *mc*ENM substantially improves over ENM in explaining localized functional transitions simply by releasing erroneous constraints in form of observed breaking contacts. Without increasing the complexity of the underlying model, *mc*ENM seems to generalize much better than ENM. We also showed that the absence of observed breaking contacts narrows down the relevant deformation space substantially. However, *mc*ENM cannot be used to predict protein motions because to determine the breaking contacts it requires a known end conformation, which is usually not available. Our novel elastic network model, *lmc*ENM, aims to overcome this lack of knowledge by predicting breaking contacts instead of observing them. The following second part of our results evaluates our proposed approach.

### B. Evaluation of *lmc*ENM–Elastic network of learned maintained contacts

*lmc*ENM is built in three steps: (i) we predict the most likely breaking contacts with our machine learning based classifier, (ii) we choose a highest scoring subset of contacts, and (iii) we remove them from the initial contact network of the unbound conformation.

Therefore, we evaluate *lmc*ENM as follows: First, we identify the best strategy to select an appropriate subset of top-scoring predicted breaking contacts. Given this subset of contacts we then evaluate the ability of our classifier to identify correct and, most importantly, relevant breaking contacts. Next, we assess the performance of *lmc*ENM w.r.t the baseline ENM, the theoretical upper bound reached by *mc*ENM, and three reference ENM variants based on pairs of conformations as well as conformational ensembles. After presenting two case studies selected from our data set, we analyze which features contribute the most to a correct classification. We conclude this section by discussing limitations and potential applications of our approach.

The chosen reference ENMs exploit different sources of information to refine connectivity and stiffness of the network (see c. Additional ENM variants for evaluation): (i) HCA—a cutoff-free model with distance-dependent spring constants [41], (ii) OFC-ENM—a model analyzing structural properties of NMR ensembles to optimize force constants for secondary structure elements [45], and (iii) edENM—a hybrid model using a combination of bond-cutoff strategy in the local sequential neighborhood and distance-dependent force constants to model remote interactions [29].

#### a. Choosing how many top scoring predicted contacts to remove

Our classifier predicts for all contacts separated by at least four sequence positions their likeliness to break. Removing predicted breaking contacts with shorter range yielded unstable networks for both, *mc*ENM and *lmc*ENM (see Results Part I a. Experimental Design and Parametrization). From this rank-ordered list we need to choose how many top-scoring contacts should be removed for a particular protein. The protein’s motion type would be a good indicator to estimate this number as we showed above. But obviously this is unknown a priori. Therefore, we considered three simple selection strategies (see Methods for details) based on a: (i) constant cutoff, (ii) relative cutoff (percent), or (iii) score-dependent cutoff.

For each strategy we evaluated how it affects *lmc*ENM accuracy along a range of cutoff values. To facilitate a fair comparison we determine the best cutoff for each strategy as the one that maximizes the average over all proteins in our data set. We empirically find that the top *n* = 60 (constant cutoff), top *n* = 16% (relative), and SVM score > 0.4 (score-dependent) work best in our settings. Fig D in S1 File shows the accuracy distribution of each strategy grouped by protein motion type.

Overall, the strategies perform similar with small advances for the relative-cutoff strategy in three out of the four depicted motion categories. Proteins with independent local motions would rather benefit from the constant-cutoff strategy. We attribute this to the fact that, on average, less breaking contacts are removed by using the constant cutoff than with the relative cutoff. Given that our classifier is less accurate for proteins with independent local motions also fewer false positive predictions are removed, thereby reducing the chances of negatively affecting ENM accuracy. However, given its slightly better overall performance, we chose the relative-cutoff strategy and build *lmc*ENM by removing the top16% predicted breaking contacts. Table B in S2 File reports contact statistics for each protein, such as initial number of contacts and removed breaking contacts for both, *lmc*ENM and *mc*ENM.

We also performed a control experiment by removing the same amount of randomly selected contacts from the initial contact topology of the proteins. As expected we found no accuracy improvement over the unmodified ENM (Table C in S2 File).

Our results indicate that finding a good selection strategy most likely depends on more factors besides protein motion type and classifier performance. Nonetheless, even such a simple strategy as our chosen one already leads to substantial accuracy improvements of *lmc*ENM.

#### b. *lmc*ENM finds correct and relevant breaking contacts

Given the above chosen fraction of top-scoring breaking contacts, we now can evaluate the SVM classifier. We use the common measures precision (Prec = TP/(TP+FP)) and coverage (Cov = TP_*frac*_/TP_*all*_), where TP denote true positive, and FP false positive predicted breaking contacts. TP_*frac*_ are the true positives among the selected fraction, whereas TP_*all*_ is the total number of true positives for a protein. Furthermore, we report the area under the receiver operator characteristic (ROC) curve (AUROC) [86]. It estimates the probability of scoring a positive sample higher than a negative one if both are chosen randomly. An AUROC of 1 indicates a perfect predictor, a value of 0.5 refers to a random predictor.

**Fig 4:**
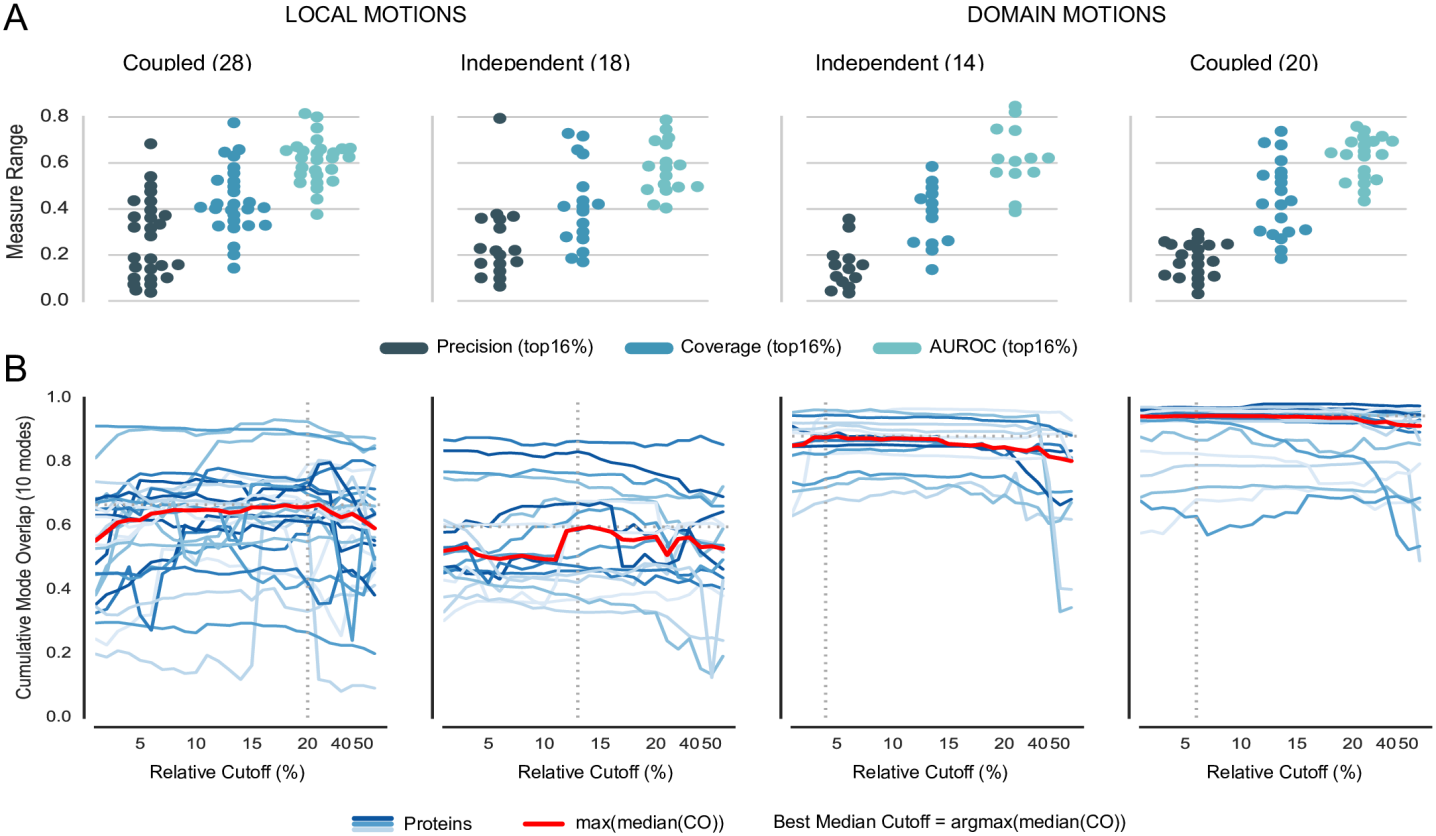
Classifier performance and sensitivity analysis of breaking contacts selection strategy, subset of local and domain motions (80 proteins). (A) Performance evaluation of classifier based on top16% predicted breaking contacts. The panels show precision, coverage, and area under receiver operator characteristic (AUROC) as swarmplot for each motion category. (B) Dependence of *lmc*ENM accuracy on removed topN% predicted breaking contacts ranked by decreasing SVM score. The blue lines depict how the *lmc*ENM-accuracy evolves for individual proteins when gradually removing more breaking contacts from their network. The cumulative mode overlap of protein with local motions often “jumps” upwards indicating a higher relevance of the removed breaking contacts responsible for this increase in accuracy. Accuracy drops if too many breaking contacts have been removed.

Fig 4A shows the prediction performance of the classifier along the protein motion types (see Table M in S1 File for individual results). Overall, the precision of the classifier is rather low. However, proteins with coupled local motions show higher precision on average. Interestingly, for some proteins–mostly domain movers–coverage is good despite a low precision. The fact that these proteins possess rather few observed breaking contacts might increase the chances of a TP among the top16% selected contacts.

We also performed a sensitivity analysis to test whether some predicted breaking contacts are more relevant for capturing the functional transition than others. Starting from the top1% until the top50% breaking contacts, we gradually removed more predicted contacts, while evaluating the reached accuracy. Fig 4B shows the results for proteins with local and domain motions. Most steps yield only small accuracy improvements. But sometimes, they cause a “jump” to a significantly higher or lower value. Drops in accuracy most likely result from removing too many false positive predicted breaking contacts. In contrast, large improvements indicate that the causing breaking contacts either are more relevant than the previously removed ones or that they allowed to reach the “critical mass”. In particular, proteins with coupled local motions show the largest jumps in *lmc*ENM accuracy.

Hence, despite its deficiencies in precision and coverage, our classifier seems to be able to identify breaking contacts not only correct but also relevant to improve *lmc*ENM accuracy.

#### c. Learned breaking contacts matter

Now we evaluate how much *lmc*ENM improves over ENM compared to the theoretical maximum reached by *mc*ENM.

**Fig 5:**
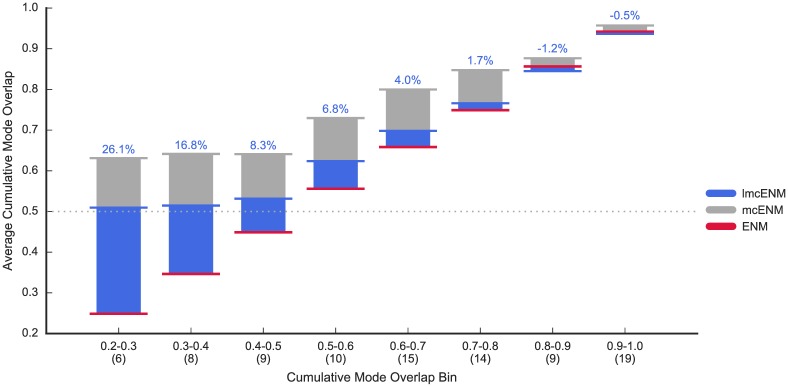
Accuracy of *lmc*ENM (our method) compared to ENM (baseline) and *mc*ENM (theoretical upper bound) on our data set (90 proteins). The accuracy is measured by the cumulative mode overlap of the first ten low-frequency normal modes (*CO*(10)). Proteins are binned based on the cumulative mode overlap reached by ENM (number of proteins per bin is given in brackets). The horizontal blue, gray and red lines mark the average accuracy per bin of *lmc*ENM, *mc*ENM, and ENM, respectively (numbers above each bin denote the absolute improvement of *lmc*ENM over ENM in percent). *lmc*ENM is most effective for proteins that largely remain elusive for ENM (*CO*(10) < 0.6). It is on par with ENM for the remaining proteins that are already accurately explained by ENM.

Fig 5 summarizes the results for the proteins binned by cumulative mode overlap of the first ten low-frequency modes (extended version of Fig 1, see Table C in S2 File for individual results). Overall, *lmc*ENM substantially outperforms ENM in accuracy, in particular, for proteins poorly captured with ENM (more than 60% of the theoretical maximum improvement on average). Individual accuracy improvements range between 1.5% up to 59.8% and sometimes even exceed the theoretical maximum reached by *mc*ENM (Table C in S2 File). As expected, proteins well captured by ENM benefit less from *lmc*ENM. We also find that *lmc*ENM substantially increases the number of proteins reaching 60% coverage of the functional transition with only ten lowest-frequency normal modes, albeit not as much as *mc*ENM (theoretical upper bound) (*lmc*ENM: 78% of proteins, ENM: 63%, *mc*ENM: 92%, see Table C in S2 File).

The overall improvement of *lmc*ENM by 5.5% on average (4.5% median) over the baseline ENM might appear small given the computational overhead of the machine-learning based classifier (Table C in S2 File of the supporting material). However, in relation to the performance of the reference ENMs (OFC-ENM: 0.95%/-1.35%(mean/median), edENM: 0.83%/-1.0%, HCA: 1.24%/-0.10%) on our data set it becomes evident that general applicability of ENMs might require such additional computational costs.

For eight proteins, *lmc*ENM accuracy drops notably below the baseline (more than -5.0%) (Table O in S1 File and Table C in S2 File). Two of them (PDB_IDs: 2v8iA, 1lfhA) are domain movers. In both cases, *lmc*ENM removes too many contacts due to the chosen selection cutoff (top 16% predicted breaking contacts). With an optimal selection cutoff removing fewer contacts, *lmc*ENM would perform as good as ENM (Table P in S1 File). Nonetheless, the performance of *lmc*ENM is still good (above 0.85 CO(10) for both proteins). Also for three other cases (PDB_IDs: 1gohA, 1a8dA, 1kp9A)–all local movers–the optimal selection cutoff would yield comparable performance of *lmc*ENM. Notably, 1kp9A, is the only case with SVM precision and coverage above average of the motion category. Yet even with an optimal selection cutoff it would not improve over ENM. Given that *mc*ENM improves over ENM by 5.9% *lmc*ENM most likely predicted breaking contacts that were correct but not relevant. Fig E in S1 File (leftmost panel) supports this view by the continuously decreasing cumulative overlap when gradually removing more predicted breaking contacts. In particular, for proteins with independent local motions or domain motions a better selection strategy may help to reduce the overall amount of removed breaking contacts, thereby decreasing the number of false-positive removed contacts (see the marked best median cutoff for individual motion types in Fig 4).

For the remaining three cases (PDB_IDs: 1dx9C, 2dh3B, 2jepB) even the optimal selection cutoff yields between 1.2% and 5.5% lower cumulative mode overlap than the baseline ENM. Fig F in S1 File shows the networks with breaking and maintained contacts for 2dh3B accompanied by a plot depicting the fluctuation profiles of the different ENM variants scaled to the observed displacements. Although *lmc*ENM partially captures true-positive breaking contacts, it misses observed ones (indicated by the dark arrows) in particular at the interface between two helices in the center performing a shear motion as well as between their connecting loop and the right helix (arrow a2). Consequently, the flexibility of these regions is underestimated (mostly around the most flexible center of the loops), whereas it is largely overestimated around two solvent-exposed loops (arrow 4), where only few breaking contacts have been observed. Hence, our feature capturing the location (border vs center) of a contact on a loop seems to be not discriminative enough. The situation for the other two proteins is highly similar. We also note that four out of the eight cases are proteins with independent local motions, i.e. not coupled to a ligand. Apart from improved feature design training of an ensemble of SVMs may help to improve the performance of the classifier. Here, each SVM could be trained to capture specific properties of a single motion category, which are then combined into an ensemble of classifiers for prediction. Such ensemble classifiers were, for instance, successfully applied in the context of protein contact prediction [30].

Next to the mode overlap we also evaluated other metrics that are commonly used to assess the performance of ENMs. The performance of *lmc*ENM compared to all other ENM variants w.r.t. to these metrics is summarized in Table 3. *lmc*ENM consistently outperforms all other ENM variants (apart from *mc*ENM (theoretical upper bound)) in all metrics except for the correlation between temperature factors and maximum overlap (considering all modes). Detailed results are given in Table C in S2 File.

**Table 3:** Evaluated similarity measures for *lmc*ENM compared to ENM (baseline), *mc*ENM (theoretical upper bound) and three reference ENM variants.

Measure	ENM	OFC-ENM	edENM	HCA	*lmc*ENM	*mc*ENM
Cumul. Mode Overlap (10)	0.69/0.66	0.67/0.67	0.68/0.67	0.68/0.68	0.73/0.72	0.82/0.80
Cumul. Fraction of Variance (10)	0.35/0.38	0.40/0.43	0.57/0.59	0.34/0.36	0.60/0.60	0.57/0.59
CorrCoeff Fluctuations—Displacements (10)	0.52/0.50	0.52/0.52	0.52/0.50	0.52/0.50	0.58/0.56	0.81/0.78
CorrCoeff Temperature Factors—Betas (10)	0.40/0.40	0.41/0.41	0.48/0.46	0.45/0.44	0.41/0.40	0.41/0.40
Max Overlap	0.47/0.50	0.46/0.51	0.44/0.50	0.46/0.50	0.45/0.50	0.60/0.62
Rank (Max Overlap Mode)	1.00/11.08	1.00/15.71	1.00/49.72	1.00/32.81	1.00/2.80	0.00/1.93
Degree of Collectivity (Max Overlap Mode)	0.38/0.39	0.40/0.40	0.45/0.41	0.40/0.40	0.33/0.34	0.27/0.31
Fraction of Variance (Max Overlap Mode)	0.05/0.08	0.06/0.08	0.09/0.12	0.05/0.06	0.08/0.13	0.12/0.20
#Modes Cumul. Mode Overlap (70%)	11.00/34.51	12.50/29.72	11.00/30.09	10.00/29.29	7.00/23.37	3.00/6.92
#Modes Cumul. Mode Overlap (80%)	35.00/79.07	34.00/66.62	31.50/70.99	31.50/65.76	22.50/54.08	7.50/19.41
#Modes Cumul. Mode Overlap (00%)	164.50/200.60	119.00/165.86	128.00/187.02	105.50/175.33	100.50/156.79	39.00/75.56

Median/mean values are reported for each measure. For several measures we consider only the subset of the first ten low-frequency modes indicated by (10) after the measure’s name. Except for Rank (Max Overlap) and Collectivity higher values are better. A lower rank of the best overlapping mode with the observed displacement vector indicates that the most relevant motion captured by the elastic network also is more dominant. In terms of Degree of Collectivity, we find that lower values indicate that less collective, localized functional transitions are better captured (see next paragraph for more details).

*lmc*ENM improves over the other ENM variants in capturing motion directions (overlap, structural variance, number of modes to explain up to X percent cumulative mode overlap) as well as motion amplitudes (correlation between fluctuation profiles) of the functional transition. edENM reaches a comparable cumulative fraction of variance (10 lowest-frequency modes) and is the best method to explain experimental b-factor profiles with predicted temperature factors (squared residue fluctuations of the first ten low-frequency modes scaled to b-factors). We attribute this to the carefully optimized stiffness constants of edENM based on MD simulations. In terms of maximum overlap, all ENM variants reach similar values. A closer look at the results for different motion types reveals more variation as we will see in the following paragraph. Considering the fraction of variance explained by the best-overlapping mode *lmc*ENM and edENM perform the best on average. Although the median rank of the best-overlapping mode is 1 for all ENM variants, the average rank shows that *lmc*ENM effectively shifted the best-overlapping mode towards lower frequencies (*lmc*ENM: 2.8 (best), ENM: 11.1 (2nd best), *mc*ENM: 1.9). Interestingly, *lmc*ENM and *mc*ENM yield much lower degree of collectivity for the best overlapping mode, whereas the other ENM variants reach higher values compared to the ENM (baseline). Hence, they seem to better capture less collective, localized transitions, which we will further investigate in the next paragraph.

These results show that the selected, learned breaking contacts in fact contain valuable information to improve ENM accuracy. *lmc*ENM is most effective for proteins that are poorly captured by ENM indicating that it helps where needed the most.

#### d. *lmc*ENM is most effective for coupled localized functional transitions

**Fig 6:**
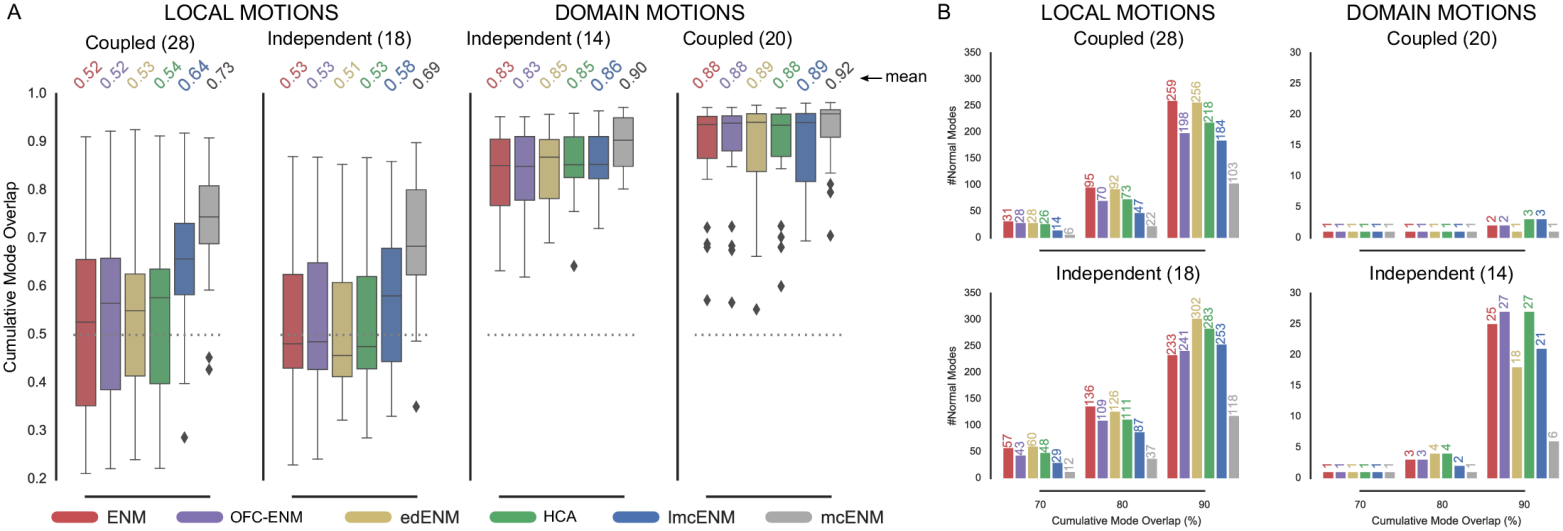
Dependence of accuracy (A) and dimensionality of deformation subspaces (B) of ENM (baseline), *lmc*ENM (our method), *mc*ENM (theoretical upper bound), and ENM on motion type of protein, subset of local and domain motions (80 proteins). (A) Accuracy is measured by the cumulative mode overlap of the first ten low-frequency normal modes (*CO*(10)). *lmc*ENM consistently improves over ENM in each motion category, being particularly effective for proteins with coupled localized functional transitions. (B) The panels show the median number of normal modes (spanning the deformation subspace) required to explain between 70% and 90% of the functional transition (measured in cumulative mode overlap (%)). *lmc*ENM consistently requires fewer modes to capture the same amount of conformational change as ENM.

Above we showed that observed breaking contacts matter to capture localized functional transitions (see A. ENMs and the Effect of Removing Observed Breaking Contacts). To evaluate whether this holds true also for the chosen predicted breaking contacts we analyze the performance of *lmc*ENM considering the motion type of the proteins. Fig 6A shows the results.

*lmc*ENM consistently outperforms ENM in accuracy regardless of the depicted motion type, being most effective for proteins with ligand-coupled local motions (*lmc*ENM: 12% improvement, *mc*ENM: 21%, HCA: 2%, edENM and OFC-ENM: 1% on average). Proteins with independent local motions improve less due to lower classification accuracy (see Fig 4A).

We also find that *lmc*ENM captures domain motions slightly better than other ENM variants or is on par despite the relatively poor classifier accuracy (see Fig 4A). We attribute this to the fact that proteins performing domain motions are structurally more rigid than proteins with local motions. Hence, the former seem to be more robust against removing false positive predictions given their higher chance to be a redundant constraint.

*lmc*ENM and edENM largely improve over the other ENM variants w.r.t. the total variances captured by the first ten low-frequency modes, with slight advances for *lmc*ENM (Fig G(A) in S1 File). By removing predicted breaking contacts *lmc*ENM effectively compensates for the overestimated rigidity in the baseline ENM [29]. Hence, the *lmc*ENM-modes with more relevance–as indicated by the larger cumulative mode overlap above–become easier accessible and contribute more to the total variance of the system. In the other ENM variants these modes are spread among a wider range, which decreases their individual contribution as well as their captured total variance. Given that removing breaking contacts is a purely topological change our work supports the findings by Orellana et al. [29] that such an effect cannot be achieved by refining spring stiffness alone.

Considering the correlation coefficients between predicted and observed fluctuations only coupled local and independent domain motions are better captured by *lmc*ENM, while it is on par with the other ENM variants for the other two motion types (Fig G(B) in S1 File). Experimental b-factors are best explained by edENM followed by HCA, while *lmc*ENM does not improve over the baseline ENM (Fig G(C) in S1 File). This can be explained by the fact that *lmc*ENM only adjusts the network topology without refining the stiffness of the springs that is typically tuned for ENMs to better match B-factor profiles. Also, larger distance cutoffs (>16 Å) are usually required to gain better agreement with experimental B-factors thereby increasing structural stiffness and collectivity of motion [70]. Given our focus on better capturing localized transitions with low degree of collectivity, this metric is of limited use in our context.

We also note that edENM improves little over the baseline ENM for coupled local motions and even drops below for independent local motions. This is unexpected given the reported performance of edENM in the original publication [29]. The main difference between *lmc*ENM and edENM is the protein-size dependent cutoff used by the latter to identify remote interactions. edENM also scales the stiffness constants depending on sequence or spatial distance. But this cannot explain the large difference in cumulative mode overlap between edENM and *lmc*ENM. Fig I in S1 File compares the best cutoff yielding largest cumulative mode overlap of the first ten low-frequency modes with the protein-size dependent cutoff of edENM. Most of the proteins do not follow the proposed logarithmic function. Consequently, the protein-size dependent cutoff seems to largely over-constrain the network for most proteins in our data set compared to the distance-cutoff used by the baseline ENM, *lmc*ENM, and *mc*ENM. This explains why edENM on average does not improve in cumulative mode overlap over the basic ENM for proteins with local function-related movements (Table C in S2 File).

Our results indicate that the predicted breaking contacts are in fact relevant to capture localized functional transitions, in particular if they are coupled to the binding of a ligand.

#### f. *lmc*ENM reduces dimensionality of essential deformation space

Above we showed that *mc*ENM substantially narrows down the essential deformation space of proteins used for subsequent fine-grained exploration (see p. 17). We cannot expect such a drastic dimensionality reduction for *lmc*ENM because the classifier only partially covers the observed breaking contacts and additionally outputs many false positives. Nonetheless there should be some effect.

Fig 6B shows the median number of modes required for each ENM variant to reach a cumulative overlap of 70%, 80%, and 90%. As expected, *lmc*ENM cannot compete with *mc*ENM. But– regardless of motion type–*lmc*ENM requires a considerable smaller amount of low-frequency normal modes than ENM to capture up to 90% of the functional transition, except for proteins with independent local motions. To reach 90% overlap *lmc*ENM needs more modes than ENM. Also the other ENM variants are able to reduce the number of required modes compared to the baseline ENM, albeit not as much as *lmc*ENM can do in most cases. For instance, to capture coupled local motions with 80% overlap *lmc*ENM needs only about half of the modes ENM needs (*lmc*ENM: 47, ENM: 95), whereas the next best other ENM variant is OFC-ENM with 70 modes. Reaching a desired overlap with fewer modes only works if individual modes capture more of the conformational transition. Hence, *lmc*ENM is able to reveal actually relevant modes particularly for coupled localized functional transitions.

Another way to investigate this is to analyze the best overlapping mode out of all modes. For highly collective protein motions usually a single low-frequency mode captures the movement quite well. Thus, large overlap and low ranking of this mode indicate that the ENM is able to accurately explain the movement. Localized functional transitions with low degree of collectivity (i.e. fewer residues are involved in the movement) require more modes (usually less than 10) to be captured [27]. These modes are often spread among higher frequencies yielding rather low overlaps in the low-frequency mode spectrum. Hence, in this case apart from higher overlap and lower rank also lower collectivity of the best-overlapping mode is desirable because it indicates that an actually relevant mode has been successfully shifted towards lower frequencies. We report the reached maximum overlap, the rank of this mode among all modes, the fraction of variance explained by this mode as well as its degree of collectivity (see Methods for details).

Fig H in S1 File shows the results. In particular for localized transitions *lmc*ENM improves in maximum overlap over the other ENM variants (except *mc*ENM). The best overlapping modes of *lmc*ENM not only have much lower rank but also contribute more to the structural variance compared to the other ENM variants. This is because they more likely represent a localized transition given their lower degree of collectivity. The even lower collectivity and corresponding higher maximum overlap of *mc*ENM indicates that *lmc*ENM missed to capture some of the localized transitions. In contrast, for domain motions *lmc*ENM shows smaller maximum overlap. Especially for independent domain movers the best overlapping *lmc*ENM-modes are less collective compared to the other ENM variants, which is not desired for this motion type. This can be explained by the fact that due to the chosen selection cutoff *lmc*ENM removes much more predicted breaking contacts than optimal for this class of proteins (see Fig 4B, not for coupled domain movers). As a consequence, actually irrelevant movements with low degree of collectivity become accessible and may contribute more to the predicted deformability than the relevant collective ones. But despite this smaller maximum mode overlap *lmc*ENM still outperforms the other ENM variants in cumulative mode overlap as shown above (see p. 23).

Taken together these results show that *lmc*ENM effectively “shifts” modes that are relevant to explain localized motions towards lower frequencies. Nonetheless, in several cases *lmc*ENM still requires more than 100 modes to capture 70% of the conformational change (see for instance 1a8dA (*lmc*ENM: 102, ENM: 136, OFC-ENM: 112, edENM: 121, HCA: 108, *mc*ENM: 33) or 1bsqA (*lmc*ENM: 126, ENM: 219, OFC-ENM: 155, edENM: 114, HCA: 179, *mc*ENM: 21) in Table C in S2 File). In such cases neither of the evaluated ENM variants is able to significantly narrow down the essential deformation space, which is the actual advantage of ENMs over other prediction methods in particular for proteins with collective motions. However, *mc*ENM (based on the removal of observed breaking contacts) clearly demonstrates that this advantage does exist also for the cases that are difficult to capture by standard ENM (less than 20 modes suffice to reach CO70% for 83/90 proteins). We only need to predict the relevant breaking contacts correctly. *lmc*ENM is able to do so for 67/90 proteins, which is an improvement of 11% over the second best method, OFC-ENM, that is successful in 57/90 cases. Thus, we believe that *lmc*ENM–despite its current limitations–provides the necessary means to advance the predictive power of ENMs for yet poorly captured proteins.

#### e. Validating against essential dynamics of conformational ensembles

Finally, we validate our method against the structural flexibility captured by redundant conformational ensembles. Due to the rapid growth of the Protein Data Bank (PDF) such ensembles recently emerged as valuable source characterizing the conformational diversity around the native state [76–78]. Amongst others, the CoDNaS 2.0 database [78] provides such a redundant collection of conformers obtained under different conditions for the requested protein. To adequately capture the native conformational diversity a minimum ensemble size of ten is recommended [76, 78], which we were able to retrieve for 35 proteins in our dataset (see Table D in S2 File). Principal component analysis (PCA) identifies the Essential Dynamics (ED) captured by the conformational ensembles, which can be compared to the normal modes of ENMs.

Fig J in S1 File shows how well *lmc*ENM explains the native conformational diversity compared to the other ENM variants. Detailed results for each protein can be found in Table E in S2 File. We measure the similarity of PCA space and ENM spaces by (i) comparing fluctuation profiles of the first ten low-frequency modes (A), (ii) the subspace overlap (also called RMSIP10) of the same mode set (B), and the weighted overlap (RWSIP) of both spaces (C). While the first two measures only consider the agreement in either magnitudes or directions of motion, respectively, the latter accounts for their interplay. In addition, RWSIP has no limit on the size of the compared spaces. Despite the wide use of RMSIP, RWSIP is considered the more comprehensive measure to assess vector space similarity [80, 87]. In fact, all ENM variants reach comparable subspace overlap thereby limiting the information gain of RMSIP. The comparison of slow-frequency fluctuation profiles reveals rather small advances for *lmc*ENM except for coupled domain motions, where *lmc*ENM performs even slightly worse than the baseline ENM. However, in terms of RWSIP *lmc*ENM clearly performs the best, followed by edENM and OFC-ENM.

These results confirm the outcome of the previous experiments. To further improve the performance of *lmc*ENM it may help to extract additional features from conformational ensembles from various sources (e.g. X-Ray, NMR, MD simulations). For instance, simple counting of contact occurrence in predicted candidate protein structures is a very successful method for *ab initio* protein contact prediction [88].

#### g. Summary

Our novel elastic network model, *lmc*ENM, relies on predicted instead of observed breaking contacts. Despite this lack of knowledge *lmc*ENM substantially outperforms all other ENM variants in explaining localized functional transitions, in particular if they are coupled to ligand binding. We showed that the predicted contacts by our classifier are not only correct but relevant to capture these movements with low degree of collectivity. Also proteins with domain motions benefit from the absence of the predicted breaking contacts, albeit to a much smaller extent. Furthermore, *lmc*ENM narrows down the number of low-frequency modes required to capture a desired amount of conformational change, which reduces the computational cost of guiding fine-grained conformational exploration. Without increasing the complexity of the underlying model, *lmc*ENM offers a promising route towards improving the general applicability of ENMs.

#### h. Case studies

In the following we discuss the performance of *lmc*ENM in more detail on two biologically interesting proteins selected from our data set: the outer membrane transporter FecA and Arachidonate 15-Lipoxygenase.

**FecA** The outer membrane protein has two main functions: To actively transport iron (ferric citrate) into the cells of *Escherichia coli* through their outer membrane [89]. Second, to trigger the transcription of genes responsible for the iron uptake. Fooling this iron-transport mechanism allows to infiltrate antibiotics into the cells of multi-drug resistant bacteria, which makes FecA a biologically interesting target [90]. We picked FecA for this case study because—despite being the only membrane protein in our data set—*lmc*ENM captures its functional transition almost 40% more accurate than ENM.

**Fig 7:**
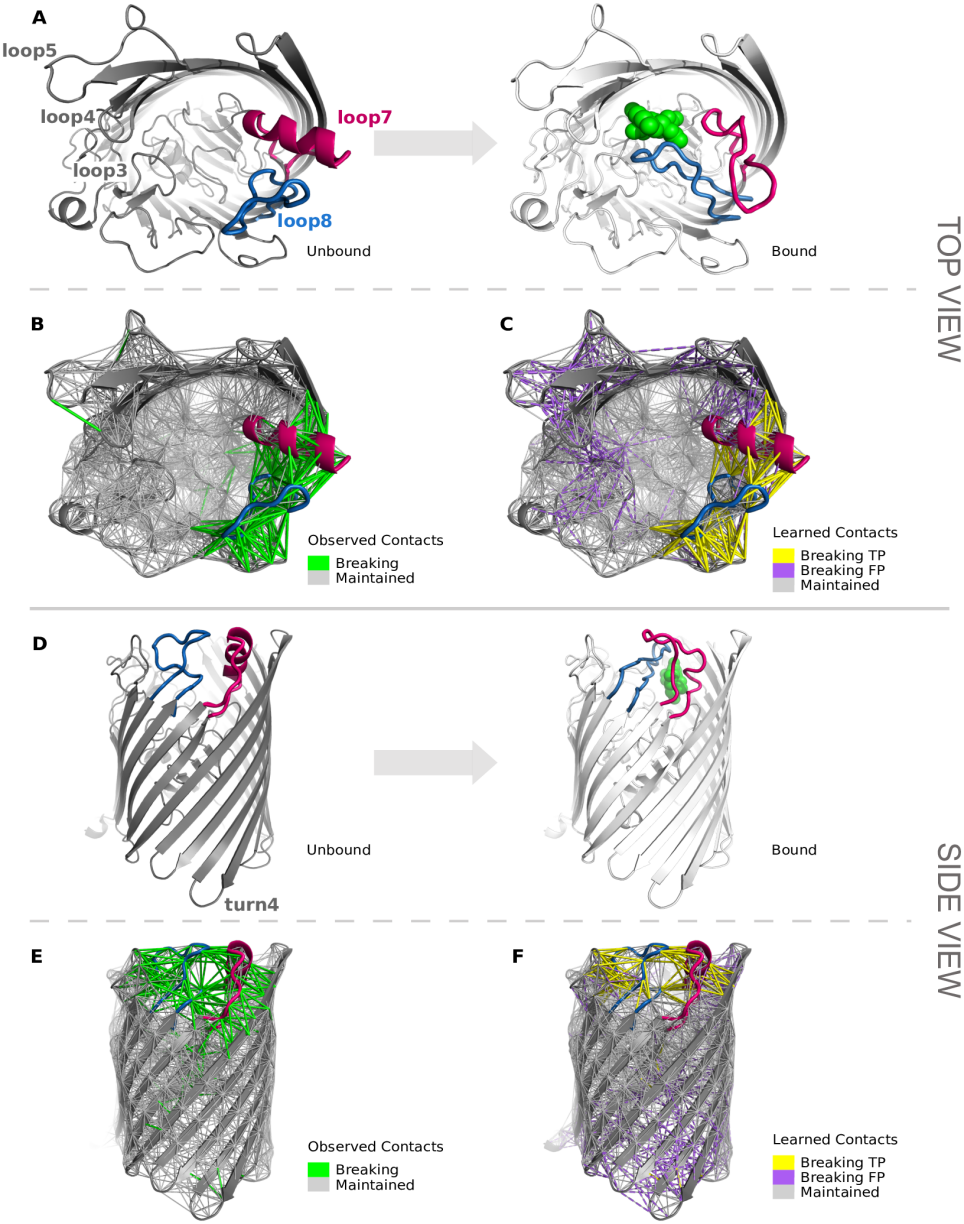
Conformational transition of outer membrane transporter FecA compared to observed and learned changes in its contact topology. (A,D): Function-related movement from unbound to bound conformation. The highlighted loops 7 (red) and 8 (blue) move the most to cover the ligand (green spheres) in the binding pocket. (B,E) *Observed* contact network of the unbound conformation mostly residing around the two highlighted loops. (C,F) *Learned* contact network. True positive (TP) predicted breaking contacts accurately match the observed ones around loop 7 and 8. The top view (C) reveals a cluster of false positive (FP, violet) predictions around loops 3, 4, and 5. Between loop 4 and 5 a single breaking contact is observed, which is not predicted. Some more FP breaking contacts are predicted around the plug domain within the *β*-barrel and turn 4 at the bottom of the barrel (F). For clarity, we omit drawing short-range contacts (sequence separation < 4 residues).

FecA is a three-domain protein [89] consisting of (i) a *β*-barrel spanning the membrane, (ii) a “plug” domain comprised by a mixed four-stranded *β*-sheet blocking direct diffusion through the barrel, and last an NH-domain in the periplasm (not resolved in the crystal structure). Fig 7A and 7D, depict the functional transition of FecA marked by unbound and ligand-bound conformation (PDB-ids: 1pnzA [91] and 1kmpA [89]). Two large extracellular loops (7 and 8) of the *β*-barrel dominate the transition by covering the ligand in the binding site [89, 92]. Being propagated through the plug-domain these movements then cause an unwinding of the H1-helix (“switch” helix) to trigger the gene transcription process.

Fig 7B and 7E, reveal that initially both loops are tightly constrained within the contact network of the unbound conformation. However, most of these surrounding contacts are observed to break (highlighted in green) to facilitate the major conformational changes of loops 7 and 8. Remarkably, the learned breaking contacts (true positives (TP), yellow) closely resemble the observed ones in the most relevant core region of both loops. Only towards their less flexible anchor points fewer contacts have been predicted to break (Fig 7C and 7F).

However, we also notice many false positive predicted breaking contacts (FP, violet) that have not been observed, for instance around loops 3, 4, and 5 (Fig K(A) in S1 File). Interestingly, there is a single observed breaking contact between loops 4 and 5, which indicates that a more strict extension threshold would have identified more contacts as breaking around these loops (Fig 7B). In fact, for this protein the optimal extension threshold to identify observed breaking contacts for *mc*ENM would be 3% (CO10: 0.839) instead of the used 9% (CO10: 0.809), which is the optimal threshold determined for the whole data set (tested for distance cutoff within 8-18Å and extension thresholds between 3 and 25%). Based on this optimal extension threshold the agreement between predicted and observed breaking contacts would improve, in particular in loop 4 and 5 (Fig L in S1 File). Hence, the predicted increased flexibility for these loops may be actually correct. This hypothesis is supported by the structural differences between these two loops [89] as well as the fast fluctuations of loop 5 revealed by MD simulations in order to interact with membrane environment and ligand [92].

Additional false positive predicted contacts locate between plug-domain and *β*-barrel, and around the switch-helix (Fig K(B) in S1 File). To enable the passage of the ligand through the protein the plug-domain is supposed to move within the *β*-barrel [89], yet MD simulations revealed only small positional changes [92]. Also, the switch-helix, not captured in the bound conformation, transiently unfolded in MD simulations [92]. Taken together, our results indicate that our classifier might generalize much better than indicated by its relatively low prediction accuracy over the full data set (Table M in S1 File).

**Fig 8:**
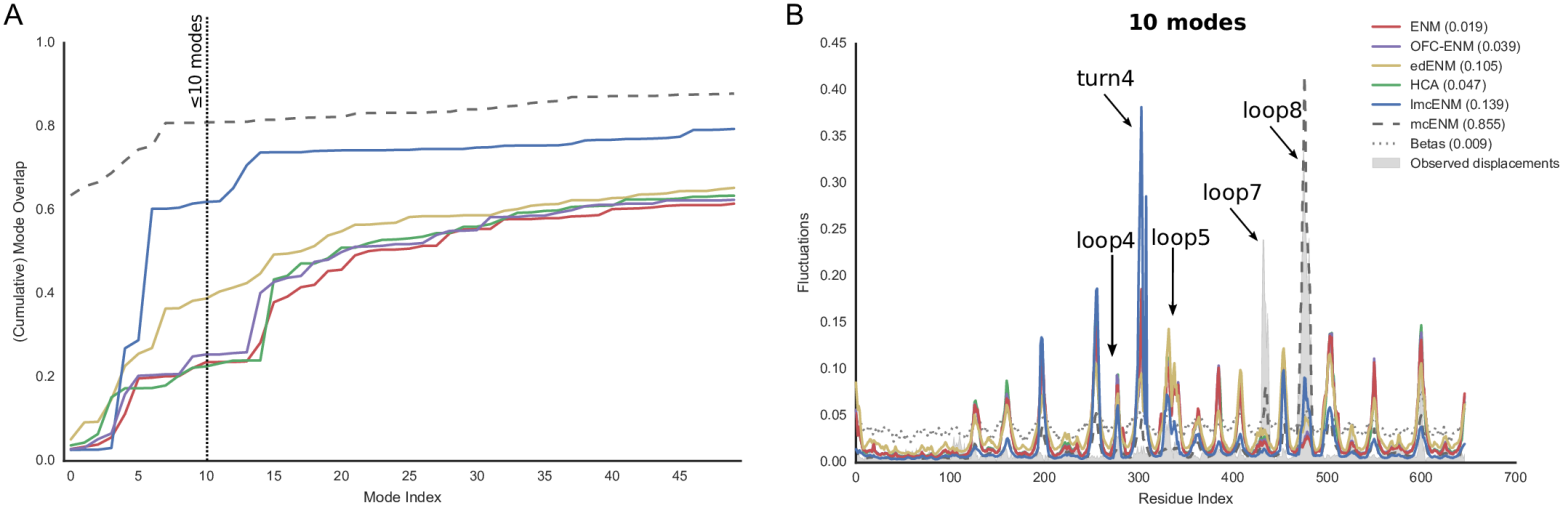
Cumulative mode overlaps and fluctuation profiles of *lmc*ENM, *mc*ENM, and the reference ENM variants for FecA. (A) Reached cumulative overlap (curves) of the first 50 normal modes with the conformational transition. The bars depict how much of the movement individual modes capture. *lmc*ENM largely outperforms the baseline ENM and the reference ENM variants (color coding is the same as in panel B). The vertical dotted line marks the cumulative mode overlaps reached with the first ten low-frequency modes. (B) Residue fluctuations along the first ten low-frequency modes scaled to fit the observed displacement magnitudes (filled gray curve) between the two conformations. The Pearson correlation coefficient is given in brackets behind the ENM labels. *lmc*ENM resembles the higher flexibility of loop 8 more accurately than ENM and other ENM variants, but largely underestimates the flexibility of loop 7. Also, loops 4 and 5 are captured well by *lmc*ENM. But due to the removal of too many false positive predicted breaking contacts (see Fig 7F), *lmc*ENM largely overestimates the flexibility of turns 4 and 3 connecting the strands at the bottom of the *β*-barrel.

We also analyzed how accurate *lmc*ENM predicts the motion directions compared to the other ENM variants. Fig 8A shows the cumulative mode overlap of the top 50 lowest-frequency modes. With the first ten modes *lmc*ENM explains more than 60% of the functional transition, an improvement of 40% compared to the baseline ENM and other ENM variants. Only edENM captures almost 40% of the movement, but eventually aligns with the other ENMs significantly below *lmc*ENM when considering more modes. This shift of relevant modes towards lower frequencies also becomes evident w.r.t. the lower rank of the best-overlapping mode (*lmc*ENM: 6, ENM: 15, edENM: 7, *mc*ENM: 0) and reduced number of modes required to capture, for instance, 70% of the cumulative overlap (*lmc*ENM: 13, ENM: 187, edENM: 82, *mc*ENM: 3). The improvement of *lmc*ENM over the baseline ENM and the reference ENMs is consistent for all evaluated measures (Table C in S2 File).

While the mode overlap of *lmc*ENM seems to be robust against false positive predicted contacts, they clearly have negative impact on the correlation of predicted and observed fluctuation patterns. Fig 8B shows that only *mc*ENM is able to capture the observed displacement magnitudes. All other ENM variants, including *lmc*ENM, reach poor agreement with the observed fluctuations. In particular, turns 4 and 3 connecting the strands at the bottom of the *β*-barrel become way too flexible due to the removed false positive predicted breaking contacts in *lmc*ENM. Also the other ENM variants overestimate the flexibility of these turns. Despite the many true positive breaking contacts around loop 7 the SVM classifier missed relevant observed ones towards the anchor points (Fig 7F) and within the helical part, which unfolds completely in the bound conformation. Such an unfolding of helical parts of a loop is currently not explicitly captured by our features. Instead the classifier treats the helix-like part as rather stable.

However, *lmc*ENM has lower tendency to overestimate the flexibility of the other loops and turns than the other ENMs. This together with the closer match of the highly flexible extracellular loop 8 accounts for the slightly higher correlation of *lmc*ENM with the observed fluctuations. One way to reduce the amount of false positive predictions could be to filter the predicted contacts using corroborating evidence. Predicted breaking contacts close to each increase their individual likelihood to be a correct prediction. The SVM classifies each contact individually without knowing whether contacts in the neighborhood have been assigned a high probability to break. We will elaborate this further in future research.

Nonetheless, the overall performance of *lmc*ENM for FecA w.r.t. to all other metrics is remarkable given that it is the only membrane protein in our data set. Even though our SVM-classifier was not specifically trained on membrane proteins it correctly predicted relevant breaking contacts. This indicates that proteins may share similar local structural parts that are involved in similar movements although they differ in their overall structure. In fact, previous work proposed that protein dynamics and deformation patterns may be evolutionary conserved and shared among proteins [93–96]. However, further research is required to confirm this hypothesis.

**Arachidonate 15-Lipoxygenase (15S-LOX1)** This protein belongs to a class of fatty acids oxidizing enzymes that are involved in inflammatory diseases. Understanding how these enzymes move may advance successful inhibitor design [97]. 15S-LOX1 is a two-domain protein exhibiting domain *and* local conformational changes. But only the local motions within the larger, catalytic domain enable the ligand binding [97]. Our results show that *lmc*ENM explains this functional transition even more accurate than *mc*ENM (theoretical maximum) with the first ten low-frequency modes, thereby substantially outperforming all other ENM variants.

**Fig 9:**
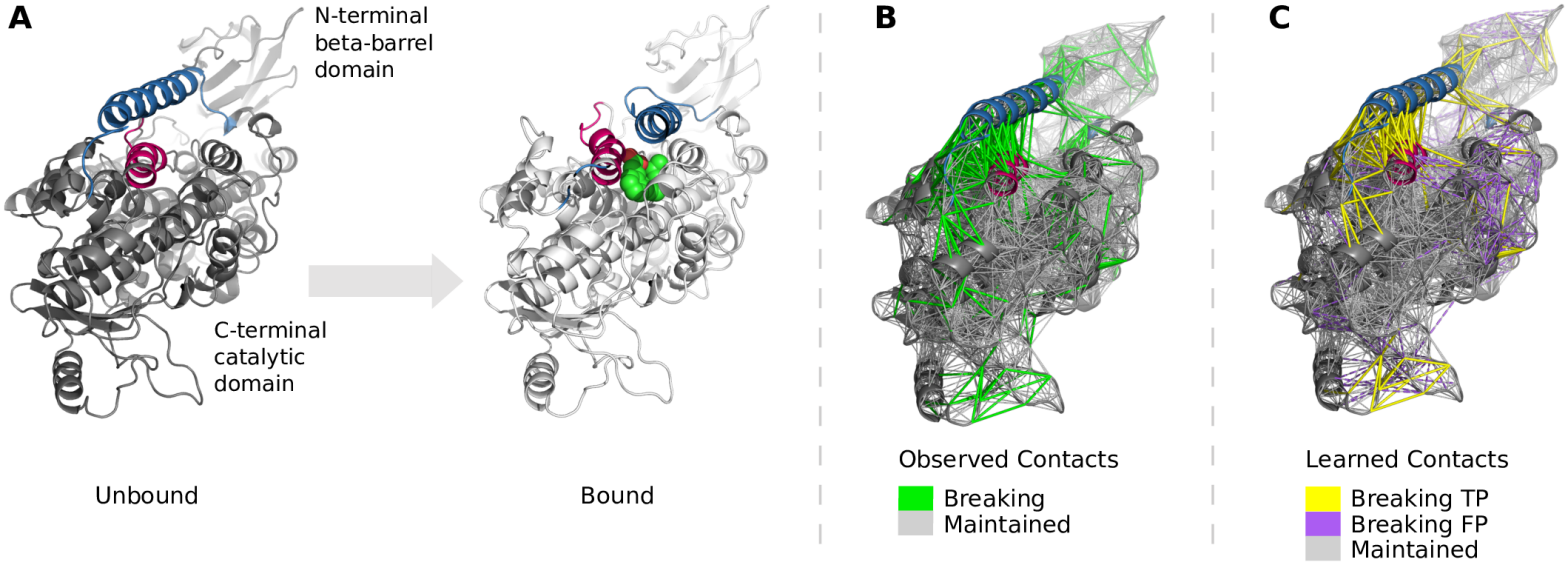
Conformational transition of Arachidonate 15-Lipoxygenase compared to observed and learned changes in the contact topology. (A) To accomodate the ligand (green spheres) in the binding site mostly the two highlighted helices (blue and magenta) move between unbound and bound conformation. (B) Most *observed* breaking contacts reside at the interface of the *α*2-helix (blue) to the rest of the structure. (C) The *learned* breaking contacts match most of the observed ones near the two helices. Most false positive contacts are predicted between the two domains, which seems actually be correct given the high mobility of the N-terminal domain in MD simulations.

Fig 9A depicts unbound and bound conformation (PDB-ids: 2p0mA and 2p0mB [97]) of the functional transition. Accomodating the ligand in the narrow pocket mainly requires movement and partial unfolding of the two highlighted helices (proposed induced-fit mechanism) [97]. Not surprisingly, most observed breaking contacts reside around these helices (Fig 9B). Our method correctly predicts most of the observed breaking contacts, but overestimates the occurrence of breaking contacts (false positives, FP) in other parts of the network (Fig 9C) and in particular between the two domains (Fig M in S1 File). Although the domain motion is not captured by the X-ray conformations, MD simulations reveal large inter-domain movement. Hence, the FP breaking contacts between the two domains seem to be correct. The other false positives around solvent exposed loop regions indicate that our classifier may overemphasize the relevance of such loops.

**Fig 10:**
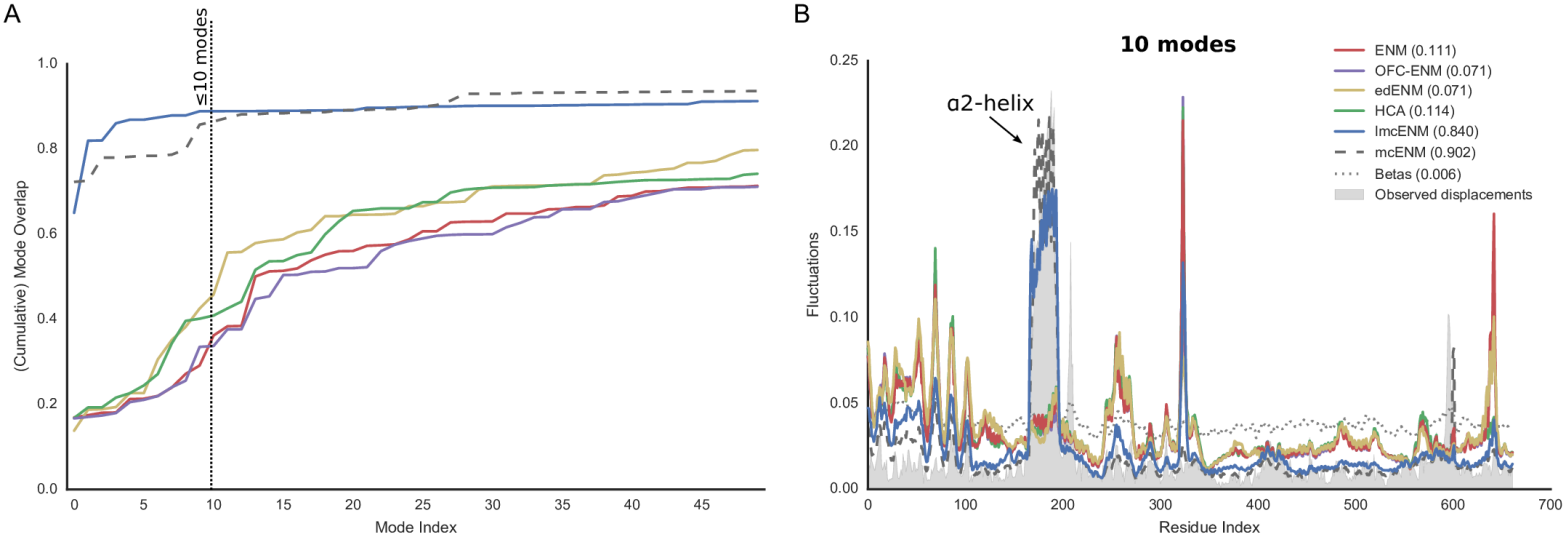
Cumulative mode overlaps and fluctuation profiles of *lmc*ENM, *mc*ENM, and the reference ENM variants for 15S-LOX1. (A) Reached cumulative overlap (curves) of the first 50 normal modes with the conformational transition. The bars depict how much of the movement individual modes capture. *lmc*ENM largely outperforms the baseline ENM and the reference ENM variants (color coding is the same as in panel B). The vertical dotted line marks the cumulative mode overlaps reached with the first ten low-frequency modes. (B) Residue fluctuations along the first ten low-frequency modes scaled to fit the observed displacement magnitudes (filled gray curve) between the two conformations. The Pearson correlation coefficient is given in brackets behind the ENM labels.

Fig 10A shows the cumulative mode overlap of *lmc*ENM of the first 50 low-frequency normal modes compared to ENM (baseline) and *mc*ENM (theoretical maximum). The first ten *lmc*ENM-modes capture 89% of the functional transition. With the same number of modes, ENM explains only 29%, *mc*ENM 86% overlap. edENM (43%) and HCA (40%) slightly improve over the baseline ENM. Hence, *lmc*ENM substantially improves over the baseline and the reference ENMs even when considering up to 50 modes. *lmc*ENM even outperforms *mc*ENM (theoretical upper bound) w.r.t. to the first ten modes. This is surprising because *mc*ENM contains not only the removed false-positive predicted breaking contacts in *lmc*ENM but also lacks observed breaking contacts that have not been detected by *lmc*ENM. The reason is that the three most relevant *lmc*ENM-modes are spread among modes 1, 2, and 4, which account for translation and upwards swinging of the *α*-helix. The corresponding *mc*ENM-modes distribute among modes 1, 3, and 10. Thus, *lmc*ENM seems to capture the network topology around this helix slightly more accurate than *mc*ENM, maybe due to the missed breaking contacts between the shorter helix (red) and a larger helix (Fig M in S1 File). As a result *lmc*ENM-modes focus more on the movement of the large helix (blue). Nonetheless, both methods perform about the same when considering more than ten modes.

Fig 10B shows that the changed contact topology of *lmc*ENM also accounts for a much better match between predicted and observed residue fluctuations, in particular for the most flexible helix (*α*2-helix). The other ENM variants, including the baseline ENM, largely underestimate the flexibility of this helix. *lmc*ENM consistently improves over the other ENM variants also w.r.t. all other measures (Table C in S2 File).

15S-LOX1 is not the only protein, where *lmc*ENM is more accurate than *mc*ENM. Overall, eight of the 90 proteins in our data set are better captured by *lmc*ENM than by *mc*ENM (see Table C in S2 File). This further underlines the potential of our method to explain functional transitions that could not be captured otherwise.

#### i. Feature importance

The features used to differentiate breaking from maintained contacts cover a broad range of properties. In particular, they characterize the physicochemical, structural and graph-based properties of the local neighborhood of a contact and its associated secondary structure elements. Hence, the question arises, which features contribute the most to a correct classification. Sorting features by the weights obtained after training a classifier on all features is one of the fastest methods for feature selection [98]. This works well for linear SVMs but not for kernel-SVMs, as the one used in our method, which are non-linear. Therefore, we tested the performance of a linear SVM on our problem as implemented in scikit-learn [65, 99] by Leave-One-Out Cross-Validation. Although the kernel-SVM classifies more accurate and also yields better performance for the corresponding *lmc*ENM, the difference is rather small (Tables Q and R in S1 File). Thus, the feature weights of the linear SVM should be a reasonable indicator of feature importance in our classification problem.

**Fig 11:**
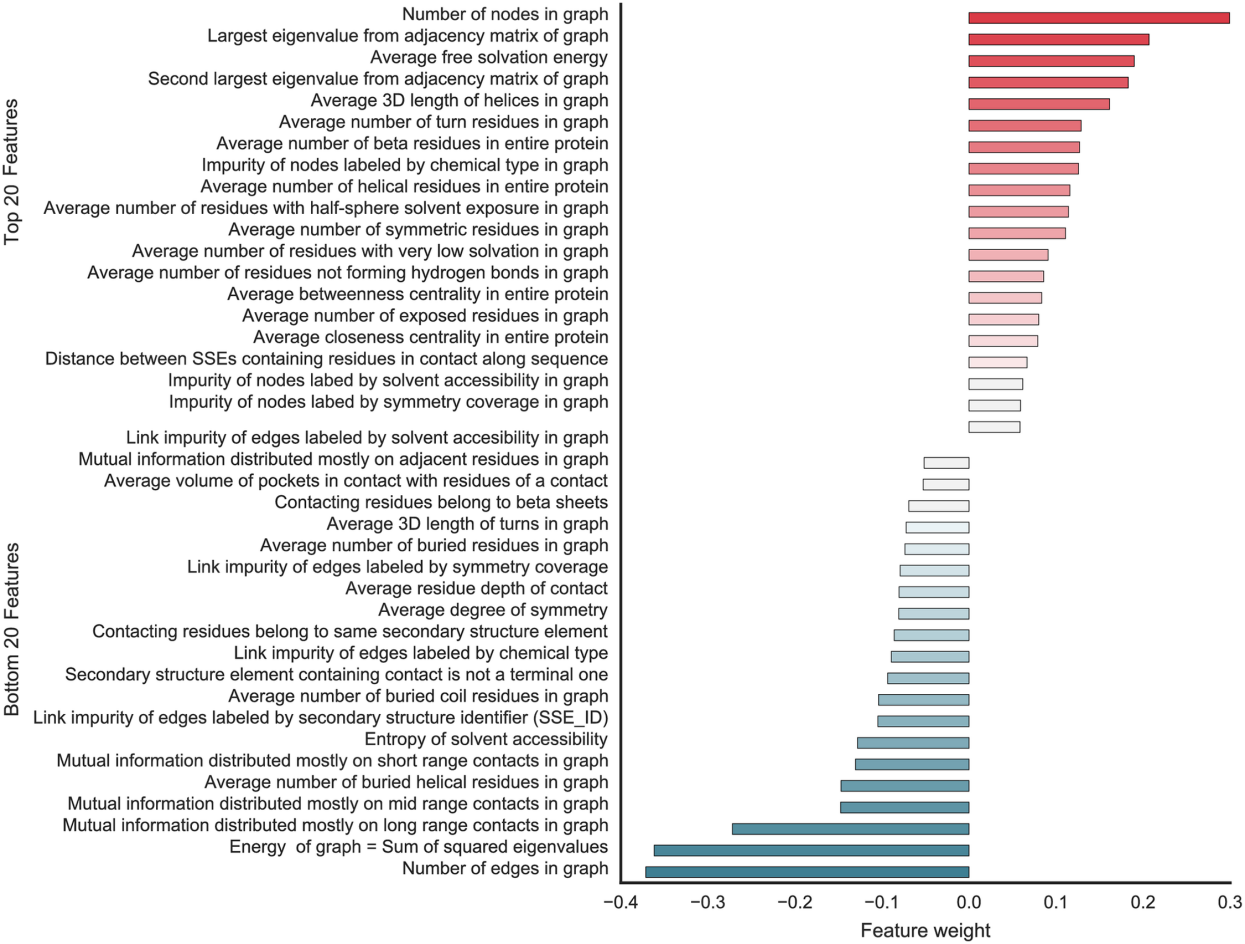
Top20 and Bottom20 features ranked by weight of the linearSVM. Features with largest weight are most important to classify breaking contacts, while features with minimum negative weight serve to identify maintained contacts. The graph refers to the neighborhood graph defining the local context of a single contact (see Methods). Features characterizing the different properties of this graph seem to dominate the classification.

Fig 11 shows the 20 features with largest (top) and lowest (bottom) weights. Positive weights contribute to identify breaking contacts, whereas negative features help to classify maintained contacts. The magnitude of the weights indicate the importance of the feature. The majority of selected features characterizes topology, spectrum, or label statistics of the neighborhood graph capturing the local context of an individual contact (see Tables C-I and Text B in S1 File for detailed feature description).

In particular, the number of nodes and number of edges in the neighborhood graph are on opposite extremes of the importance spectrum. If the local neighborhood is relatively large but weakly connected, the contact is more likely to break. In contrast, contacts in densely constrained regions are more probable to be maintained. The latter is also supported by the strong negatively weighted energy of the graph, which is usually higher for larger graphs due to their higher dimensionality of the adjacency matrix [100]. The large positive weight of largest and second largest eigenvalue may be interpreted in terms of their gap [101]. Taken individually, they provide not much information, however their gap may hold relevant information about the graph connectivity. Although we did not include this gap as explicit feature, the SVM classifier may have exposed an implicit relation between both pointing towards breaking contacts. Also, high degree of solvent accessibility and exposure indicate breaking contacts, especially when the impurity degree in the local context is higher. Further, long helices (3D length), a larger amount of turn residues, low amount of hydrogen bonds in the neighborhood, as well as a larger sequential distance of the secondary structure elements holding the contact seem to promote its breaking.

On contrary, maintained contacts seem to populate rather buried neighborhoods (number of buried (helical/coil) residues, entropy of solvent accessibility, residue depth) with high degree of sequence conservation (mutual information distribution). In fact, Liu et al. [96] have shown that there is a strong link between sequence conservation and intrinsic deformability for enzymes. Although some sequence correlations may be irrelevant for protein dynamics, certain amino acids involved in substrate recognition tend to be both, more mobile, while also coevolve more often. This points towards a breaking contact, whereas high sequence conservation rather characterizes maintained contacts. A high degree of symmetry leads to enhanced structural stability (maintained contacts) in the symmetric parts, while weakly attached parts are more likely to move to facilitate a functional transition. The outer membrane transporter, FecA, presented in the case study above exemplifies the effect of stable symmetric core allowing motion within the barrel as well as at the entrances. The average 3D length of turns intuitively measures the extension of a turn. Largely extended turns or coils are restricted in their mobility due to stronger interactions with the neighborhood along their full length. Being in contact with pockets of larger volume also seems to be associated with maintained contacts. Contact with a pocket is established if at least one of the contacting residues touches the surface of one of the alpha spheres characterizing the pocket’s shape as determined by fPocket [102]. A possible explanation could be that large pockets may tend to maintain their shape and hence the contact topology. Breaking contacts are more likely to be found at the pocket entrance to accommodate for ligand binding.

One might argue that several of our feature are captured by other approaches. For instance, residue depth or solvent exposure of a contact are implicitly modeled by its embedding into a highly or weakly constrained part of an ENM, respectively. Also the influence of contact order, secondary structure type, and hydrogen bonding have been used to refine ENMs [29, 45, 47], for instance. However, Fig 11 reveals that only the topmost feature as well as the three bottommost features are clearly separated from the other features in terms of their weight/importance, whereas the importance of the other features shows a much smaller spread. In fact, the ranking and weight of these features slightly varies for the different motion categories (Table G in S2 File of the supporting information). This has two implications: First, rather a combination of several features instead of a few individual ones may better explain the motions a protein can perform. Second, depending on the protein this specific feature/property combination may also vary. Both effects may be difficult to capture implicitly by modeling specific interactions, such as hydrogen bonds or disulfide bridges.

Overall, the strongest of our features seems to be the graph-based encoding of the local contact environment itself. With the presented feature set it holds valuable information about protein dynamics and can easily be extended by additional features. Yet, to improve the classifier by removing irrelevant features and to gain deeper understanding about features driving protein motion more advanced methods for features selection such as recursive feature elimination (SVM-RFE) [98] could be used. They also provide information about the importance and interplay of feature groups as opposed to their individual importance, which is analyzed with the above approach. Nonetheless, the interplay of features is partially captured by the contact’s neighborhood graph, which combines individual properties of its environment into aggregated features.

#### j. Limitations of *lmc*ENM

Given that *lmc*ENM preserves the simplicity of the classical ENM the computational costs to analyze the network’s deformability are comparable. However, *lmc*ENM requires additional computation to predict the breaking contacts needed to adjust its contact network, which largely depend on the protein’s size. Feature generation ranges from a few minutes for small proteins (> 100 residues) up to half an hour for our largest protein, FecA, with 647 residues on a single CPU. The prediction step is much faster taking seconds for small proteins up to six minutes for FecA. Nonetheless, the gain in accuracy of *lmc*ENM should compensate for these additional computational costs. Only training of the classifier is computationally more intense. But in principle, it has to be done only once and runs parallelized. A web service to run *lmc*ENM for single-chain proteins is currently in preparation.

**Fig 12:**
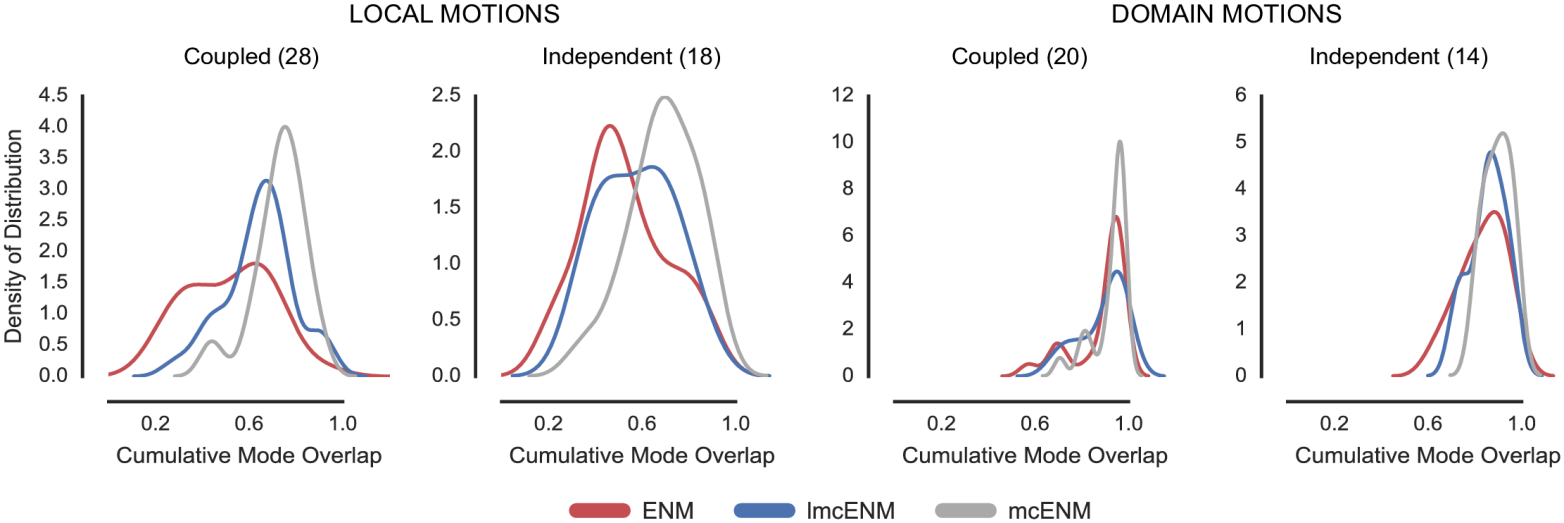
Distribution of *lmc*ENM-, *mc*ENM-, and ENM-accuracy considering the subset of local and domain motions (80 proteins). While *lmc*ENM closely resembles the accuracy distribution of *mc*ENM (theoretical upper bound) for proteins with coupled local motions and domain motions, it only slightly improves in accuracy for proteins with independent local motions. Nonetheless, *mc*ENM clearly demonstrates that also the latter type of motions can be captured with high accuracy with a refined contact topology.

Furthermore, the effectiveness of *lmc*ENM is currently mostly focused on proteins with local motions *coupled* to ligand binding, as shown in Fig 12. It reaches about two-third of the theoretical maximum accuracy achieved by *mc*ENM (see also Table N in S1 File). For proteins with independent local motions, *lmc*ENM is able to capture about half of them better than ENM, whereas the other half reaches only small if any improvement over ENM (baseline). Also, domain movers cannot benefit from *lmc*ENM to the extent as local movers, mostly due to the removal of too many predicted breaking contacts. Yet, our results clearly demonstrate that ENMs are able to capture previously poorly explained localized functional transitions. This further underlines the potential of our approach to further expand the range of motion types to be accurately modeled by elastic network models.

#### k. Potential applications of *lmc*ENM

Above we showed that *lmc*ENM alleviates a major shortcoming of ENMs being less suited to capture localized functional transitions with low degree of collectivity. By removing predicted breaking contacts, *lmc*ENM substantially improves the prediction accuracy for proteins performing local function-related movements. As a result, *lmc*ENM largely increases the chances that a protein’s motion is accurately modeled no matter if it performs a local or domain motion, thereby expanding the practical relevance of ENMs. In the following we will discuss some potential applications of ENMs that could benefit from using *lmc*ENM.

A logical first step would be to apply *lmc*ENM in the context of protein ligand docking. The ability of ENM to capture collective protein motions with only a few modes allows to narrow down the accessible deformation space of the unbound conformation. Hence, conformational sampling in this reduced space not only requires less computation, but also increases the chances to sample good candidate conformations for the actual docking. However, Dietzen et al. [39] showed that in small-protein docking conformational ensembles generated by sampling along ENM-modes often yield no improvement. The major obstacle seems to be that standard ENMs fail to capture the localized movements associated with ligand binding by the first few low-frequency modes. Although usually a few modes (less than ten) suffice to explain local transitions they are often spread among higher frequencies [27]. This makes it difficult to decide how many modes should be included to accurately sample the relevant deformation space. Our results show that *lmc*ENM effectively reduces the essential deformation space for localized functional transitions in most cases. Thus, it would be interesting to see whether a subset of for instance the first 20 low-frequency modes of *lmc*ENM would improve small-molecule docking. In addition, *lmc*ENM may also be helpful for the most difficult cases involving induced-fit movements that are triggered by the presence of a ligand. Training a SVM classifier specifically on such protein pairs may help to shift the most relevant *lmc*ENM-modes toward lower frequencies. This would alleviate the problem of identifying the relevant modes for a specific ligand because they already reside in the low-frequency mode spectrum.

For the same reasons, *lmc*ENM could also provide more accurate guidance for more fine-grained conformational sampling, especially for larger proteins performing localized functional transitions. Gur et al. [103], for instance, sample candidate structures mainly focusing on the space spanned by the first few low-frequency modes. These candidate structures serve as starting points for MD runs that generate conformations with full atomic detail. This multi-scale sampling can be used, for instance, to explore the conformational space accessible to the unbound conformation or to predict transition pathways between two end points of a function-related movement. Alternatively, ENM-modes have also been used to guide robotics-based sampling methods [33] to explore the conformational space with reduced computational costs. The quality of the guidance obviously depends on the accuracy of the predicted lowest-frequency modes. *lmc*ENM offers a way to improve this guidance for proteins exhibiting localized functional transitions that are difficult to capture by standard ENMs.

The predicted breaking contacts to construct *lmc*ENM may also be useful when constructing multi-scale ENMs, such as RCNMA [58], to predict motions of large proteins or complexes at a coarse-grained scale. While the occurrence of predicted breaking contacts reveals parts of the network requiring higher resolution, their absence indicates parts that could be further simplified. This would help to analyze only relevant parts of the protein and their motions in more detail, thereby reducing computational demands. However, to explore this further we would first need to extend our SVM prediction framework to accept multi-chain proteins and optimize the feature generation part in our pipeline to reduce computation time of breaking contact prediction.

Another interesting application for *lmc*ENM would be in the context of sampling of pathways between end points of functional transitions with a two-state ENM such as proposed by Das et al. [104]. Based on the ENMs of the two endpoints they construct a combined potential that allows to transition from one state to the other via an low-energy path. Such methods obviously require the knowledge of start and end conformation of a functional transition. However, in case the actual target conformation is unknown, the predicted network of learned maintained contacts of *lmc*ENM could be used as an estimate of the coarse-grained representation of the target conformation. Nonetheless, the prediction accuracy of the current SVM classifier may need to be improved before attempting such an experiment.

## Conclusion

We presented a novel elastic network model based on learned maintained contacts (*lmc*ENM) that offers an attractive route towards overcoming an important limitation of ENMs. Elastic network models (ENMs) exploit the fact that a protein’s motions are largely encoded in its contact topology. While ENMs accurately explain functional transitions of proteins that are large-scale and collective, they fail to capture localized, uncorrelated ones. Hence, the movements predicted by an ENM may be wrong or misleading. *lmc*ENM overcomes this limitation by predicting contacts that break during the motion. To predict these contacts we developed a machine-learning based classifier that differentiates breaking from maintained contacts using the aforementioned additional information. Our approach is a first step towards a “deformation-invariant” contact topology to study protein motions of any type on a coarse-grained scale.

Our approach is based on two key insights: First, the ability of ENMs to capture function-related transitions critically depends on a contact topology that remains maintained throughout the movement. While this is naturally fulfilled for highly collective movements, localized functional transitions often cause substantial changes in the contact topologies between start and end conformation. We showed that ENMs can accurately capture these localized movements if *observed* breaking contacts are removed from their initial contact topology. But, to *predict* protein motions with ENMs we also need to *predict* these breaking contacts. Second, the additional information required to predict breaking contacts is hidden in the physicochemical characteristics of local parts of the protein structure. These characteristics capture how tightly different parts of the protein are bound to each other, how this affects their movements, and ultimately their contact topology.

We showed that *lmc*ENM predicts function-related protein motions more accurate than the classical, distance-cutoff based ENM and three other reference ENM variants. *lmc*ENM is particularly effective in capturing ligand-coupled localized functional transitions that remain largely unexplained by all reference ENMs. Furthermore, we showed that *lmc*ENM reduces the complexity of the deformation space relevant to capture function-related movements. This has also implications for subsequent applications, such as generating conformational ensembles for protein-ligand docking, which often involves localized, functional transitions. These applications utilize the deformation space spanned by the lowest-frequency modes as guidance. Hence, they may benefit from a lower dimensional space that reduces the computational costs for sampling.

Last, we presented further evidence that protein motion likely results from the interplay of a broader set of properties/features characterizing the mobility of local structural parts. We also believe that combining different information sources (e.g. conformational ensembles obtained by MD, NMR, X-ray, or other experimental methods) will make the identification of relevant properties even more robust and accurate than relying on a single source alone. With our presented approach we provide a novel, unified, and extendible way to examine, exploit and relate additional features captured by each of these information sources in order to further advance our understanding of protein motion.

## Supporting information

S1 File Used features, supplementary tables, and figures. Graphs for modeling physicochemical context, detailed informations about the features and their generation, tables and plots reporting supplementary performance overviews of ENM variants and the SVM, and supplementary figures.
(PDF)

S2 File Datasets, supplementary tables with raw performance data and most relevant features. Dataset of proteins used to obtain the theoretical upper bound (*mc*ENM), to train and test the SVM and to evaluate *lmc*ENM, raw SVM performance data, breaking contact statistics, raw performance data of ENM variants, dataset of conformational ensembles, Top20 and Bottom20 features weighted by Linear SVM.
(ZIP)
