# Supplementary material for: Elastic network model of learned maintained contacts to predict protein motion
Source: PLoS One. 2017 Aug 30;12(8):e0183889. doi: 10.1371/journal.pone.0183889 (PMC5576689; doi:10.1371/journal.pone.0183889)
Supplement: S1 File — Graphs for modeling physicochemical context, detailed informations about the features and their generation, tables and plots reporting supplementary performance overviews of ENM variants and the SVM, and supplementary figures. (PDF) [file pone.0183889.s001.pdf]

# Elastic Network Model of Learned Maintained Contacts to Predict Protein Motion
# Supplementary Material

## Contents


**A Graphs for modeling physicochemical context** **3**
1.1 Node labels . . . . . . . . . . 3
1.2 Edge labels . . . . . . . . . . 5

**B Features listing and implementation details** **5**
2.1 Pairwise residue features . . . . . . . . . . 6
2.2 Graph features . . . . . . . . . . 9
2.2.1 Node label statistics . . . . . . . . . . 10
2.2.2 Edge label statistics . . . . . . . . . . 10
2.3 Whole protein features . . . . . . . . . . 10
2.4 External Software Used to Generate Features . . . . . . . . . . 11

**C Supplementary tables** **17**

**D Supplementary figures** **21**

## List of Figures


A Accuracy of *mc*ENM compared to ENM using additional metrics on LMC_all data set grouped by motion type . . . 21
B Accuracy of *mc*ENM w.r.t. maximum mode overlap related measures compared to baseline ENM on LMC_all data set grouped by motion type . . . 22
C Observed breaking contacts in contact topology of house dust mite allergen Der f. . . . 23
D Effect of breaking contact selection strategies on *lmc*ENM accuracy grouped by protein motion type . . . 24
E Sensitivity analysis of *lmc*ENM-selection cutoff (topN percent) for the eight proteins, where *lmc*ENM drops by more than 5% in accuracy compared to ENM (baseline) . . . 24
F Example protein (2dh3B), where *lmc*ENM performance significantly drops below ENM (baseline) . . . 25
G Accuracy of *lmc*ENM compared to reference ENM variants using additional metrics on LMC_all data set grouped by motion type . . . 26
H Accuracy of *lmc*ENM w.r.t. maximum mode overlap related measures compared to reference ENM variants on LMC_all data set grouped by motion type . . . 27
I Scatter plot of cutoff distance against protein length . . . 28
J Ability of *lmc*ENM to capture structural flexibility of conformational ensembles compared to ENM (baseline), *mc*ENM (theoretical upper bound) and three other ENM variants on subset of 35 proteins having at least 10 conformational states . . . 29
K Outer membrane transporter FecA: False positive predicted breaking contacts 30
L Contact networks for outer membrane transporter FecA based on the optimal extension threshold determined for this protein only . . . 31
M Observed and predicted breaking contacts of Arachidonate 15-Lipoxygenase (side view) . . . 32

# A Graphs for modeling physicochemical context

To model the local contact environment we use the graph-based encoding developed in previous work from our group [1]. Nodes in the contact graph refer to residues, which are connected by edges if they are in contact. Although Schneider et al. [1] aim to predict contacts for protein sequences, they used labels for nodes and edges of the contact graph that are quite general. We therefore reuse and extend the labels that are also relevant to derive characteristics of contact graphs in our context. Furthermore, we added novel labels specifically focusing on our problem domain. Tables A and B) summarize the node and edge labels and mark which labels are reused, extended, or novel. For convenience, we also recap the explanations of the reused and extended labels introduced in [1].

## 1.1 Node labels

Table A: Summary of node labels

| Node label | Possible labels |
| --- | --- |
| Chemical type[a] | Discrete value |
| Secondary structure[a] | Discrete value |
| Solvent accessibility[a] | Discrete value |
| Free solvation energy[a] | Continuous value |
| Secondary structure length[a] | Discrete value |
| Secondary structure 3D length[a] | Continuous value |
| Secondary structure buried[a] | Continuous value |
| Secondary structure exposed[a] | Continuous value |
| Hydrogen bonding[b] | Discrete value |
| Distance to the centroid[a] | Continuous value |
| Sequence conservation[a] | Continuous value |
| Sequence neighborhood conservation[a] | Continuous value |
| Secondary structure unique ID | Continuous value |
| Part of symmetric element | Discrete value |
| Depth | Continuous value |

[a]Reused node label from Schneider et al. [1].
[b]Extended node label from Schneider et al. [1].

**Chemical type:** A residue can be non-polar, polar, acidic, or basic.

**Secondary structure:** A residue can be part of a helix, sheet, turn, or coil.

**Solvent accessibility:** distinguishes between buried and exposed residues. The former have a relative solvent accessibility (calculated by POPS [2]) $\leq 25\%$, while the latter are above this cutoff.

**Free solvation energy:** calculated by POPS [2].

**Secondary structure length:** Length of the secondary structure element associated with the residue, measured along the sequence.

**Secondary structure 3D length:** Three-dimensional distance (in Å) between first and last residue of the secondary structure element calculated between their $C_\alpha$ atoms.

**Secondary structure buried:** Specifies how buried the residue's secondary structure element is based on its average number of buried residues.

**Secondary structure exposed:** Specifies how exposed the residue's secondary structure element is based on its average number of exposed residues.

**Hydrogen bonding:** Residue can be donor, acceptor or not part of a hydrogen bond.

**Distance to the centroid:** Three-dimensional distance (in Å) between the $C_\alpha$ atom of the residue and the centroid of the protein structure.

**Sequence conservation:** Specifies the degree of sequence conservation of the residue obtained from a multiple-sequence alignment (based on [**?**, 3]).

**Sequence neighborhood conservation:** Specifies the degree of sequence conservation within the local neighborhood of the residue up to three sequence positions away ($i-3$, $i-2$ $i-1$, $i+3$, $i+2$ $i+1$) as in [**?**, 3].

**Secondary structure unique ID:** Unique identifier of the secondary structure element the residue belongs to.

**Part of symmetric segment:** Specifies whether the residue is part of symmetric segment in protein (calculated by SymD [4]).

**Structural depth:** Specifies the depth of the residue w.r.t. the solvent accessible surface by averaging the distance of its atoms to the surface vertices (calculated with BioPython [5]).

**Half Sphere Exposure:** Specifies the degree of exposure of a residue by counting the contacts within the upper and lower half-sphere (default radius 12Å) around the residue's $C_\alpha$ atom. The sphere is cut into two halves by a plane centered at the $C_\alpha$-atom, which is perpendicular to the vector between the $C_\alpha$- and a pseudo-$C_\alpha$-atom. (calculated with BioPython [5] based on [6]).

### 1.2 Edge labels

Table B: Summary of edge labels

| Edge label | Possible labels |
|---|---|
| 3D distance[a] | Continuous value |
| Mutual information[a] | Continuous value |

[a]Reused node label from [1].

**3D distance:** Specifies how far away the two residues of the contact are in 3D by calculating the distance between their $C_\alpha$.

**Mutual information:** The mutual information in the multiple-sequence alignment between the two residue positions of the contact.

## B Features listing and implementation details

The dynamic behavior of contacts depends on their immediate local context as well as their embedding into the overall arrangement of local structural parts. The physicochemical interactions between these parts control their movement with respect to each other, which ultimately influences the contact topology between them. We use a set of features to characterize the properties of the local neighborhood of a contact as well as its associated secondary structure elements. Feature can be a single real-value input or encode categorical properties by a set of binary values. Unless stated otherwise, a categorical feature with $k$ states is encoded by an $k$-dimensional binary input vector. The individual features are concatenated into a single vector that serves as input for the SVM to differentiate breaking from maintained contacts. This feature vector has a total length of 170 input values.

We designed features specific to our problem domain, as well as reuse or extend features used in previous work from our group [1]. The features fall into eight categories: Pairwise, graph topology, graph spectrum, single node, node label statistics, edge label statistics and

whole protein features. In the following we introduce in detail the features in each category and mark all reused or extended features respectively.

### 2.1 Pairwise residue features

Pairwise features encode properties of an individual contact. As contacts seldom change their distance in isolation, many of the pairwise features are defined on their associated secondary structure element(s) (SSEs). Table C lists the individual features together with their number of input values in the feature vector. For all features that are not self-explanatory a detailed description of the feature and its generation is given in the text.

**Distance between secondary structures elements along protein chain (SSE):** Distance between relative index positions of SSEs associated with residue $i$ and $j$ along the protein chain (1 inputs).

**SSE-contact type:** Contacts can be within a SSE (intra-SSE) or between (inter-SSE). While the dynamic behavior of intra-SSE contacts mostly depends on the intrinsic flexibility of the SSE, inter-SSE contacts are influenced by the strength of the interface (1 input). Intrinsic SSE-flexibility or SSE-interface strength are characterized by the following features.

**SSE-interface contact position:** Position of an inter-SSE contact within the SSE-interface. Contacts located at the border of the interface have a higher probability to break than more central ones. Contacts with at least one residue in the border region (outer 10% of interface length measured in residue position) are encoded by [1,0], core contacts by [0,1] (2 inputs).

**SSE-interface hydrogen bonding:** Fraction of hydrogen bonds in SSE-interface relative to total number of hydrogen bonds in the structure. 0 for intra-SSE contact. (1 input)

**SSE-interface density:** Density of SSE-interface indicating the degree of connectedness of the two SSEs. Strongly connected SSE-interfaces are likely to be maintained. The interface of two SSEs can be represented as a bipartite graph, where the two disjunct node sets refer to the interface-residues of the two SSEs. The density of the SSE-interface is then calculated as actual number of contacts between these two node sets divided by the maximal possible number of contacts of a fully connected bipartite graph. 0 for intra-SSE contact. (1 input).

**SSE-interface degree:** Averaged number of interface connections of residues $i$ and $j$, respectively. Indicates how much the contacting residues contribute to the SSE-interface strength. 0 for intra-SSE contact. (1 input).

**SSE-interface redundancy:** Fraction of other contacting residues of residues $i$ or $j$ in SSE-interface that would remain in contact in a one-mode projection even if $i$ or $j$ would be removed [7]. This could be viewed as a measure of the importance of individual SSE-interface residues to maintain the connectivity of the interface. 0 for intra-SSE contact (1 input).

**SSE-interface balanced:** Equal number of residues participating in SSE-interface on both sides or not. Indicates whether the SSE-interface has "exposed" contacts with rather long distance (imbalanced SSE-interface). Due to a lower degree of connectivity in their neighborhood such contacts are more likely to break than shorter, highly constrained ones. 0 for intra-SSE contact. (1 input).

**Contact between terminal SSEs:** Terminal regions of the protein chain often possess more flexibility. This feature captures if the contacting residues belong to one of the two terminal secondary structure element along the protein chain (5 binary inputs in total). We distinguish five cases: If the contact is an intra-SSE contact, the SSE can be terminal or not (2 binary inputs). If the contact is between different SSEs, both SSEs can be terminal, only one, or none (3 binary inputs).

**SSE-intra hydrogen bonding:** Fraction of hydrogen bonds within SSE relative to total number of hydrogen bonds of the protein. Hydrogen bonds increase the stability of a SSE-interface. 0 for inter-SSE contact (1 input).

**SSE-intra degree:** Averaged intra-SSE degree of residue $i$ and $j$, i.e. the number of contacts within SSE of each residue. $\alpha$-helices, for instance, have a lower probability to unfold compared to loops that have fewer internal constraints. 0 for intra-SSE contact (1 input).

**Contact with highest ranked pocket:** This feature captures if both residues, $i$ and $j$, are "in-contact" with the highest ranked pocket, or only one of the residues, or none. (3 inputs). Location and properties of pockets used to generate all pocket-related features are calculated with FPocket [8]. FPocket reports detected pockets ranked by a probability score to be the functional-active binding site. Contacts around the binding pocket have higher propensity to change. They may be involved in movements to accomodate the ligand in the binding site or to shield it from the solvent.

**Contact with a pocket:** This feature captures if both residues, $i$ and $j$, are "in-contact" with any detected pocket, or only one of the residues, or none. (3 inputs). Being in touch with any pocket, not necessarily the binding pocket, increases the changes for secondary structure elements to move into this "free space". Such movements may be required to

propagate, for instance, allosteric signals and may result in changes of the local contact topology.

**Exposure to pocket:** Sum of atom contacts to closest pocket of residues $i$ and $j$. A high number of atom contacts indicates that the residue extends into the pocket, i.e. has a high degree of exposure into the pocket (1 input).

**Polarity of pocket:** Average polarity of pocket(s) in contact with residues $i$ and $j$ (1 input). The polarity of a pocket is a measure for its hydrophilicity (calculated with FPocket [8]).

**Hydrophobicity of pocket:** Average hydrophobicity of pocket(s) in contact with residues $i$ and $j$ (1 input). This feature measures the degree of hydrophobicity of a pocket (in contrast to the polarity above, calculated with FPocket [8]).

**Druggability score of pocket:** Average druggability score of pocket(s) in contact with residues $i$ and $j$ (1 input). This score estimates the probability of a pocket to bind small drug like molecules (calculated with FPocket [8])

**Volume of pocket:** Average volume of pocket(s) in contact with residues $i$ and $j$ (1 input, calculated with FPocket [8]).

**Side chain contact:** Captures if contact is also a side chain contact. Two residues are in side chain contact if at least one pair of heavy side chain atoms is within 4.5Ådistance to each other (1 input).

**Contact depth:** Captures the depth of a contact based on the normalized structural depth of its residues (4 inputs). The residue depth is binned into four states: really deep (lower than 0.25), deep (between 0.25 and 0.5), exposed (between 0.5 and 0.75) and very exposed (larger than 0.75). If the depth class of the contacting residues differs, the class of the deeper residue determines the contact depth. Deeply buried contacts are more likely to be maintained than contacts close to the surface.

**Residue depth difference:** Measures the binned difference in structural depth of the contacting residues. The normalized depth difference bins are: $\Delta_d epth < 0.25, 0.25 <= \Delta_d epth < 0.5, 0.5 <= \Delta_d epth < 0.75, \Delta_d epth > 0.75$. Depending on the actual contact depth, also the difference in depth of the contacting residues may influence the contact's dynamic behavior. A large depth difference increases the chances that a contact may break (4 inputs).

**SSE-symmetry coverage:** (Average) fraction of residues being part of symmetric structural parts of the SSE(s) associated with the contact (see section 1.1). Symmetric parts of a protein structure often stabilize the overall fold. For instance, $\beta$-barrels, $\beta$-sheets, or mixed $\alpha$-$\beta$-barrels (TIM-barrels), are strongly stabilized by hydrogen bonds. Hence, contacts between SSEs involved in symmetric arrangements are likely to be maintained. (1 input).

**Contact symmetry coverage:** Captures if both residues of a contact are part of symmetric segment (see section 1.1), only one or none (3 inputs).

**Distance to symmetry plane:** This feature captures the distance of the contacting residues to the symmetry plane (6 binary inputs in total). Both residues can be far apart (normalized distance $\geq 0.7$) from the symmetry plane either on the positive or negative side (2 binary inputs). Or both residues are on either positive or negative side, but only one is far away from the symmetry plane (2 binary inputs). Or one residue is on the positive and the other one on the negative side (1 binary input). If no symmetry plane exists all except the last input are 0. Contacts closer to the core of a symmetric part of the protein structure may benefit more from its higher stability. Hence, they are likely to be maintained. However, contacts close to the border of a symmetric part, such as a $\beta$-barrel, may have a higher chance to be involved in the functional activity of the protein. We discuss an example of a highly symmetric membrane protein in detail in case study II (see Results and Discussion in main document).

**Secondary structure:** The secondary structures types (helix, sheet, turn or coil) of the protein are obtained with STRIDE [9] (10 inputs).

**Solvent accessibility:** The solvent accessibility of residues is classified into solvent exposed or buried (see section 1.1) (3 inputs).

**Hydrogen bonding:** Residues of a contact can be bonded by an hydrogen bond, or be donor or acceptor of another hydrogen bond, or not involved in hydrogen bonding. 1.1) (3 inputs).

**Mutual information:** The mutual information in the multiple-sequence alignment between positions $i$ and $j$ (1 input).

## 2.2 Graph features

Our work is based on the assumption that breaking contacts and maintained contacts show differences in the properties of their local neighborhood. To specify these differences

we re-use the graph-topology (Tab. D), graph spectrum (Tab. E), and single node features (Tab. F) from [1]. These topological features and node/edge label statistics characterize the properties of the local context of a contact defined by its immediate neighborhood graph (see Methods in main document) and help us to distinguish breaking from maintained contacts.

For convenience we list the re-used features in the following tables (Tables D, E,and F). Detailed explanations of these features can be found in the original publication [1].

#### 2.2.1 Node label statistics

Node label statistics, listed in Table G, capture the frequency of different node labels in the graph.

**Average degree of symmetry:** The degree of symmetry for a single node is the fraction of neighbor nodes that belong to symmetric segments. Average degree of symmetry is the average over all nodes in the graph (1 input).

**Neighborhood impurity degree:** Normalized number of neighbor nodes with different labels in the graph. Schneider et al. [1] evaluated the neighborhood impurity degree for the node labels chemical type, secondary structure, solvent accessibility. We extend this node label list by unique secondary structure identifier (SSE_ID), symmetry coverage, large positive distance to symmetry plane, large negative distance to symmetry plane (7 inputs).

#### 2.2.2 Edge label statistics

Edge label statistics, listed in Table H, capture the frequency of different edge labels in the graph.

**Link impurity:** Normalized number of edges between nodes with different labels in the graph. Schneider et al. [1] evaluated the link impurity for the edge labels chemical type, secondary structure, solvent accessibility. We extend this edge label list by unique secondary structure identifier (SSE_ID), symmetry coverage, large positive distance to symmetry plane, large negative distance to symmetry plane (7 inputs).

**Mutual information distribution:** Fraction of edges between nodes with different ranges of sequence separation (adjacent, 2-6, 7-11, 12-23, >24), yielding a 5-bin distribution of the mutual information of the graph. (5 inputs).

### 2.3 Whole protein features

These features characterize global properties of the whole protein (Table I). We reused one feature from [1] in this category, which is marked.

### 2.4 External Software Used to Generate Features

We use FPocket [8] to compute location and properties of pockets and cavities. Symmetric segments and symmetry axes are calculated with SymD [4]. Residue depth is computed with Biopython [5]. In addition, we rely on software used by Schneider et al. [1] to generate the features re-used or extended by us. For completeness we also list these software packages. POPS [2] is used to calculate solvent accessibility and free solvation energies. Secondary structure types and hydrogen bonds are assigned based on STRIDE [9]. Features capturing sequence conservation are calculated based on methods in [3]. The Python library NetworkX [10] is used to generate graphs as well as topological and spectral graph features. Last, to classify contact changes we rely on the SVM library of scikit-learn [11] that internally builds on LIBSVM [12].

Table C: Pairwise features between contacting residues $i$ and $j$ or their associated secondary structure elements (SSEs)

| Feature | Description | Number of inputs |
|---|---|---|
| Distance between SSEs along protein chain | Relative distance between position of SSEs along protein chain | 1 |
| Centroid distance between SSEs | 3D-distance between centroids of SSEs | 1 |
| SSE-contact type | Contact within same SSE (intra-SSE) or between different SSEs (inter-SSE) | 1 |
| SSE-interface hydrogen bonding | See text | 1 |
| SSE-interface contact position | See text | 4[a] |
| SSE-interface density | See text | 1 |
| SSE-interface balanced | See text | 1 |
| SSE-interface degree | See text | 1 |
| SSE-interface redundancy | See text | 1 |
| SSE-intra hydrogen bonding | See text | 1 |
| SSE-intra degree | See text | 1 |
| Contact with highest ranked pocket | See text | 3[a] |
| Contact with a pocket | See text | 3[a] |
| Exposure to pocket | See text | 1 |
| Polarity of pocket | See text | 1 |
| Hydrophobicity of pocket | See text | 1 |
| Druggability score of pocket | See text | 1 |
| Volume of pocket | See text | 1 |
| Side chain contact | See text | 1 |
| Contact depth | See text | 4[a] |
| Residue depth difference | See text | 4[a] |
| SSE-symmetry coverage | See text | 1 |
| Contact symmetry coverage | See text | 3 |
| Distance to symmetry plane | See text | 6 |
| Contact between terminal SSEs | See text | 5 |
| Secondary structure type[b] | Secondary structure of the contacting residues: helix, sheet, turn, coil | 10[a] |
| Hydrogen bonding[c] | See text | 3[a] |
| Mutual information[b] | Sequence mutual information | 1 |
| Total inputs | | 63 |

[a]Binary inputs
[b]Reused from Schneider et al. [1].
[c]Extended from Schneider et al. [1].

Table D: Graph topology features[a]

| Feature | Description | Number of inputs |
|---|---|---|
| Number of nodes | Number of nodes in the graph | 1 |
| Number of edges | Number of edges in the graph | 1 |
| Average degree centrality | Average number of node neighbors indicating packing density of graph. | 1 |
| Average closeness centrality | Average reciprocal distance of each node to all other nodes in the graph. | 1 |
| Average betweenness centrality | Average number of shortest paths passing through each node of the graph indicating the degree of influence of individual nodes onto the network. | 1 |
| Average eccentricity | Average maximum distance between each node and all other nodes in the graph. | 1 |
| Graph radius | Smallest eccentricity in the graph. | 1 |
| Graph diameter | Largest eccentricity in the graph. | 1 |
| Number of end points | Number of nodes with only one neighbor. | 1 |
| Average clustering coefficient | Average number of actual neighbors divided by possible neighbors of each node in the graph measuring the degree of transitivity in the network. | 1 |
| Total inputs | | 10 |

[a]Reused from Schneider et al. [1].

Table E: Graph spectrum features derived from the adjacency matrix[a]

| Feature | Description | Number of inputs |
|---|---|---|
| Largest eigenvalue | Largest eigenvalue | 1 |
| Second largest eigenvalue | Second largest eigenvalue | 1 |
| Number of different eigenvalues | Number of different eigenvalues | 1 |
| Sum of eigenvalues | Trace of the adjacency matrix | 1 |
| Energy | Sum of squared eigenvalues | 1 |
| Total inputs | | 5 |

[a]Reused from Schneider et al. [1].

Table F: Single node features[a]

| Feature | Description | Number of inputs |
|---|---|---|
| Degree centrality | Number of node neighbors of node $i$ and $j$. | 2 |
| Closeness centrality | Reciprocal average distance from nodes $i$ and $j$ to all other nodes in the graph. | 2 |
| Betweenness centrality | Number of shortest paths that pass through nodes $i$ and $j$. | 2 |
| Sequence separation from N/C-terminus | Distance in sequence position of $i$ to N-terminus and $j$ to C-terminus | 2 |
| Sequence conservation | Conservation of residue position of $i$ and $j$ in multiple sequence alignment. | 2 |
| Sequence neighborhood conservation | Conservation of neighboring residues of $i$ and $j$ in multiple-sequence alignment. | 2 |
| Total inputs | | 12 |

[a]Reused from Schneider et al. [1].

Table G: Node label statistics

| Feature | Description | Number of inputs |
|---|---|---|
| Symmetry coverage | Average number of nodes covered by symmetry | 1 |
| Average degree of symmetry | Average fraction of node neighbors that are covered by symmetry for all nodes in the graph | 1 |
| Residue depth | Average residue depth in graph | 1 |
| Residue depth distribution | 5-bin distribution of residue depth in graph | 5 |
| Average half-sphere exposure | Average lower/upper half-sphere exposure in graph | 2 |
| Neighborhood impurity degree[b] | Average number of neighbors with different labels | 7 |
| Hydrogen bonding[b] | Average numbers of nodes that act as donor, acceptor or do not form hydrogen bonds | 3 |
| Label entropy[a] | Entropy of the different labels, calculated for chemical type, secondary structure, and solvent accessibility. | 3 |
| Chemical type[a] | Number of polar, non-polar, acidic, basic labels | 4 |
| Secondary structure distribution[a] | Number of nodes with helix, sheet, turn, coil labels | 4 |
| Secondary structure length[a] | Average length of secondary structure element in amino acids | 4 |
| Secondary structure 3D length[a] | Average 3D length of secondary structure element | 4 |
| Secondary structure buried[a] | Average number of buried residues per ss_type (helix, sheet, turn, coil) | 4 |
| Secondary structure exposed[a] | Average number of exposed residues per ss_type (helix, sheet, turn, coil) | 4 |
| Solvent accessibility[a] | Average number of exposed/buried nodes | 2 |
| Average solvation energy[a] | Average free solvation energy | 1 |
| Solvation energy distribution[a] | 4-bin distribution of free solvation energy | 4 |
| Distance to centroid[a] | Average distance of nodes to the centroid | 1 |
| Sequence conservation[a] | Average sequence conservation of nodes | 1 |
| Sequence neighborhood conservation[a] | Average sequence neighborhood conservation of nodes | 1 |
| Total inputs | | 57 |

[a]Reused from Schneider et al. [1].
[a]Extended from Schneider et al. [1].

Table H: Edge label statistics

| Feature | Description | Number of inputs |
|---|---|---|
| Link impurity[b] | Number of edges connecting two nodes with different labels | 7 |
| Mutual information distribution[a] | 5-bin distribution of mutual information | 5 |
| Cumulative mutual information[a] | Cumulative mutual information over all edges | 1 |
| Total inputs | | 13 |

[a]Reused from Schneider et al. [1].
[b]Extended from Schneider et al. [1].

Table I: Whole protein features

| Feature | Description | Number of inputs |
|---|---|---|
| Secondary structure composition[b] | Distribution of secondary structure types in protein (helix, sheet, turn, coil). | 4[a] |
| Connectivity class | Binned number of contacts of protein (<500, 501-1000, 1001-2000,2001-3000 >3000). | 5[a] |
| Symmetry coverage | Normalized number of residues in symmetric segments. | 1 |
| Total inputs | | 10 |

[a]Binary inputs
[b]Reused from Schneider et al. [1].

# C Supplementary tables

Table J: Performance of $\text{ANM}_{minDeg4}$ at cutoff value $r_c$ ranging between 8 and 18Å measured by the cumulative mode overlaps of the first ten low-frequency modes evaluated on the LMC_all data set (90 proteins). Cutoffs 9Å and 10Å yield best median overlap. However, only $\text{ANM10}_{minDeg4}$ fulfills the criterion of six zero eigenvalues making it the better choice as baseline for our approach.

| $r_c$ (Å) | 8[a] | 9[b] | 10 | 11 | 12 | 13 | 14 | 15 | 16 | 17 | 18 |
|---|---|---|---|---|---|---|---|---|---|---|---|
| median | 0.670 | 0.685 | 0.685 | 0.677 | 0.673 | 0.675 | 0.665 | 0.660 | 0.647 | 0.624 | 0.615 |
| mean | 0.661 | 0.665 | 0.665 | 0.667 | 0.664 | 0.660 | 0.651 | 0.646 | 0.639 | 0.631 | 0.622 |

[a]10 cases with more than the six trivial zero eigenvalues
[b]3 cases with more than the six trivial zero eigenvalues

Table K: Performance of *mc*ENM at different extension thresholds $e_c$ used to distinguish breaking from maintained contacts. Contacts that extend their distance by less than $e_c$ percent of their initial distance are considered maintained, otherwise breaking. *mc*ENM is based on the best performing $\text{ANM}_{minDeg4}$ at cutoff value 10Å (see Table S10). The performance is measured by the cumulative mode overlaps of the first ten low-frequency modes evaluated on the LMC_all data set (90 proteins). Extension thresholds between 5% and 9% reach similar performance, with slightly better median at threshold 9%. Removing too many breaking contacts as for threshold 5% may lead to instable networks. Hence, we chose extension threshold 9% to build *mc*ENM in this study.

| $e_c$ (%) | 5[a] | 7 | 9 | 11 | 13 | 15 | 17 | 19 | 21 | 23 | 25 |
|---|---|---|---|---|---|---|---|---|---|---|---|
| median | 0.819 | 0.819 | 0.820 | 0.815 | 0.807 | 0.801 | 0.795 | 0.790 | 0.777 | 0.778 | 0.779 |
| mean | 0.804 | 0.800 | 0.799 | 0.793 | 0.787 | 0.781 | 0.774 | 0.769 | 0.767 | 0.763 | 0.762 |

[a]1 case with more than the six trivial zero eigenvalues

Table L: Performance overview of ENM, *mc*ENM, and *mfc*ENM on the LMC_all data set (90 proteins). The performance is measured by the cumulative mode overlap of the first ten low-frequency modes (CO). *mc*ENM relies on observed maintained contacts, while *mfc*ENM additionally contains observed forming contacts. For each ENM variant the table reports the median and mean CO of the proteins grouped by their motion types (coupled/independent local motions (CLM and ILM), coupled/independent domain motions (CDM and IDM), burying ligand motions (BLM), and other types of motions (OTM)). The number of proteins in each category is given in brackets after the motion labels. The last row reports the mean and median CO for all proteins.

| Motion types | ENM | *mc*ENM | *mfc*ENM |
|---|---|---|---|
| CLM (27) | 0.53/0.52 | 0.74/0.73 | 0.31/0.32 |
| ILM (18) | 0.48/0.53 | 0.68/0.69 | 0.39/0.39 |
| IDM (14) | 0.85/0.83 | 0.90/0.90 | 0.24/0.24 |
| CDM (21) | 0.94/0.88 | 0.96/0.92 | 0.20/0.24 |
| BLM (4) | 0.70/0.70 | 0.82/0.84 | 0.47/0.52 |
| OTM (6) | 0.62/0.61 | 0.82/0.76 | 0.21/0.26 |
| ALL (90) | 0.69/0.66 | 0.82/0.80 | 0.28/0.31 |

Table M: SVM performance overview of the top16% predicted breaking contacts on the full LMC_all data set (90 proteins). Different performance measures are reported for the proteins grouped by their motion types (coupled/independent local motions (CLM and ILM), coupled/independent domain motions (CDM and IDM), burying ligand motions (BLM), and other types of motions (OTM)). Median and mean values are reported for each measure. The number of proteins in each category is given in brackets after the motion labels. The last row reports the average values for all proteins.

| | Precision | Coverage | AUC[a] |
|---|---|---|---|
| Motion types | | | |
| CLM (27) | 0.24/0.27 | 0.41/0.44 | 0.62/0.61 |
| ILM (18) | 0.22/0.25 | 0.40/0.41 | 0.56/0.58 |
| CDM (21) | 0.18/0.18 | 0.43/0.44 | 0.64/0.62 |
| IDM (14) | 0.15/0.16 | 0.39/0.37 | 0.62/0.63 |
| BLM (4) | 0.13/0.19 | 0.32/0.30 | 0.49/0.51 |
| OTM (9) | 0.20/0.30 | 0.25/0.28 | 0.55/0.55 |
| ALL (90) | 0.19/0.23 | 0.41/0.41 | 0.61/0.60 |

[a] Area under curve (AUC) of receiver operator characteristic (ROC)

Table N: Performance overview of *lmc*ENM compared to baseline ENM and *mc*ENM (theoretical upper bound), as well as three other reference ENM variants on the LMC_all data set (90 proteins). The performance is measured by the cumulative mode overlap (CO) of the first ten low-frequency modes. *lmc*ENM consists of the learned maintained contacts after removing the top16% predicted breaking contacts. For each ENM variant the median and mean CO is reported of the proteins grouped by their motion types (coupled/independent local motions (CLM and ILM), coupled/independent domain motions (CDM and IDM), burying ligand motions (BLM), and other types of motions (OTM)). The number of proteins in each category is given in brackets after the motion labels. The last row reports the average values for all proteins. While the other ENM variants perform about the same, *lmc*ENM clearly improves in capturing localized functional transitions over their common baseline, thereby reaching almost half of the improvement made by *mc*ENM.

| Motion types | ENM | OFC-ENM | edENM | HCA | *lmc*ENM | *mc*ENM |
|---|---|---|---|---|---|---|
| CLM (28) | 0.53/0.52 | 0.57/0.54 | 0.55/0.53 | 0.58/0.54 | 0.66/0.64 | 0.74/0.73 |
| ILM (18) | 0.48/0.53 | 0.49/0.53 | 0.46/0.51 | 0.48/0.53 | 0.58/0.58 | 0.68/0.69 |
| CDM (20) | 0.94/0.88 | 0.94/0.88 | 0.94/0.89 | 0.94/0.88 | 0.94/0.89 | 0.96/0.92 |
| IDM (14) | 0.85/0.83 | 0.85/0.83 | 0.87/0.85 | 0.85/0.85 | 0.85/0.86 | 0.90/0.90 |
| BLM (4) | 0.75/0.75 | 0.77/0.75 | 0.81/0.80 | 0.78/0.77 | 0.75/0.76 | 0.87/0.88 |
| OTM (6) | 0.62/0.61 | 0.63/0.62 | 0.64/0.60 | 0.64/0.60 | 0.65/0.60 | 0.82/0.76 |
| ALL (90) | 0.69/0.66 | 0.67/0.67 | 0.68/0.67 | 0.68/0.68 | 0.73/0.72 | 0.82/0.80 |

Table O: Proteins, where *lmc*ENM drops by more than 5% in cumulative overlap of the first ten low-frequency modes compared to ENM (baseline). For each ENM variant the difference w.r.t. ENM is shown in percent (Δ). In some cases also other ENM variants perform worse than ENM albeit to a lesser extent.

| Unbound | ENM | Δ (%) *lmc*ENM | Δ (%) OFC-ENM | Δ (%) edENM | Δ (%) HCA | Δ (%) *mc*ENM | Motion label | RMSD | #Residues | Scop class | #Domains |
|---|---|---|---|---|---|---|---|---|---|---|---|
| 1dx9C | 0.340 | -10.20 | 2.90 | 0.50 | 4.70 | 26.40 | OTM | 1.690 | 168 | a/b | 1 |
| 2dh3B | 0.439 | -10.80 | -0.70 | -2.80 | -0.50 | 9.70 | ILM | 1.730 | 416 | None | 2 |
| 1gohA | 0.467 | -11.20 | -0.20 | -3.60 | -2.70 | 26.80 | ILM | 1.890 | 639 | all beta | 1 |
| 1a8dA | 0.523 | -8.30 | 2.90 | -6.60 | -1.10 | 19.90 | ILM | 1.870 | 451 | all beta | 2 |
| 2jepB | 0.635 | -17.70 | 3.10 | -11.30 | -0.10 | 17.30 | ILM | 1.140 | 359 | None | 1 |
| 1kp9A | 0.739 | -8.70 | 0.70 | -4.10 | -1.80 | 5.60 | CLM | 4.010 | 270 | a/b | 1 |
| 2v8iA | 0.910 | -5.60 | 0.50 | -0.40 | -0.20 | 5.90 | IDM | 2.000 | 535 | None | 3 |
| 1lfhA | 0.931 | -10.30 | 0.10 | -1.80 | -0.50 | 1.90 | CDM | 6.510 | 691 | a/b | 3 |

Table P: Best value of selection cutoff (topN percent) of *lmc*ENM and corresponding cumulative overlap of the first ten low-frequency modes for proteins, where *lmc*ENM performs significantly worse than ENM (baseline). For ease of comparison, also the cumulative overlap of ENM and *lmc*ENM based on the chosen selection cutoff of top16% as well as corresponding precision and coverage of the SVM is shown. Fig F shows how the cumulative overlap for these proteins evolves, when gradually removing more predicted breaking contacts.

| Unbound | ENM | *lmc*ENM | $CO10_{best}$ | Cutoff$_{best}$ (%) | SVM Precision | SVM Coverage |
|---|---|---|---|---|---|---|
| 1dx9C | 0.340 | 0.238 | 0.328 | 3 | 0.153 | 0.308 |
| 2dh3B | 0.439 | 0.331 | 0.384 | 4 | 0.218 | 0.300 |
| 1gohA | 0.467 | 0.355 | 0.469 | 1 | 0.101 | 0.185 |
| 1a8dA | 0.523 | 0.440 | 0.547 | 3 | 0.164 | 0.411 |
| 2jepB | 0.635 | 0.458 | 0.618 | 1 | 0.064 | 0.338 |
| 1kp9A | 0.739 | 0.652 | 0.738 | 1 | 0.434 | 0.429 |
| 2v8iA | 0.910 | 0.854 | 0.912 | 1 | 0.043 | 0.137 |
| 1lfhA | 0.931 | 0.828 | 0.932 | 4 | 0.108 | 0.289 |

Table Q: Performance of linear SVM (cost=100) and kernel SVM (cost=100, $\gamma$=0.00001) on the LMC_all data set (90 proteins). Performance is measured by precision and coverage of the $L/5$ contacts with highest SVM score, where $L$ refers to the length of the protein. The kernel SVM performs slightly better.

| | Precision | Coverage |
|---|---|---|
| linear SVM | 0.294 | 0.455 |
| kernel SVM | 0.307 | 0.470 |

Table R: Performance of *lmc*ENM based on linear SVM and *lmc*ENM based on kernel SVM (our presented approach) compared to ENM (baseline), and *mc*ENM (theoretical upper bound) on the LMC_all data set (90 proteins). Performance is measured by the cumulative mode overlap of the first ten low-frequency modes. Both *lmc*ENM-variants reach largest overlap when removing the top16% predicted breaking contacts. *lmc*ENM based on linear SVM performs slightly worse.

| | ENM | *lmc*ENM (kernel SVM) | *lmc*ENM (linear SVM) | *mc*ENM |
|---|---|---|---|---|
| Cumul. Mode Overlap (10) | 69/0.66 | 0.72/0.71 | 0.73/0.72 | 0.82/0.80 |

## D Supplementary figures

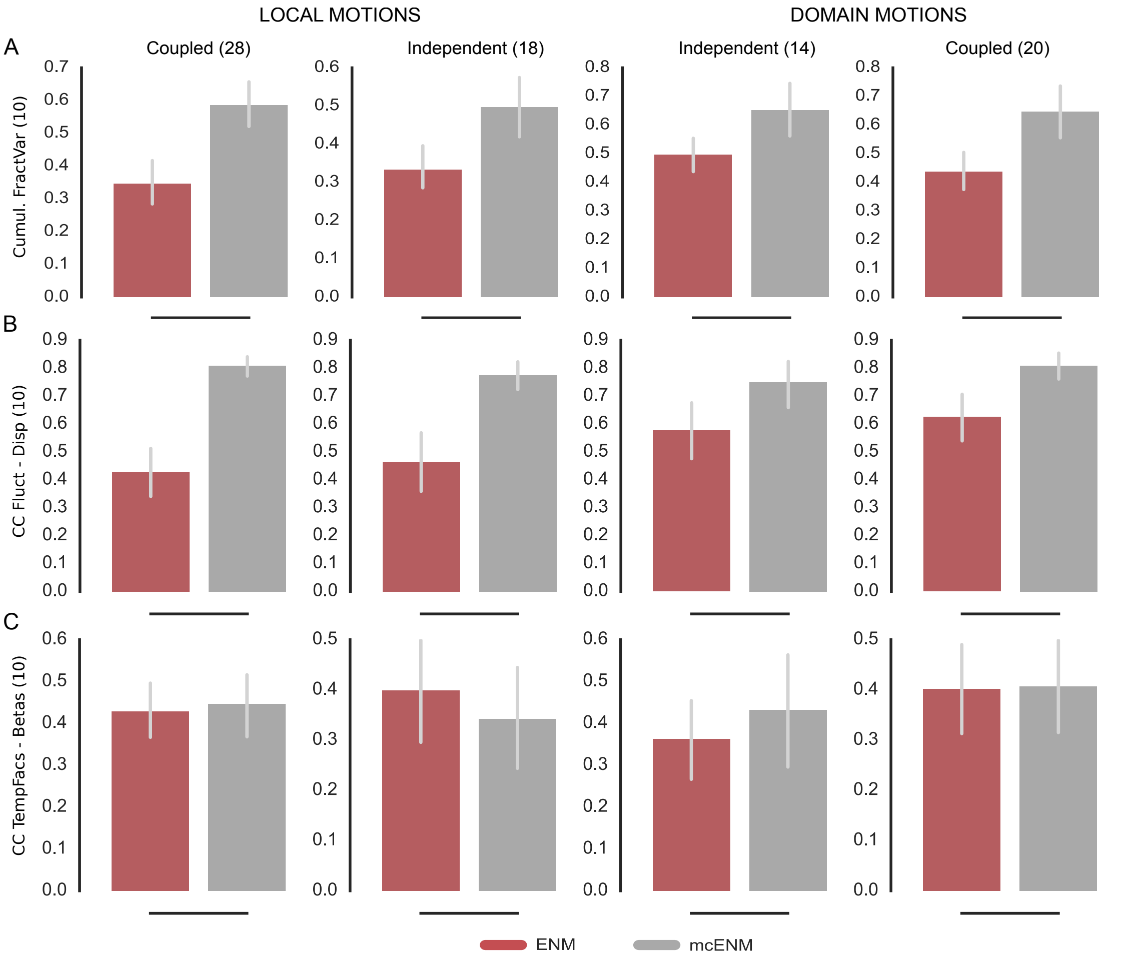


Figure A: **Accuracy of *mc*ENM compared to ENM using additional metrics on LMC_all data set grouped by motion type** (A) Cumulative Fraction of Variance (10 modes). (B) Correlation coefficient between predicted residue fluctuations and observed displacement magnitudes (10 modes). (C) Correlation coefficient between predicted Temperature factors and experimental Beta factors (10 modes). *mc*ENM consistently outperforms ENM considering the first two measures. The improvement is largest for proteins with localized functional transitions. Considering the similarity of temperature factor profiles *mc*ENM and ENM perform roughly the same.

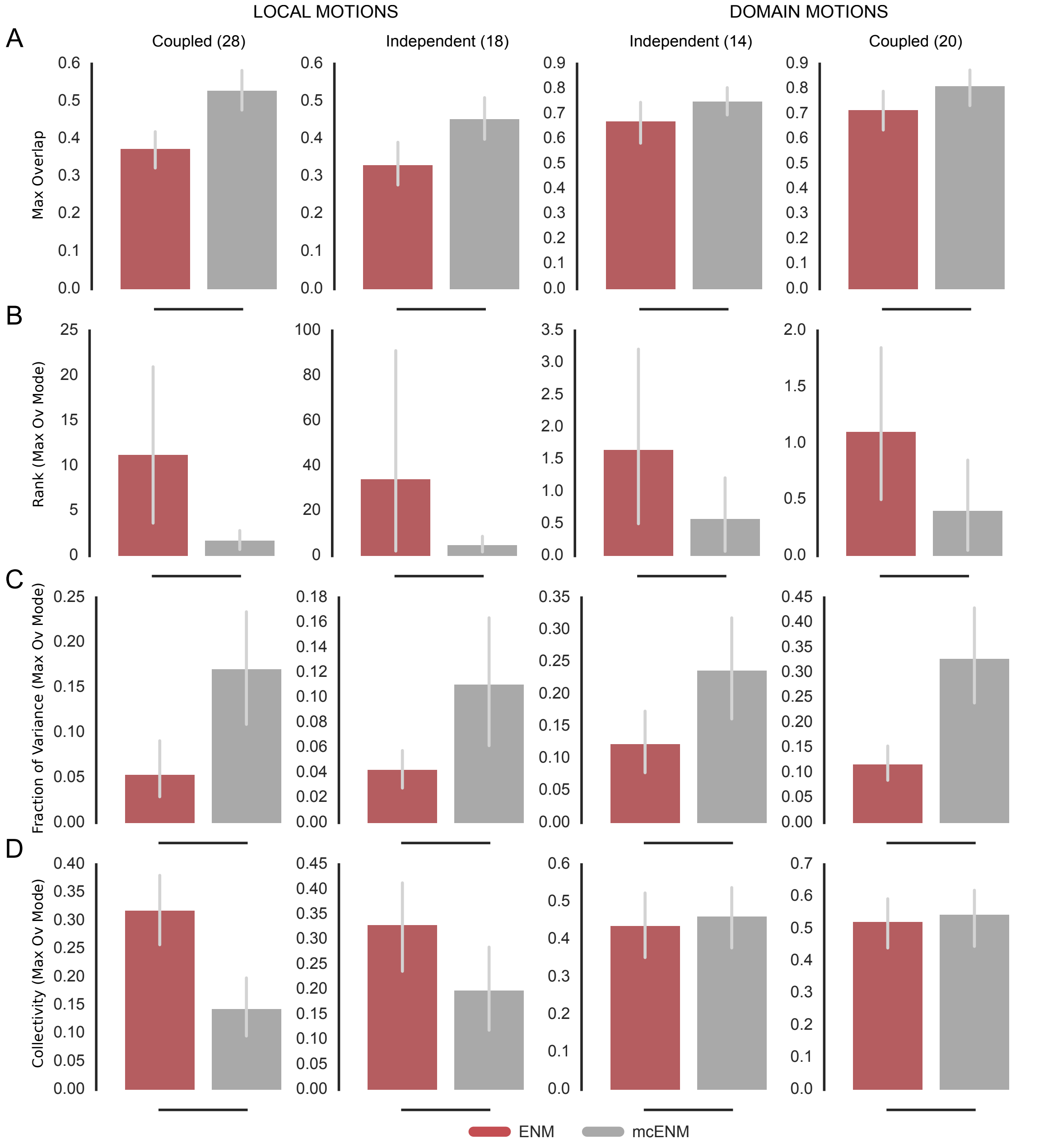


Figure B: **Accuracy of *mc*ENM w.r.t. maximum mode overlap related measures compared to baseline ENM on LMC_all data set grouped by motion type** (A) Maximum mode overlap of all modes. (B) Rank of best-overlapping mode. (C) Fraction of variance explained by best-overlapping mode. (D) Degree of collectivity of best-overlapping mode.

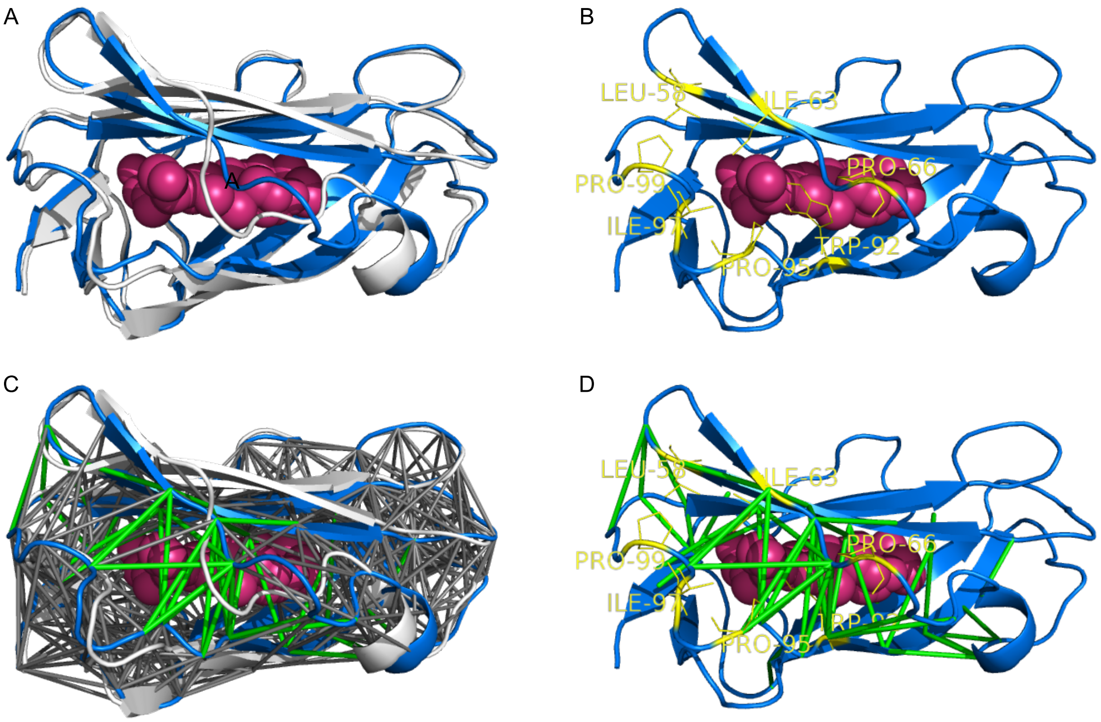


Figure C: **Observed breaking contacts in contact topology of house dust mite allergen Der f.** (A) Unbound and bound conformation (PDB_IDs: 2f08D, 1xwvB) colored blue and white, respectively. Ligand is shown as magenta spheres. (B) The ligand is proposed to enter the binding site via a narrow tunnel opening. The residues forming the tunnel opening are highlighted in yellow [13]. (C) Observed breaking (green) and maintained (gray) contacts networks. (D) Observed breaking contacts locate around the proposed tunnel opening.

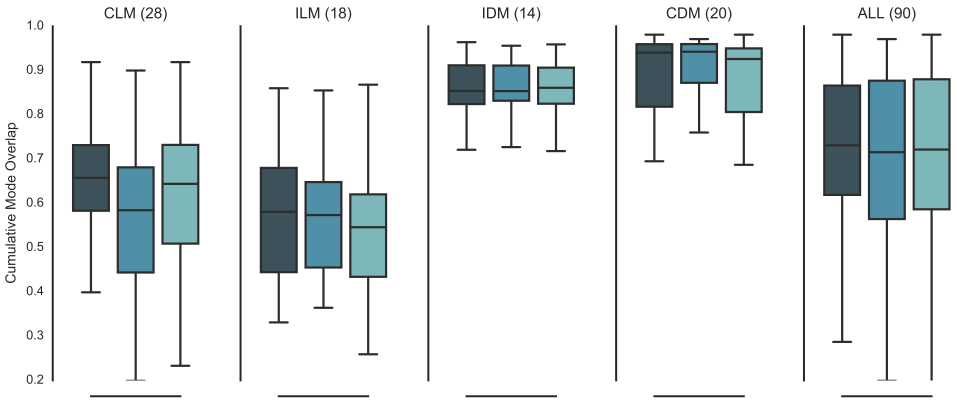


Figure D: **Effect of breaking contact selection strategies on *lmc*ENM accuracy grouped by protein motion type.** For each strategy we chose the cutoff value that maximizes the accuracy of *lmc*ENM averaged over all proteins in our data set (90). The panels show the reached *lmc*ENM-accuracy distribution for each strategy as box plot, where boxes show the quartiles of the data. The numbers above each box report the mean. The selection strategies reach similar performance with small advances for the relative cutoff strategy.

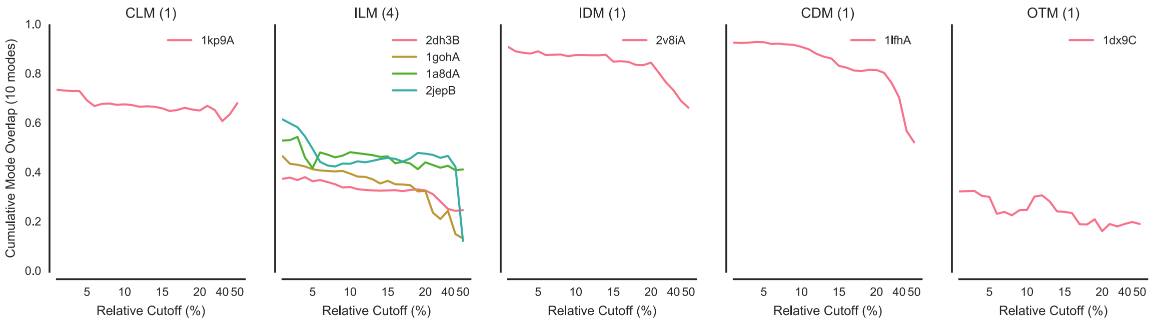


Figure E: **Sensitivity analysis of *lmc*ENM-selection cutoff (topN percent) for the eight proteins, where *lmc*ENM drops by more than 5% in accuracy compared to ENM (baseline).** Dependence of *lmc*ENM accuracy on removed topN% predicted breaking contacts ranked by decreasing SVM score for the eight proteins grouped by their motion type. The lines depict how *lmc*ENM-accuracy evolves for individual proteins when gradually removing more breaking contacts from their network. In all cases the accuracy drops almost starting from the beginning. The optimal topN percent cutoff for each protein is reported in Table S17 above.

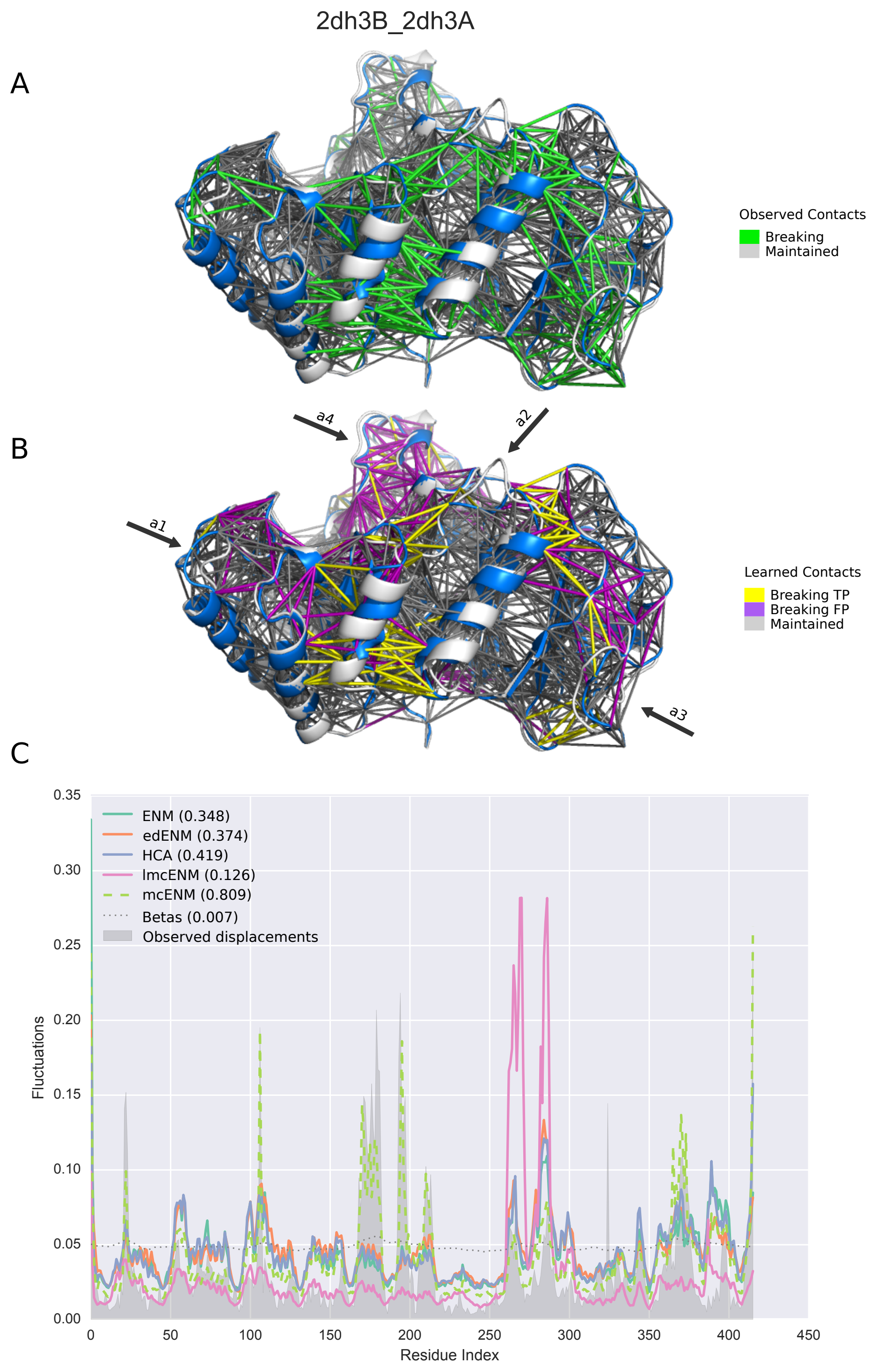


Figure F: **Example protein (2dh3B), where *lmc*ENM performance significantly drops below ENM (baseline).** (A) Observed breaking and maintained contact networks. Unbound and bound conformation colored blue and white, respectively. (B) Predicted true-positive (TP), false-positive (FP) breaking, and maintained networks. (C) Fluctuation profiles of all ENM variants scaled to observed displacements. The dark arrows point to parts, where *lmc*ENM substantially underestimates the flexibility between residues 106-109 (a1), 160-220 (a2: helix-loop-helix), 360-380 (a3) because relevant observed breaking contacts mostly constraining flexible loops have not been predicted. Between residues 260-290 (a4) it largely overestimates flexibility due to the removal of too many false-positives.

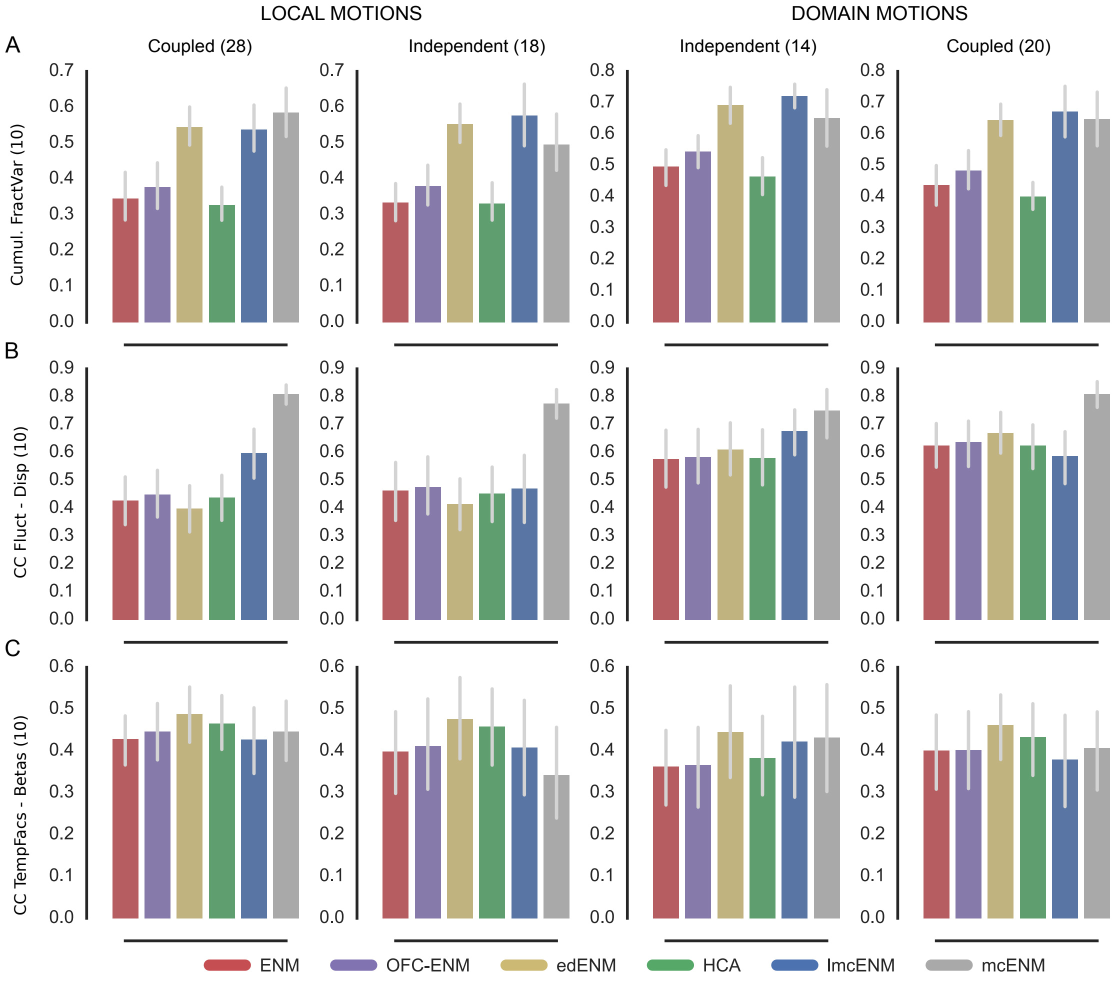


Figure G: **Accuracy of *lmc*ENM compared to reference ENM variants using additional metrics on LMC_all data set grouped by motion type** (A) Cumulative Fraction of Variance (10 modes). *lmc*ENM and edENM consistently capture by far largest amount of structural variance with the lowest frequency modes, with slight advances for *lmc*ENM except for coupled local motions. They perform as good or better than *mc*ENM (theoretical upper bound). (B) Correlation coefficient between predicted residue fluctuations and observed displacement magnitudes (10 modes). For coupled local and independent domain motions *lmc*ENM reaches largest agreement between predicted and observed fluctuation profiles. For the other two motion types *lmc*ENM performs as good as the other ENM variants or slightly worse. (C) Correlation coefficient between predicted Temperature factors and experimental Beta factors (10 modes). Considering the similarity of temperature factor profiles *lmc*ENM and ENM perform roughly the same. The largest agreement for all motion types is achieved by edENM.

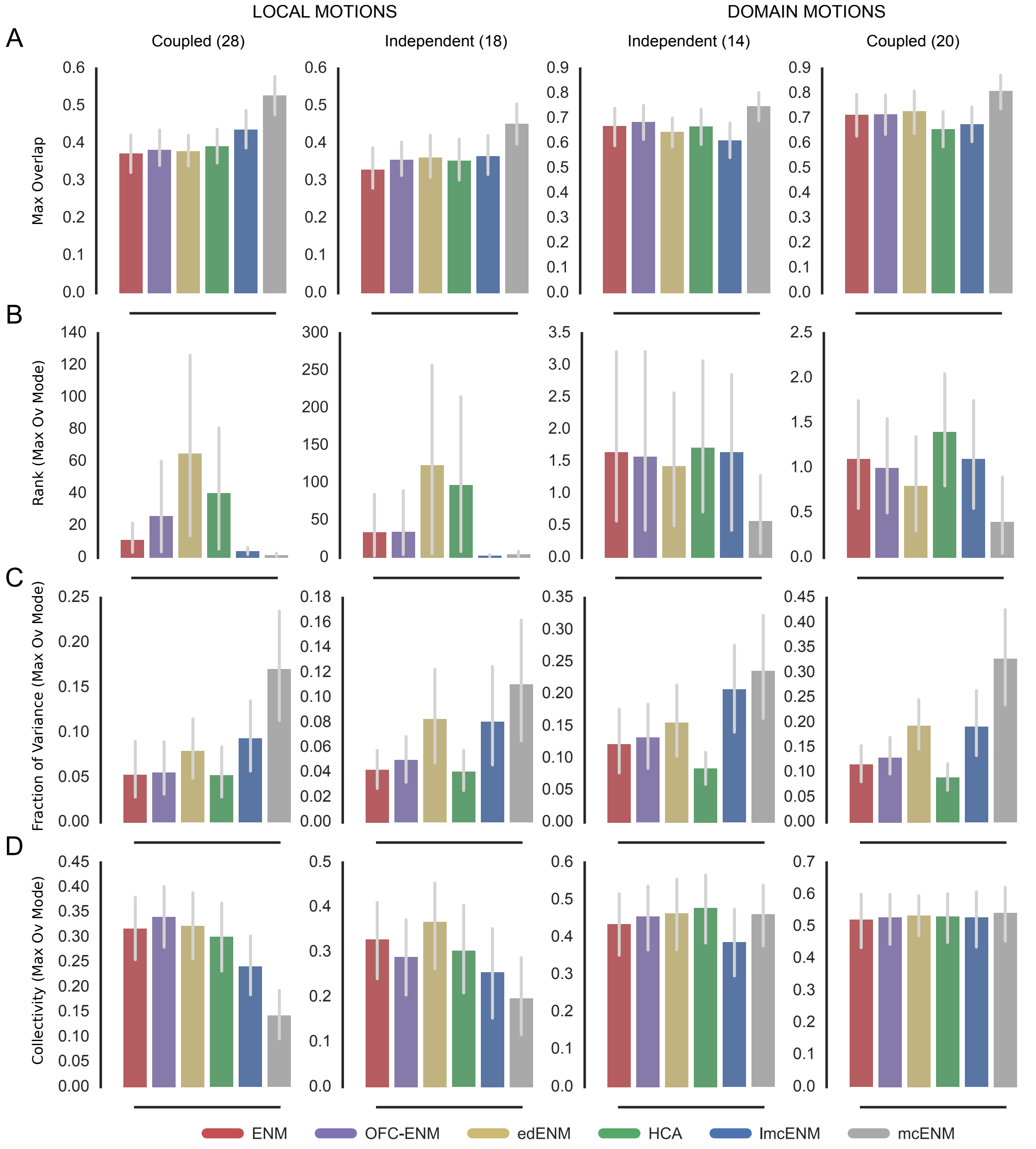


Figure H: **Accuracy of *lmc*ENM w.r.t. maximum mode overlap related measures compared to reference ENM variants on LMC_all data set grouped by motion type** (A) Maximum mode overlap of all modes. (B) Rank of best-overlapping mode. (C) Fraction of variance explained by best-overlapping mode. (D) Degree of collectivity of best-overlapping mode.

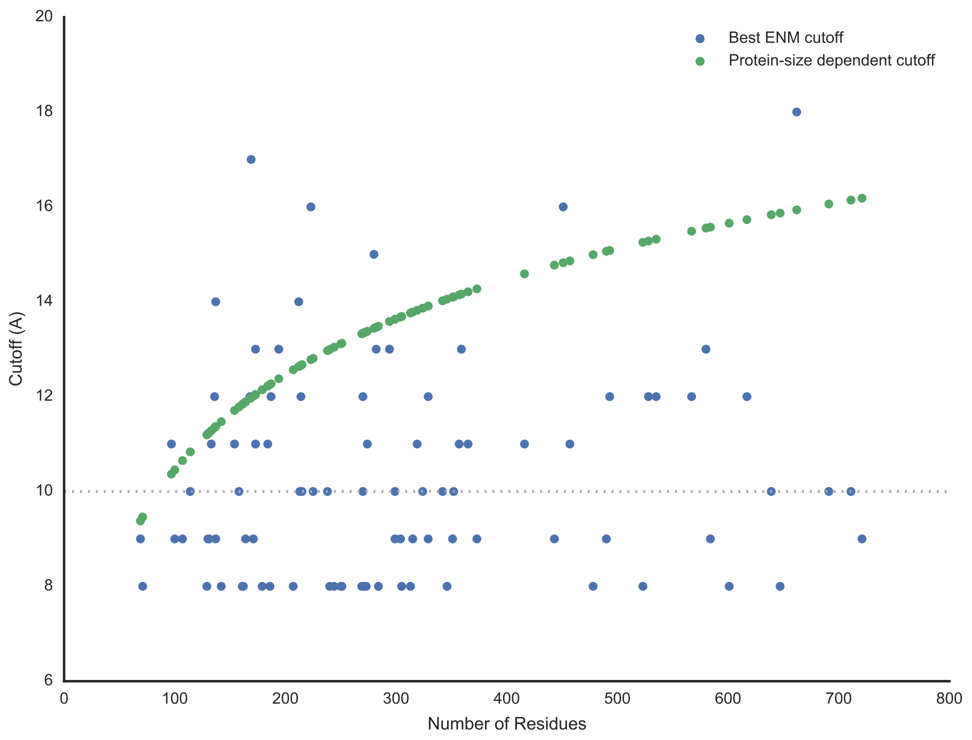


Figure I: **Scatter plot of cutoff distance against protein length.** In blue the best cutoff values of the baseline ENM are shown, which yield the largest cumulative mode overlap of the first ten low-frequency modes (in cutoff range 8-18A). In green a protein-size dependent cutoff is shown as proposed by Orellana et al.[14]. The dotted horizontal line indicates the median best ENM cutoff. In our set of proteins we find no correlation between optimum cutoff and protein size. The protein-size dependent cutoff largely over-constrains the network for most proteins in our data set.

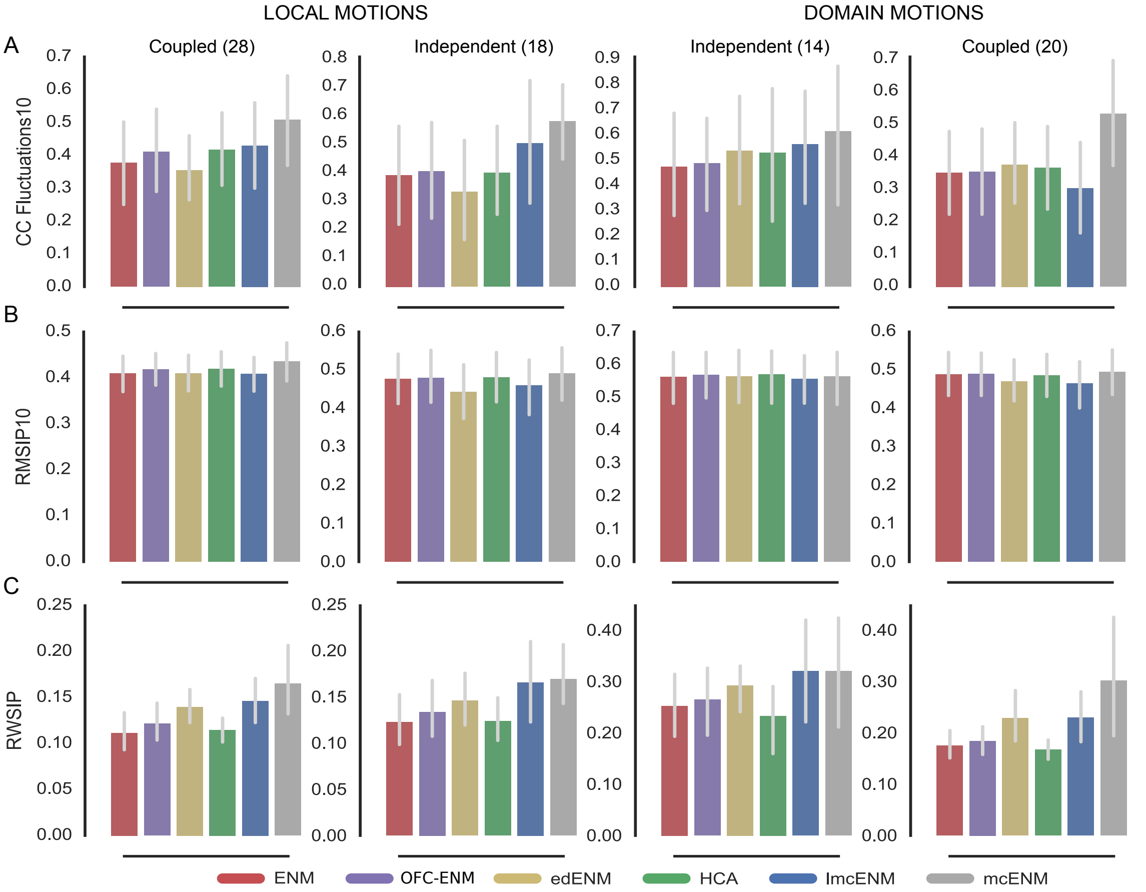


Figure J: **Ability of *lmc*ENM to capture structural flexibility of conformational ensembles compared to ENM (baseline), *mc*ENM (theoretical upper bound) and three other ENM variants on subset of 35 proteins having at least 10 conformational states**. The panels grouped by motion type show the similarity of fluctuation profiles (magnitudes) considering the first ten low-frequency modes (A), the subspace overlap (directions) of the same mode set (B), and the weighted overlap (directions and magnitudes) of both spaces (C). *lmc*ENM clearly outperforms the other ENM variants in the most robust weighted overlap, i.e. when taking into account motion directions and magnitudes captured by the full deformation spaces. Closest in performance is edENM, which performs slightly worse for coupled local motions and comparable for coupled domain motions.

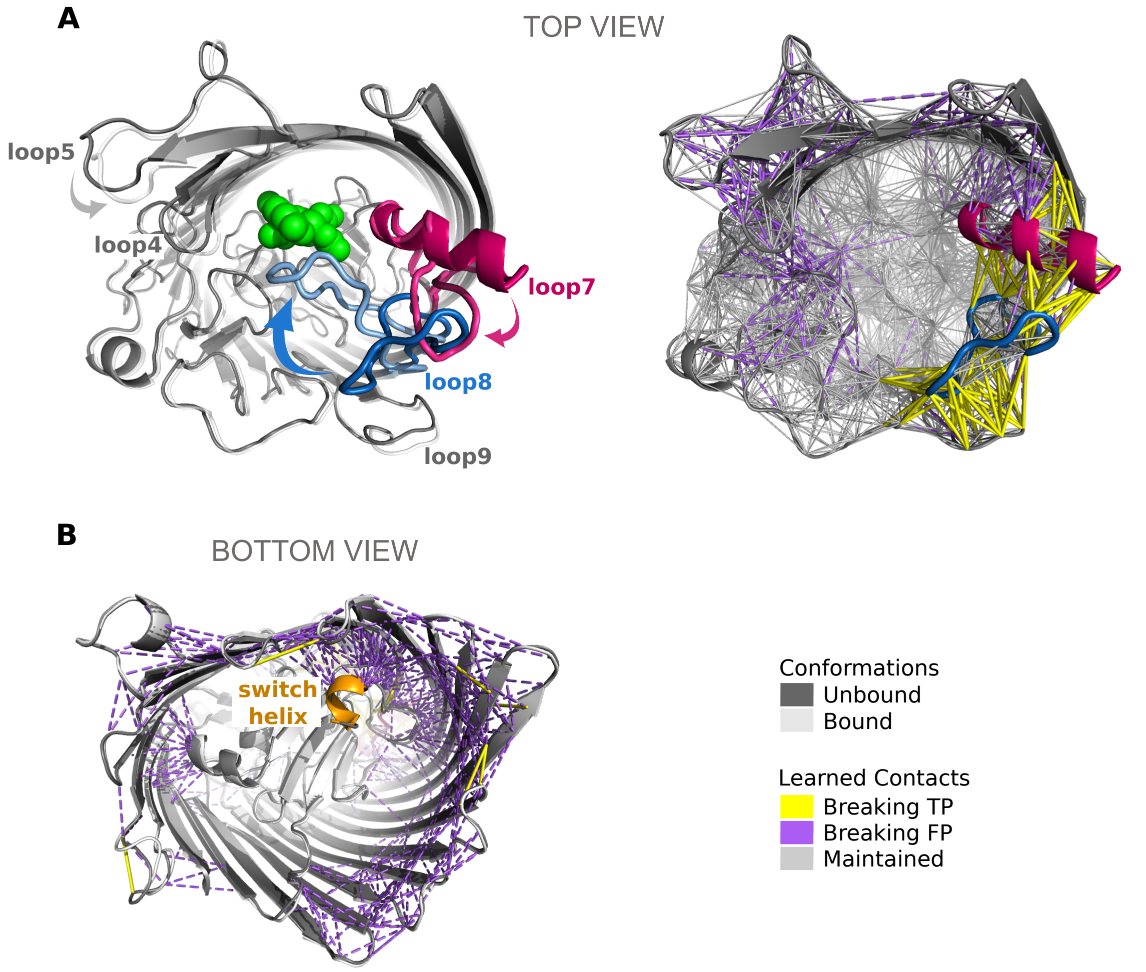


Figure K: **Outer membrane transporter FecA: False positive predicted breaking contacts**. (A) Top view of the transition between unbound and bound conformation (left) compared to the location of predicted breaking contacts (right). Loops 7 and 8 dominate the conformational change. Most of the predicted breaking contacts around these loops are true positives (TP). Some more breaking contacts are predicted at the opposite side of these loops, but considered false positive predictions (FP). (B) Bottom view of FecA in unbound and bound state. Only the learned breaking contacts are shown for clarity. Many false positive predicted breaking contacts locate around the switch helix (orange). Although the helix retains its shape between the two conformations, MD simulations revealed reversible unwinding of the helix that is involved in the functional behavior of FecA. Hence, these FP predictions may be correct. Some more FP breaking contacts reside between the plug domain and the surrounding $\beta$-barrel.

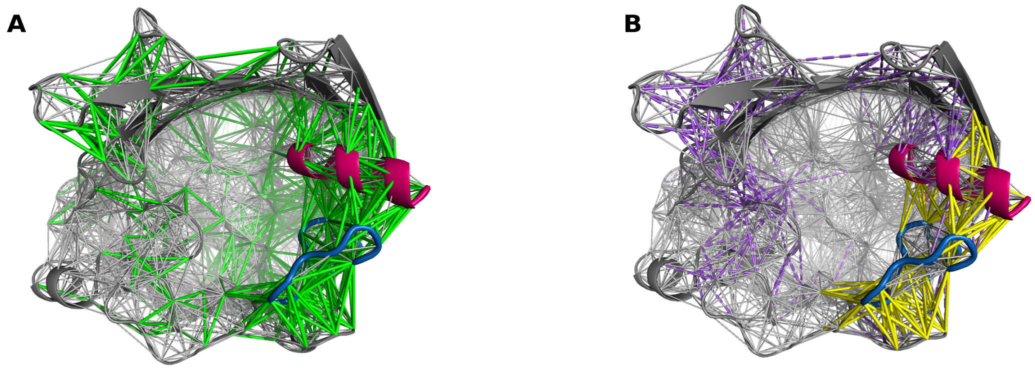


Figure L: **Contact networks for outer membrane transporter FecA based on the optimal extension threshold determined for this protein only**. (A) Top view of the observed breaking contacts identified based on the optimal extension threshold of 3% that maximizes the cumulative mode overlap of the first ten low-frequency modes of this protein. Please note that the optimal extension for our data set is 9%. (B) Location of predicted breaking contacts of *lmc*ENM. Given this stricter extension threshold observed and breaking contacts would agree much better, especially around loops 4 and 5. This indicates that the classifier may has correctly predicted more flexibility in these regions, which is supported by the observed fluctuations for loop 5 in MD simulations.

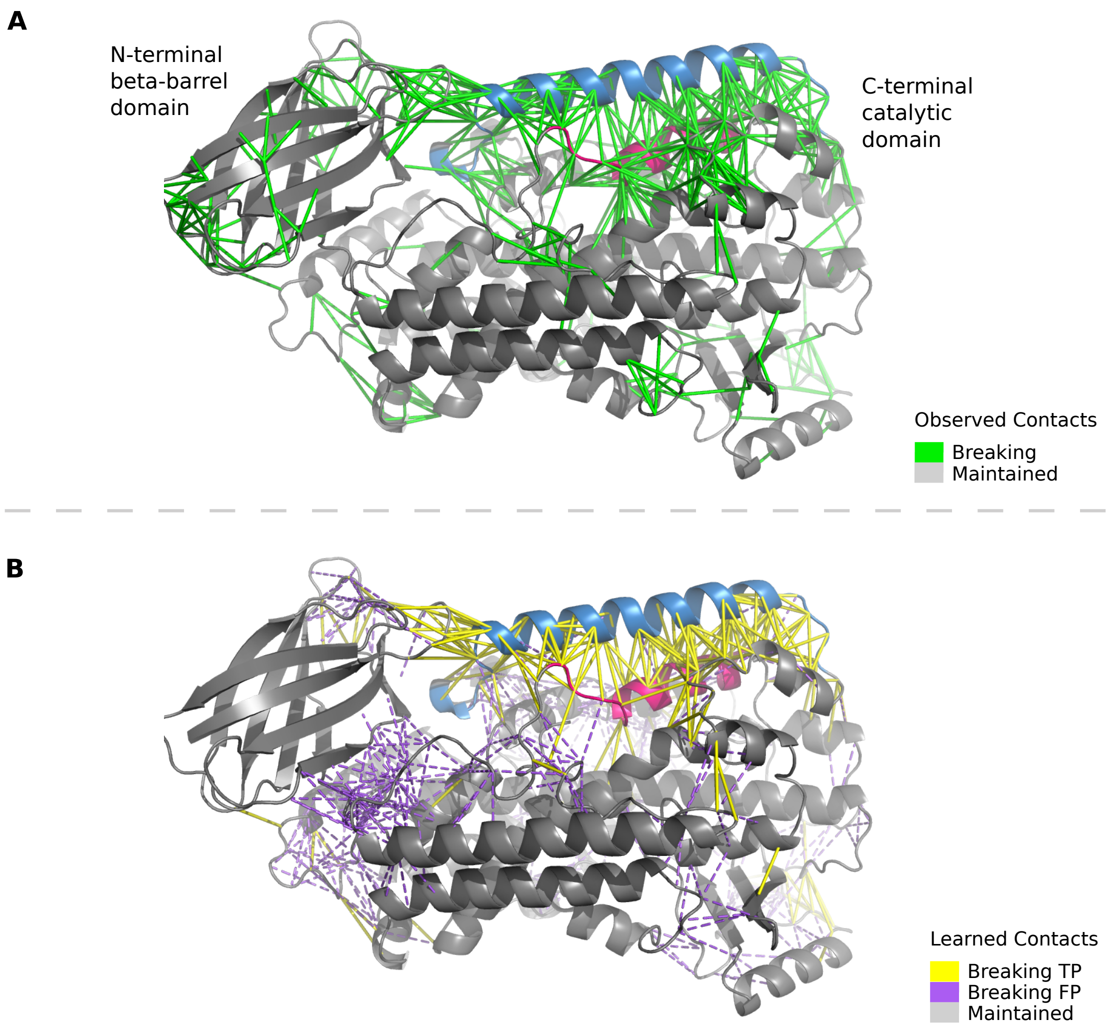


Figure M: **Observed and predicted breaking contacts of Arachidonate 15-Lipoxygenase (side view).** (A) Observed breaking contacts. Most observed breaking contacts reside at the interface of the $\alpha$2-helix (blue) to the rest of the structure. (C) The learned breaking contacts match most of the observed ones near the two helices. The majority of false positive predicted breaking contacts resides between the highly flexible N-terminal $\beta$-barrel domain and the catalytic C-terminal domain. Given the high mobility of the N-terminal domain observed in MD simulations these false positives may actually be correct.

## References


[1] Schneider M, Brock O. Combining Physicochemical and Evolutionary Information for Protein Contact Prediction. PLoS ONE. 2014 Oct;9(10):e108438. Available from: http://dx.doi.org/10.1371/journal.pone.0108438.

[2] Cavallo L, Kleinjung J, Fraternali F. POPS: a fast algorithm for solvent accessible surface areas at atomic and residue level. Nucleic Acids Res. 2003 Jul;31:3364–3366. Available from: http://www.ncbi.nlm.nih.gov/pmc/articles/PMC169007/.

[3] Fischer JD, Mayer CE, Soeding J. Prediction of protein functional residues from sequence by probability density estimation. Bioinformatics. 2008 Mar;24(5):613–620. Available from: http://bioinformatics.oxfordjournals.org/content/24/5/613.

[4] Kim C, Basner J, Lee B. Detecting internally symmetric protein structures. BMC Bioinformatics. 2010 Jun;11:303. Available from: http://www.ncbi.nlm.nih.gov/pmc/articles/PMC2894822/.

[5] Cock PJA, Antao T, Chang JT, Chapman BA, Cox CJ, Dalke A, et al. Biopython: freely available Python tools for computational molecular biology and bioinformatics. Bioinformatics. 2009 Jun;25(11):1422–1423. Available from: http://www.ncbi.nlm.nih.gov/pmc/articles/PMC2682512/.

[6] Hamelryck T. An amino acid has two sides: A new 2D measure provides a different view of solvent exposure. Proteins. 2005 Apr;59(1):38–48. Available from: http://onlinelibrary.wiley.com/doi/10.1002/prot.20379/abstract.

[7] Latapy M, Magnien C, Vecchio ND. Basic notions for the analysis of large two-mode networks. Soc Networks. 2008 Jan;30(1):31–48. Available from: http://www.sciencedirect.com/science/article/pii/S0378873307000494.

[8] Guilloux VL, Schmidtke P, Tuffery P. Fpocket: An open source platform for ligand pocket detection. BMC Bioinformatics. 2009 Jun;10(1):168. Available from: http://www.biomedcentral.com/1471-2105/10/168/abstract.

[9] Frishman D, Argos P. Knowledge-based protein secondary structure assignment. Proteins. 1995 Dec;23:566–579.

[10] Hagberg AA, Schult DA, Swart PJ. Exploring Network Structure, Dynamics, and Function using NetworkX. Proceedings of the 7th Python in Science Conference. 2008;p. 11–15.

[11] Pedregosa F, Varoquaux G, Gramfort A, Michel V, Thirion B, Grisel O, et al. Scikit-learn: Machine Learning in Python. J Mach Learn Res. 2011;12:2825–2830. Available from: http://jmlr.org/papers/v12/pedregosa11a.html.

[12] Chang CC, Lin CJ. LIBSVM: A library for support vector machines. ACM Trans Intell Syst Technol. 2011 May;2(3):27:1–27:27. Available from: http://doi.acm.org/10.1145/1961189.1961199.

[13] Johannessen BR, Skov LK, Kastrup JS, Kristensen O, Bolwig C, Larsen JN, et al. Structure of the house dust mite allergen Der f 2: Implications for function and molecular basis of IgE cross-reactivity. FEBS Letters. 2005 Feb;579(5):1208–1212. Available from: http://onlinelibrary.wiley.com/doi/10.1016/j.febslet.2004.11.115/abstract.

[14] Orellana L, Rueda M, Ferrer-Costa C, Lopéz-Blanco JR, Chacón P, Orozco M. Approaching Elastic Network Models to Molecular Dynamics Flexibility. J Chem Theory Comput. 2010 Sep;6(9):2910–2923. Available from: http://dx.doi.org/10.1021/ct100208e.
